# Constructing porous aromatic frameworks with controllable hierarchical pores to investigate their effect on the adsorption activity of micro-pollutants

**DOI:** 10.1039/d6sc03053j

**Published:** 2026-08-08

**Authors:** Shaolei Wang, Lin Zhao, Man Feng, Zhen Zhan, Kai Ma, Chengxin Zhang, Guangshan Zhu

**Affiliations:** a Key Laboratory of Polyoxometalate and Reticular Material Chemistry of Ministry of Education, Faculty of Chemistry, Northeast Normal University Changchun 130024 China wangsl030@nenu.edu.cn zhugs010@nenu.edu.cn; b Department of Applied Physics, The Hong Kong Polytechnic University Kowloon Hong Kong SAR 999077 China; c School of Materials Science and Engineering, Shenyang Ligong University Shenyang 110159 China

## Abstract

Adsorption activity serves as a key indicator to assess the applicability of materials in micro-pollutant adsorption. Nevertheless, the current understanding of the relationship between hierarchical pores and adsorption activity remains highly ambiguous and insufficient to guide the design and synthesis of porous materials for efficient adsorption of micropollutants. Herein, we report a novel type of sulfonated porous aromatic frameworks (PAFs) with tunable hierarchical pores (especially macropores and micropores) to systematically investigate the relationship between porous structure and micro-pollutant adsorption activity. The hierarchical pores were finely designed and regulated by controlling the amount of water in the aqueous phase and adjusting the porogen-to-monomer volume ratio in the oil phase during high internal phase emulsions (HIPE) polymerization, respectively. The water amount in the aqueous phase helped to optimize porosity and macropore size, and the volume ratio of porogen-to-monomer could modulate the micropore volume and specific surface area of PAF materials. The adsorption data of methylene blue (MB) and tetracycline (TC) suggested that the adsorption rate was independent of specific surface area and micropore volume but increased with increasing macropore size of PAF materials. In contrast, the adsorption capacity was governed by micropore structure rather than macropore size. Moreover, the removal efficiencies of MB and TC exceeded 99% within 10 s, with *k*_2_ values of 2.09 g mg^−1^ min^−1^ and 1.53 g mg^−1^ min^−1^, respectively, which are almost 10–100 times higher than those of other porous adsorbents. This work introduces a straightforward and viable approach to prepare sulfonated POPs materials with breakneck adsorption rate. More importantly, it reveals the inherent relationship between hierarchical pores and the adsorption activity of micro-pollutants.

## Abstract 2

Novel sulfonated porous aromatic frameworks with tunable hierarchical pores were constructed to systematically elucidate the structure–activity relationship governing efficient micropollutant adsorption.

## Graphical abstract

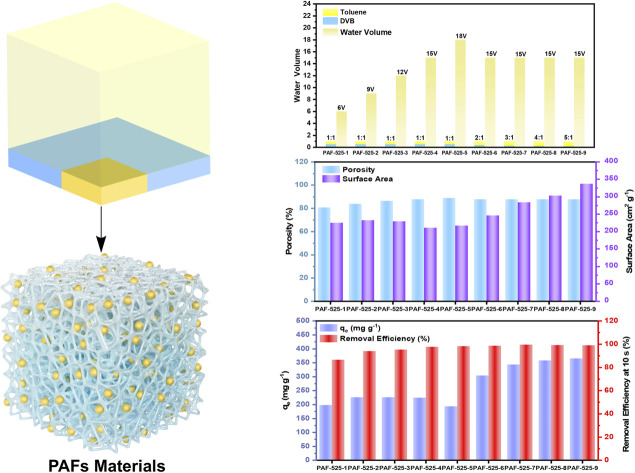

## Introduction

The development of novel adsorbent materials provides an important approach for sustainable water purification through adsorption technology, offering low operating costs, simple process design, high removal efficiency, and reduced secondary pollution compared with most other pollutant-removing methods.^1^ Among adsorbent materials, porous materials such as porous carbon, metal–organic frameworks (MOFs) and porous organic polymers (POPs) with high specific surface area and designable structure facilitate the introduction and exposure of many more adsorption sites and accelerate the rate of mass transfer, thus presenting broad application prospects in the field of water purification.^2–4^ It is necessary to consider the adsorption rate, adsorption selectivity, and adsorption capacity for practical applications of porous materials. However, most porous materials reported usually lack good selectivity for micro-pollutants, which hinders their application in real-world scenarios. The introduction of adsorption sites is an effective strategy for improving adsorption selectivity, but it inevitably induces pore narrowing or even blockage in porous adsorbents with a single type of pore, thereby compromising the adsorption rate and capacity.^5^

Numerous studies have demonstrated that hierarchical pores have the potential to overcome the limitations of single pores by reducing pore blockage, shortening the transmission path, enhancing diffusion kinetics, and improving site accessibility during adsorption processes.^6,7^ The reason is that micropores can provide and expose adsorption sites, and mesopores serve as channels to connect micropores, facilitating the access of micro-pollutants to adsorption sites. Macropores act as transport pathways to improve overall mass transfer efficiency, which is crucial for increasing the adsorption rate.^8^ For example, Zhou *et al.* introduced tunable hierarchical pores into PCN-250 by using a modulating method and templating method, and PCN-250 with surface and internal pores showed excellent dye-removal performance compared with PCN-250 with only micropores, which could be attributed to surface-pore blocking.^9^ The relationship between hierarchical pores and adsorption activity is ambiguous because the studies related to this relationship lack elaborate analyses and systematic investigation, which prevents the rational design and synthesis of porous adsorbents with efficient adsorption performance.^10^ Furthermore, the existing methods of constructing hierarchical pores often involve high fabrication costs and complex preparation processes, rendering these methods impractical and difficult to implement in an industrial setting.^6^

Herein, we report a novel type of sulfonated porous aromatic framework (PAF) with tunable hierarchical pores, especially macropores and micropores, to investigate the effect of hierarchical pores on micro-pollutant adsorption activity. The hierarchical pores of these PAFs materials were systematically designed and regulated by adjusting the amount of water in the aqueous phase and controlling the volume ratio of porogen : monomer in the oil phase during high internal phase emulsion (HIPE) polymerization, respectively. Analyses of porous structure showed that the amount of water in the aqueous phase helped to optimize porosity and macropore size, and the volume ratio of porogen : monomer could alter the micropore volume and specific surface area of PAFs materials. Meanwhile, rich mesopores and interconnected macropores made sulfonation modifications more thorough, endowing PAF materials with high sulfur content. The tunable hierarchical pores and constant sulfonic acid content could ensure that the relationship between hierarchical pores and the adsorption activity of micro-pollutants could be studied systematically and objectively. The adsorption data of methylene blue (MB) and tetracycline (TC) suggested that the adsorption rate of micro-pollutants was independent of specific surface area and micropore volume, yet positively correlated with the macropore size of PAFs materials, whereas the adsorption capacity showed opposite trends relative to the adsorption rate. Moreover, the removal efficiency of MB and TC exceeded 99% within 10 s, with *k*_2_ values of 2.09 g mg^−1^ min^−1^ and 1.53 g mg^−1^ min^−1^, respectively, which is almost 10–100 times higher than those of most porous adsorbents. Thus, this work proposes a simple and effective method to prepare porous materials with regulated hierarchical pores and reveals the inherent influence of pore structure on the adsorption activity of micro-pollutants.

## Experimental section

### Materials

Toluene (Aldrich, 99% grade), divinylbenzene (DVB, Aldrich, 80% grade), and potassium persulfate (K_2_S_2_O_8_, Fisher Scientific) were recrystallized from deionized water prior to drying under reduced pressure. ClSO_3_H (anhydrous) and other reagents of analytical grade were used without further purification and are obtained from National Medicines Corporation Ltd of China.

### Synthesis of PAM precursors

An oil phase consisting of 0.7 mL DVB, 0.7 mL of a porogen (toluene) and 0.21 g of Span 80 was stirred for 2 h under continuous mechanical stirring (300 rpm). Subsequently, a aqueous phase containing 0.013 g of K_2_S_2_O_8_, 0.067 g of CaCl_2_ and a certain amount of H_2_O was added dropwise at 1.5 mL min^−1^. After the dropwise addition of the aqueous phase, the high internal phase emulsion was polymerized at 60 °C for 48 h.^11^ Samples were further extracted with ethanol using Soxhlet extraction for 2 day and freeze-dried for 1 day. The porogen-to-DVB volume ratio in the oil phase was 1, and the water amount in the aqueous phase was 6 mL, 9 mL, 12 mL, 15 mL or 18 mL. The corresponding samples were named ‘PAM-*X*-1’ (where *X* is the water amount). Moreover, the volume ratio of porogen-to-DVB in the oil phase was varied to obtain corresponding samples under a fixed water amount of 15 mL, which were named as ‘PAM-15-*Y*’ (where *Y* is the volume ratio of porogen-to-DVB).

### Synthesis of sulfonated PAFs materials

First, 1 g of PAM and 50 mL of DCE were added to a 100 mL round-bottom flask to thoroughly swell the porous framework for 1 day. Then, 2.5 mL of ClSO_3_H was added dropwise to the reaction system in an ice-water bath, followed by stirring at room temperature for 1 day to complete sulfonation. Finally, the obtained sample was quenched in ice water, washed with distilled water to neutral pH, extracted with ethanol for 2 day, and then dried under vacuum at 60 °C to obtain sulfonated PAFs. Other PAM materials were processed according to the same dosage and reaction conditions. The corresponding materials of PAM-*X*-1 were named ‘PAF-525-1’ to ‘PAF-525-5’, and those of PAM-15-*Y* were named ‘PAF-525-4’ and ‘PAF-525-6’ to ‘PAF-525-9’, respectively.

## Results and discussion

**Fig. 1:**
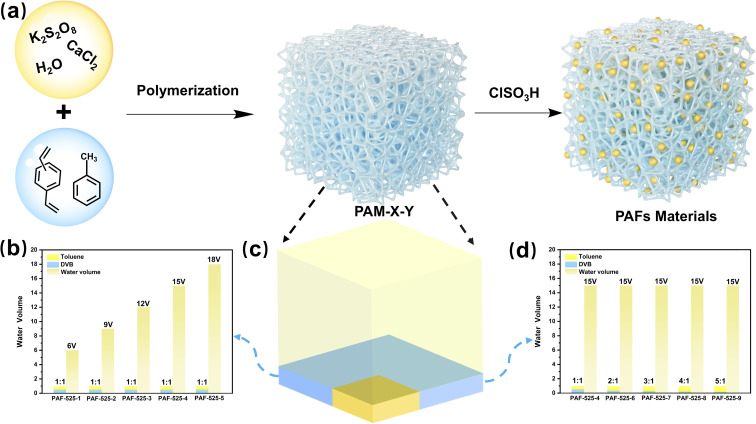
(a) Synthetic procedure of sulfonated PAFs materials (blue bubbles represent the oil phase, and yellow bubbles represent the aqueous phase). (b) Regulation of the water amount of the aqueous phase under a fixed volume ratio of toluene-to-DVB of the oil phase. (c) HIPE composition (schematic). (d) Adjustment of the volume ratio of toluene-to-DVB under a fixed water amount of the aqueous phase.

The resulting samples were synthesized by HIPE polymerization and further sulfonation using DVB and ClSO_3_H as the polymerization monomer and sulfonating reagent, respectively (Fig. 1a). The hierarchical pores of these resulting samples were finely designed and regulated by adjusting the water amount in the aqueous phase and controlling the volume ratio of porogen-to-monomer in the oil phase during HIPE polymerization, which facilitated systematic investigation of the influence of hierarchical pores upon adsorption activity (Fig. 1b–d). First, five water amounts of the aqueous phase were chosen to regulate the macropore structure of frameworks under the fixed volume ratio of porogen-to-monomer in the oil phase (Fig. 1b and S1), and five monoliths were obtained and named as ‘PAF-525-1’ to ‘PAF-525-5’. Subsequently, the volume ratio of porogen-to-monomer of the oil phase was regulated further to investigate the variation of micropores, and we obtained four additional monoliths (PAF-525-6 to PAF-525-9) under the fixed water amount of the aqueous phase (Fig. 1d). Finally, the relationship between porous structure and activity of micro-pollutants was studied in detail.

The chemical structure of these resulting samples was determined by Fourier transform infrared (FT-IR) spectroscopy, solid-state ^13^C CP/MAS nuclear magnetic resonance (NMR) spectroscopy, and X-ray photoelectron spectroscopy (XPS). Taking a precursor of PAF-525-9 as an example, the signals near 1633 cm^−1^ and 1453 cm^−1^ indicated the presence of C=C vibration peaks of aromatic carbon, whereas the broad peaks at 2800–3000 cm^−1^ corresponded to the saturated C–H vibration peaks of alkyl groups.

These observations implied that the ethylene groups of monomers underwent free–radical reaction to form polymer networks^12^ (Fig. S2 and S3). After the sulfonation reaction, PAF-525-9 showed two new peaks at 1172 cm^−1^ and 1039 cm^−1^, which were ascribed to the asymmetric stretching vibrations and symmetric stretching vibrations of the O=S=O bond,^13^ while the adsorption peaks at 912 cm^−1^ and 707 cm^−1^ were assigned to S–OH and C–S bonds, respectively^14^ (Fig. S4). The emergence of characteristic peaks demonstrated that sulfonic acid groups had been introduced into polymer frameworks (Fig. S3–S6).

As shown in Fig. S7a, the resonance peaks near 140 and 123 ppm could be ascribed to the substituted aromatic carbon and unsubstituted aromatic carbon, respectively. Also, the carbon signal of methylene linkers appeared near 35 ppm stemming from the self-crosslinking of vinyl groups for precursors.^15^ Compared with the precursor, the introduction of sulfonic acid groups exerted an indicative effect on the carbon atoms of the benzene ring, resulting in a shift of the nuclear magnetic carbon spectrum signal and significant enhancement of the model between 124 ppm and 139 ppm for PAF-525-9 (Fig. S7b). Different from the precursor, the peaks of S 2s and S 2p appeared near 232.1 eV and 169.1 eV for PAF-525-9, respectively (Fig. S8–S9a). The high-resolution XPS spectrum revealed two peaks at 168.4 eV and 169.4 eV, which were attributed to S 2p_1/2_ and S 2p_3/2_ of O=S=O bonds, respectively (Fig. S9b).^14^ Moreover, the sulfur content of these resulting samples was further quantified by energy dispersive X-ray spectroscopy (EDS) elemental analysis, which showed that the sulfur content reached up to 10.5 wt% (Table S1). FT-IR spectroscopy, XPS, EDS and solid-state ^13^C NMR data confirmed that HIPE polymerization and further sulfonation were effective to construct polymer networks from monomers and introduce sulfonic acid groups into a porous framework.

**Fig. 2:**
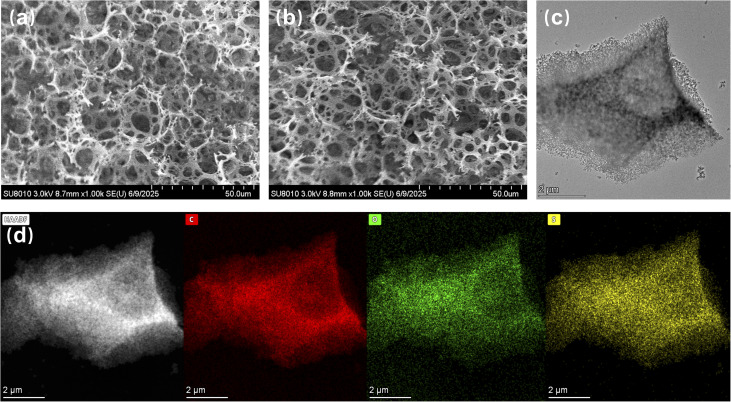
FE-SEM of (a) PAM-15-5 and (b) PAF-525-9. (c) HR-TEM and (d) high-angle annular dark field (HAADF) mapping of PAF-525-9.

The morphology of these resulting samples was investigated by scanning electron microscopy (SEM) and transmission electron microscopy (TEM). The resulting samples showed a macropore size distribution and spanned tens of micrometers, which originated from the removal of the aqueous phase. Macropore size increased with an increase in the water amount during HIPE polymerization for these resulting samples (Fig. S10). However, the change in the volume ratio of porogen-to-monomer in the oil phase did not affect the apparent pore structure of these resulting samples (Fig. S11). Meanwhile, SEM revealed that the macroporous structure of precursors was retained after sulfonation (Fig. 2a and b). Besides, TEM showed that these resulting samples possessed an amorphous structure and disordered pores (Fig. 2c and S12–S16). Three elements were uniformly distributed throughout the framework structure observed by element distribution mapping, which suggested the sulfonation reaction had proceeded (Fig. 2d). The hydrophilicity of PAFs materials was evaluated by water contact angle measurements. Compared with the precursor, the hydrophilicity of PAF-525-9 was significantly enhanced due to the grafting of sulfonic acid groups and increase in charge density (Fig. S17). The self-polymerization of monomers endowed precursors with a uniform chemical structure, thereby showing excellent thermal stability and rapid pyrolysis at 347 °C (Fig. S18). Moreover, the introduction of sulfonic acid groups reduced the stability but increased the residual amount for PAFs materials, whether translated in an air atmosphere or a nitrogen atmosphere (Fig. S18–S19).

**Fig. 3:**
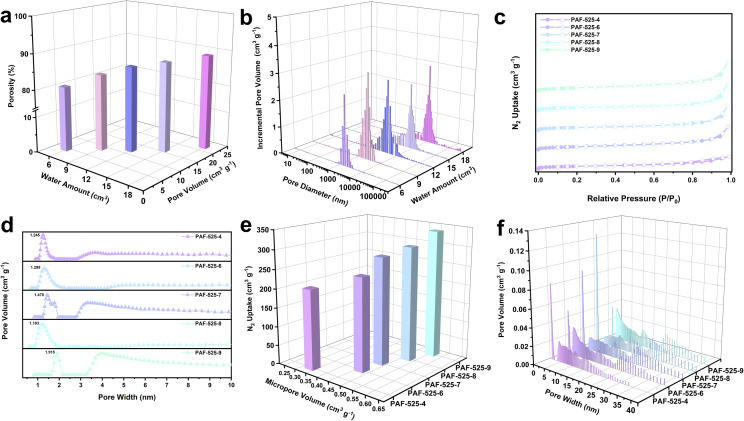
(a) Relationship between porosity, total pore volume and water amount of PAFs materials measured by the mercury intrusion method. (b) Relationship between the incremental pore volume, corresponding pore size, and water amount of PAF materials. (c) N_2_ adsorption–desorption isotherms of PAF-525-4 and PAF-525-6 to PAF-525-9 at 77.3 K. (d) Pore size distributions of PAF-525-4 and PAF-525-6 to PAF-525-9 calculated using density functional theory (DFT) methods. (e) Change in specific surface area and micropore volume and (f) change in the incremental pore volume and corresponding pore size of PAF materials by regulating the volume ratio of porogen-to-monomer of the oil phase.

The pore structure of the resulting samples was evaluated using the mercury injection method and nitrogen sorption method. The cumulative pore volume increased with increasing pressure and reached an adsorption equilibrium at 1000 bar for all PAFs materials. Notably, the increase in water amount led to an increase in the porosity and total pore volume for PAFs materials from 80.6% to 88.9% and from 4.3 mL g^−1^ to 21.8 mL g^−1^, respectively (Fig. 3a). Meanwhile, a higher water amount made the droplet volume of the aqueous phase much larger, thereby promoting an increase in macropore size, and the cumulative pore volume, porosity and pore size distribution were maximized when the water amount was 18 mL (Fig. 3a, b and Table S2). Furthermore, a rapid increase in the nitrogen adsorption capacity in the low-pressure region indicated that PAFs materials had abundant micropores and a high specific surface area based on the International Union of Pure and Applied Chemistry (IUPAC) classification (Fig. 3c and S20a–S22a). The pore size distribution further confirmed that PAFs materials possessed a hierarchical pore structure with abundant micropores (Fig. 3d and S20b–22b). The Brunauer–Emmett–Teller (BET) surface areas of PAFs materials are shown in Table S3. The micropore volume and specific surface area remained essentially unchanged for PAF-525-1 to PAF-525-5, which stemmed from the fixed volume ratio of porogen-to-monomer of the oil phase. However, the micropore volume and specific surface area increased with an increase in the volume ratio of porogen-to-monomer for PAF-525-4 and PAF-525-6 to PAF-525-9 (Fig. 3e and f). These results suggested that the water amount in the aqueous phase helped to optimize porosity and macropore size, and the volume ratio of porogen-to-monomer could modulate the micropore volume and specific surface area of PAF materials. Moreover, the introduction of sulfonic acid groups caused a substantial decrease in the specific surface area and micropore volume compared with precursors (Table S3).^16–19^ Based on analyses of pore structure, the fine design and regulation of porous structure had been achieved for PAFs materials, which would be conducive to studying the relationship between hierarchical pores and the adsorption activity of micro-pollutants.

**Fig. 4:**
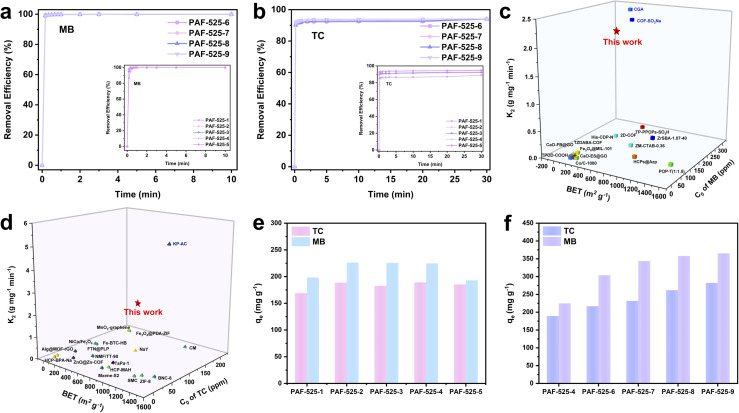
Time-dependent adsorption of MB (a) and TC (b) at different concentrations by PAFs materials. Comparison of BET surface area, *k*_2_, and the concentration of MB (c) and TC (d) of PAF-525-9 and a porous adsorbent. (e) Equilibrium adsorption capacities of MB and TC by PAF-525-1 to PAF-525-5. (f) Equilibrium adsorption capacities of MB and TC by PAF-525-4, and PAF-525-6 to PAF-525-9.

Subsequently, we systematically studied the effects of porous structure on micro-pollutants adsorption activity, considering that PAF materials possess a designable porous structure, excellent hydrophilicity and rich sulfonic acid groups. MB and TC were employed as research models to investigate micro-pollutants adsorption performance (Fig. S23). The removal efficiencies of micro-pollutant models at different time intervals are illustrated in Fig. 4a and b. PAF materials showed a rapid adsorption rate for micro-pollutant models in a 200 mg L^−1^ MB aqueous solution. Interestingly, the MB removal efficiency of PAF-525-1 to PAF-525-5 reached 86.56%, 93.91%, 95.30%, 97.63% and 98.22% within 10 s, respectively. Among these samples, PAF-525-5 achieved an MB removal rate of 99.9% within 20 s, while PAF-525-1, PAF-525-2, PAF-525-3, and PAF-525-4 exhibited MB removal rates of 94.9%, 98.8%, 99.0% and 99.8%, respectively. However, the MB removal efficiency remained essentially unchanged under the increase of surface area and micropore volume for PAF-525-4 and PAF-525-6 to PAF-525-9 (Fig. 4a). Meanwhile, the adsorption rate of TC showed the same pattern as that of MB for PAF-525-1 to PAF-525-9, which could effectively and swiftly remove TC, achieving adsorption equilibrium within only 5 min (Fig. 4b). Moreover, the pseudo-first-order and pseudo-second-order modes were employed to evaluate adsorption kinetics data (Fig. S24–S27). The pseudo-second-order model fitted the adsorption kinetics better than the pseudo-first-order model, as judged by the coefficient of determination (*R*^2^), suggesting that chemisorption was the rate-determining step for the adsorption process of these micro-pollutants.^20–22^ The pseudo-second-order rate constant (*K*_2_) of MB and TC was 2.09 and 1.53 g mg^−1^ min^−1^, respectively, much higher than those of most porous adsorbents (Fig. 4c, d and Tables S4, S5). These results collectively suggested that the adsorption rate of micro-pollutants was independent of specific surface area and micropore volume, but positively correlated with the macropore size of porous adsorbents. The reason is that larger and richer macropores facilitate efficient mass transfer, thereby accelerating the diffusion of micro-pollutants to adsorption sites.

Subsequently, the intraparticle diffusion model based on the Weber–Morris equation was further employed to investigate the rate-determining step during the MB and TC adsorption process.^23^ For PAF-525-1 to PAF-525-5, the low specific surface area and abundant hierarchical pore structure shortened the diffusion path of micro-pollutants towards the adsorption sites. This phenomenon led to the intraparticle diffusion curves of PAF-525-1 to PAF-525-5 deviating from the classical three-stage adsorption process, instead showing a two-stage adsorption process for MB and TC adsorption (Fig. S28–S29). Meanwhile, the fitting curve of each stage did not pass through the origin, which indicated that intraparticle diffusion was not the sole rate-determining step. In other words, the adsorption processes of MB and TC were jointly controlled by multiple diffusion steps.^24,25^ With increasing micropore volume and specific surface area, the transport pathways of MB and TC within the pore were extended, which resulted in the appearance of a distinct three-stage adsorption process of PAF-525-6 to PAF-525-9 toward MB and TC (Fig. S30–S31). The first linear stage corresponds to MB and TC diffusion from the bulk solution to the external surface of PAF-525-6 to PAF-525-9 *via* boundary layer diffusion, while the second stage reflects the intraparticle diffusion of MB and TC from the external surface into the internal pore of PAF-525-6 to PAF-525-9. The *k*_i2_ of the second stage decreased with increasing micropore volume and specific surface area, suggesting that the increase in micropore volume reduced intraparticle diffusion efficiency (Table S6).

The relationship between maximum adsorption capacity (*q*_m_) and porous structure was further estimated by measuring the adsorption capacity of micro-pollutants at various equilibrium concentrations (Fig. S32). Meanwhile, the consequences of micro-pollutant adsorption were fitted by Langmuir and Freundlich adsorption isotherm models (Fig. S33).^26^ The *R*^2^ of the Langmuir isotherm model was significantly higher than that of the Freundlich isotherm model, which suggested that the adsorption mechanism was much more consistent with the characteristics of monolayer and uniform adsorption (Table S7).^27–29^ With an increase in the total pore volume, porosity and macropores size of PAF-525-1 to PAF-525-5, their theoretical maximum adsorption capacities of MB were relatively constant, with values of 198 mg g^−1^, 226 mg g^−1^, 225 mg g^−1^, 224 mg g^−1^ and 192 mg g^−1^, respectively (Fig. 4e). However, the theoretical maximum adsorption capacities of PAF-525-4, and PAF-525-6 to PAF-525-9 increased with an increase in the surface area and pore volume under the condition of essentially constant macropore size and sulfonic acid content, which were 224 mg g^−1^, 303 mg g^−1^, 343 mg g^−1^, 357 mg g^−1^ and 365 mg g^−1^, respectively (Fig. 4f). Meanwhile, the maximum adsorption capacity of TC showed the same pattern as that of MB for PAF-525-1 to PAF-525-9, and PAF-525-9 showed the highest adsorption capacity of 282 mg g^−1^ for TC. These results demonstrated that the maximum adsorption capacity of micro-pollutants was dependent upon the specific surface area and micropore volume of PAF materials rather than macropore size.^10^ This phenomenon could be because a larger specific surface area and greater micropore volume provide more adsorption sites, thereby enhancing the adsorption capacity of PAF materials. Moreover, the maximum absorption capacities of MB and TC were higher than those of most porous adsorbents, such as AC,^30^ HCPs@Azo,^31^ Ni-MOF,^32^ and His-CDP-N.^33^ The above research clearly clarified the relationship between hierarchical pores and the adsorption activity of micro-pollutants. That is, the adsorption rate is dependent upon macropore size, and the maximum adsorption capacity is determined by the specific surface area and micropore volume.

The correlation between sulfonic acid density and the adsorption capacity of the resulting samples was systematically investigated. As the sulfonation degree of PAM-15-5 decreased, the sulfonic acid density of the resulting samples declined significantly, whereas their specific surface area and micropore volume exhibited a notable upward trend (Tables S1, S3 and Fig. S34). The maximum adsorption capacities of the resulting samples for MB and TC decreased with decreasing sulfonic acid density. In contrast, the surface area and micropore volume increased with decreasing sulfonic acid density (Table S7 and Fig. S35–S38). This result suggested that sulfonic acid density had a more important role than their porosity in determining the maximum adsorption capacity of the resulting samples toward micropollutants. Moreover, the influence of the mesoporous structure of PAFs on their adsorption performance towards MB and TC was also briefly investigated. Meso-PAF-525-4 was synthesized *via* the hard-template method. Compared with PAF-525-4, Meso-PAF-525-4 exhibited an increased specific surface area and a distinct mesoporous structure centered at 50 nm (Fig. S39). The introduction of mesopores increased the specific surface area of Meso-PAF-525-4, thereby improving its maximum adsorption capacity towards MB and TC. However, the introduction of mesopores exerted no significant effect on the adsorption kinetics, which may be because the newly introduced mesopores could not notably alter the mesopore-to-macropore ratio of Meso-PAF-525-4 (Fig. S40).

Meanwhile, the Gibbs free energy (Δ*G*^0^) and activation energy (*E*_a_) were calculated to gain in-depth insights into the adsorption mechanism. The Gibbs free energy was −20.82 kJ mol^−1^ for MB and −16.16 kJ mol^−1^ for TC, respectively. Negative Δ*G*^0^ values indicate that MB and TC could spontaneously adsorb onto the adsorption sites of PAF-525-9.^34^ According to the literature, physisorption typically exhibits low *E*_a_ values (lower than 4.2 kJ mol^−1^), whereas chemisorption corresponds to higher *E*_a_ values (8.4–83.7 kJ mol^−1^). The activation energy was 79.96 kJ mol^−1^ for MB and 34.48 kJ mol^−1^ for TC based on the Arrhenius equation, which fell precisely within the typical range for the chemisorption of micro-pollutants (Fig. S41–S43 and Table S8). This result indicated that the adsorption of PAF-525-9 towards MB and TC was dominated by chemisorption. Moreover, the positive value for the activation energy further indicated that the adsorption process was endothermic and thus thermodynamically favored at higher temperatures.

Subsequently, theoretical simulations were carried out by density functional theory (DFT) calculations to further clarify the interaction mechanisms between PAF-525-9 and micro-pollutants (Fig. S44–S46). As shown in Fig. S45, blue and red regions represented the minimum and maximum electrostatic potential (ESP) values of each structure, respectively. The minimum ESP regions of PAF-525-9 were predominantly distributed on its sulfonic acid groups, while the MB molecule exhibited overall positive ESP characteristics. For TC, the minimum ESP values were mainly localized on benzene ring moieties, and the maximum ESP values were concentrated on amino (–NH_2_) and hydroxyl (–OH) groups. As illustrated by the IGMH analysis (Fig. S47), the intermolecular interactions between PAF-525-9 and micro-pollutants were dominated by electrostatic interactions. The calculated binding energy of TC/PAF-525-9 and MB/PAF-525-9 complexes was −143.56 kJ mol^−1^ and −160.26 kJ mol^−1^, respectively, indicating a stronger binding affinity of PAF-525-9 towards MB than TC (Table S9). This trend was consistent with the experimental observation that the adsorption performance of PAF-525-9 towards MB was superior to that toward TC.

Multiple characterization methods were adopted to elucidate the intrinsic adsorption mechanism of PAF-525-9 towards MB and TC. The FT-IR spectra of PAF-525-9@MB and PAF-525-9@TC displayed distinct characteristic absorption bands that were uniquely assigned to MB and TC molecules, respectively (Fig. S48 and S49). These results provided direct spectroscopic evidence for the adsorption of MB and TC within PAF-525-9 frameworks, which was further verified by the emergence of the N 1s signal in the corresponding XPS spectrum, showing the obvious change in the pore structure and prolonged water wetting time for PAF-525-9@MB and PAF-525-9@TC (Table S3 and Fig. S50–S53). Importantly, the S=O bond stretching vibration peaks, as well as the S 2p_3/2_ and S 2p_1/2_ peaks of PAF-525-9@MB and PAF-525-9@TC, showed slight shifts compared with those of PAF-525-9 (Fig. S48–S51). These spectral perturbations originated from the strong electrostatic attraction between cationic micropollutants and anionic sulfonic acid sites, which introduced an external electric field around the sulfonic acid groups and thereby altered the chemical microenvironment and vibrational frequency of the S=O bonds.^35^ Compared with pristine PAF-525-9, the zeta potential of PAF-525-9@MB and PAF-525-9@TC exhibited a distinct positive shift, rising from −37.2 mV to −31.8 mV and from −37.2 mV to −32.4 mV, respectively (Fig. S54). This phenomenon could be reasonably attributed to the electrostatic adsorption of cationic MB and TC molecules onto the anionic sulfonic acid sites within the porous framework, which partially neutralized the intrinsic negative surface charge of the polymeric network.^36,37^ A combination of adsorption data, theoretical calculation results and multi-dimensional characterization evidence confirmed that electrostatic interactions were the dominant driving force for the adsorption of MB and TC by PAF-525-9.

Given the optimal adsorption performance of PAF-525-9, we further investigated its practical applications of adaptability and reusability. The maximum adsorption capacity of MB remained relatively consistent across different pH values for PAF-525-9. By contrast, the maximum adsorption capacity of TC initially increased and then decreased with a change in pH, with optimal adsorption achieved at a pH of 6.^38,39^ The solution pH influenced the charge state and molecular conformation of TC and modulated the surface charge characteristics of sulfonic acid groups, which collectively governed the change in adsorption capacity of PAF-525-9 towards TC (Fig. S55).^40–42^ The adsorption capacity of MB and TC increased with an increase in temperature, which suggested that the adsorption process between PAF-525-9 and micro-pollutants was endothermic (Fig. S56).^43–45^ More importantly, PAF-525-9 showed excellent removal performance at low micro-pollutant concentrations (1 mg L^−1^ MB and 1 mg L^−1^ TC), achieving a removal efficiency of 98% for MB and 98% for TC, respectively (Fig. S57).

Meanwhile, tap water was further employed as the solvent to investigate the adsorption performance of PAF-525-9 towards micro-pollutants in real aqueous environments. The use of tap water as the solvent slightly decreased the MB adsorption performance of PAF-525-9, yet significantly reduced its TC adsorption performance (Fig. S58a and b). Notably, the adsorption performance of PAF-525-9 for ciprofloxacin (CIP) exhibited no significant change in tap water solution (Fig. S58c). This phenomenon could have been because calcium and magnesium ions can complex with TC, thereby weakening the adsorption performance of PAF-525-9 for TC.^46^ The adsorption performance of PAF-525-9 towards TC was notably restored when using EDTA-treated tap water as the solvent, further validating this mechanism (Fig. S58d and S59). Moreover, PAF-525-9 displayed outstanding resistance to interference from sodium ions. The adsorption performance of PAF-525-9 towards MB and CIP decreased slightly with an increase in sodium ion concentration, whereas the adsorption performance of PAF-525-9 for TC declined significantly (Fig. S60 and S61). This result further confirmed that TC was more susceptible to metal ion interference than MB and CIP, which consequently impaired its adsorption behavior.

MB and methyl orange (MO) were chosen as representative cationic and anionic dyes, respectively, to assess the adsorption selectivity of PAF-525-9. As shown in Fig. S62a, the solution color gradually changed from dark green to the characteristic color of MO within 2 min, suggesting that PAF-525-9 preferentially adsorbed cationic dyes over anionic dyes (Fig. S62b). Meanwhile, the characteristic peaks of MB at 465 nm disappeared completely, whereas the characteristic peaks of MO remained essentially unchanged. The removal efficiency of MB exceeded 99%, which could be ascribed to the strong electrostatic interactions between the sulfonic acid groups of PAF-525-9 and cationic dyes. Moreover, PAF-525-9 showed high cycle stability and maintained superior adsorption performance of micro-pollutant models after five cycles (>98%) (Fig. S63). The chemical and morphological structure of PAF-525-9 remained constant after recycling (Fig. S64 and S65). Based on systematic design and regulation of porous structure, PAF-525-9 showed an ultra-rapid adsorption rate, high absorption capacity, excellent universality and good reusability for MB and TC. The *k*_2_ values of MB and TC were found to be 2.09 g mg^−1^ min^−1^ and 1.53 g mg^−1^ min^−1^, respectively, which were almost 10–100 times higher than those of other porous materials and superior to those of most micro-pollutant adsorbents.^47–50^ That is, PAF-525-9 has great potential in water treatment and could contribute to the protection of the ecological environment.

## Conclusions

A series of sulfonated PAF materials with adjustable porous structure, porosity, and pore size was prepared by HIPE polymerization and subsequent sulfonation. The porous structure of these PAF materials was designed and regulated readily by controlling the water amount in the aqueous phase and adjusting the porogen-to-monomer volume ratio in the oil phase during HIPE polymerization, respectively. The adjustable pores enabled accurate elucidation of the relationship between hierarchical pores and the adsorption activity of micro-pollutants. The adsorption rate was dependent upon macropore size. The maximum adsorption capacity was determined by the specific surface area and micropore volume. Hence, a larger and richer macropore size could provide much higher mass transfer efficiency, thereby improving the adsorption rate of porous adsorbents. Meanwhile, the specific surface area and micropore volume exerted a greater impact on the adsorption capacity of micro-pollutants due to exposure to more adsorption sites. The introduction of a mesoporous structure could enhance the maximum adsorption capacity of PAF materials toward MB and TC, while exerting a negligible influence on their adsorption kinetics. Moreover, these PAF materials showed an ultra-rapid adsorption rate of MB and TC, with *k*_2_ values of 2.09 and 1.53 g mg^−1^ min^−1^, respectively, outperforming those of most reported porous adsorbents. Hence, this study presents a straightforward and viable approach to prepare sulfonated POPs materials with extremely high adsorption rates. More importantly, it reveals an effective and feasible method for investigating the relationship between the pore structure and adsorption activity of micro-pollutants, laying a foundation for the rational design of high-performance porous adsorbents for water treatment.

## Author contributions

Lin Zhao: writing (original draft) and conceptualization. Zhen Zhan and Kai Ma: methodology, data analyses/curation. Chengxin Zhang and Feng Man: validation, investigation. Shaolei Wang and Guangshan Zhu: resources, writing (review and editing), project administration, funding acquisition, supervision.

## Conflicts of interest

The authors declare that they have no known competing financial interests or personal relationships that could have appeared to influence the work reported in this paper.

## Supplementary Material

SC-OLF-D6SC03053J-s001

## Data Availability

The data supporting the conclusions reached from our study have been uploaded as part of the supplementary information (SI), which are also available from the corresponding authors upon reasonable request. Supplementary information is available. See DOI: https://doi.org/10.1039/d6sc03053j.
