# Supplementary material for: Constructing porous aromatic frameworks with controllable hierarchical pores to investigate their effect on the adsorption activity of micro-pollutants
Source: Chem Sci. 2026 Aug 8;17(39):18722–31. doi: 10.1039/d6sc03053j (PMC13482920; doi:10.1039/d6sc03053j)
Supplement: Supplementary file 1 [file SC-017-D6SC03053J-s001.pdf]

Supplementary Information (SI) for Chemical Science.
This journal is © The Royal Society of Chemistry 2026

Supporting Information

Constructing porous aromatic frameworks with controllable hierarchical pores to investigate their effect on adsorption activity of micro-pollutants

Shaolei Wang[a*], Lin Zhao[a], Man Feng[a], Zhen Zhan[b], Kai Ma[a], Chengxin Zhang[c], Guangshan Zhu[a*]

[a] Key Laboratory of Polyoxometalate and Reticular Material Chemistry of Ministry of Education, Faculty of Chemistry, Northeast Normal University, Changchun, 130024, China.

[b] Department of Applied Physics, The Hong Kong Polytechnic University, Kowloon, 999077, Hong Kong SAR, China.

[c] School of Materials Science and Engineering, Shenyang Ligong University, Shenyang, 110159, China.

*Corresponding Author:

Prof. Guangshan Zhu.

E-mail: zhugs@nenu.edu.cn.

Table of Contents

Characterization and Preparation..1

Supporting Figures……………………………………………………………………………………5

Supporting Tables…………………………………………………………….……………………….…..24

References…………………………………………………………………………………………...……29

Characterization:

Fourier transform infrared spectroscopy (FT-IR) is measured with KBr pellets via NICOLET iS50. ^{13}C Solid-state NMR is measured on a BRUKER AVANCE NEO 400WB NMR spectrometer. The elements chemical state of the material is analyzed by X-ray photoelectron spectroscopy (Thermo ESCALAB 250XI). Pore size distribution and porosity of materials are measured by a high-performance fully automatic mercury intrusion instrument (AutoPore Iv 9510). The nitrogen adsorption-desorption isotherms are measured on a Quantachrome AutosorbiQ at 77.3 K, and the pore size distribution (PSD) is obtained according to the NLDFT calculation model. Thermo-gravimetric analysis (TGA) is measured on TGA/DSC 3^+ thermalanalyzer in the temperature range of 30-800 ℃ with a heating rate of 10 ^oC min^{-1} under N_2 and air atmosphere, respectively. Field emission scanning electron microscopic analysis (FE-SEM) is performed on a HITACHI SU8010 microscope. Sulfur content is measured through Elementar UNICUBE. The UV-Vis adsorption spectroscopy is measured through the Agilent VARIAN Cary-60 UV-Vis spectrophotometer in the wavelength range of 200-800 nm.

Adsorption kinetics:

MB and TC are selected as the micro-pollutant models for adsorption experiments at room temperature (298 K) (Note: TC aqueous solution need avoid light). Then the concentration of micro-pollutant models in water is detected by an UV-visible spectrophotometer at the detection wavelength (MB: 664 nm and TC: 355 nm). All reported experimental data are the average values of three parallel experiments.

40 mg adsorbent is added to a 50 mL micro-pollutant solution (200 ppm for MB and 50 ppm for TC) and stirred immediately. At certain intervals about 1 mL of the suspension is withdrawn and filtered immediately through a Whatman 0.45 μm inorganic membrane to obtain the solution. After the filtration process, the residual concentration of the pollutant is analyzed by an UV-visible spectrophotometer. All samples need to be ground before testing.

Based on the changes in the initial and residual concentrations of micro-pollutants in the adsorption process, the adsorption capacity of the adsorbent at a specific time and at adsorption equilibrium can be determined through equations 1.1 and 1.2.

$$q_t = \frac{(c_0 - c_t)}{m} \times V \tag{1.1}$$

$$q_e = \frac{(c_0 - c_e)}{m} \times V \quad (1.2)$$

where q_t (mg g^{-1}) and q_e (mg g^{-1}) are the adsorption amounts of pollutant at the specific time t (min) and at the equilibrium, c_t (mg L^{-1}) is the concentration of the pollutant at a specific time, c_0 (mg L^{-1}) and c_e (mg L^{-1}) are the initial and equilibrium concentrations of the pollutants in the filtrate, respectively, V is the volume of the adsorption system (L), m is the mass of the adsorbent (g).

Adsorption efficiency:

The micro-pollutants removal efficiency is calculated by following equation:

$$\text{Removal efficiency (\%)} = \frac{(c_0 - c_t)}{c_0} \times 100 \quad (1.3)$$

where c_0 (mg L^{-1}) and c_t (mg L^{-1}) are the initial and residual concentrations of the pollutants in the filtrate at certain time, respectively.

Adsorption rate:

The adsorption rate is approximately described by linear the pseudo-first-order and pseudo-second-order adsorption models. The pseudo-first-order and pseudo-second-order adsorption models are represented by equations 1.4 and 1.5, respectively.

$$\log(q_e - q_t) = \log q_e - \frac{k_1}{2.303} t \quad (1.4)$$

$$\frac{t}{q_t} = \frac{1}{k_2 {q_e}^2} + \frac{1}{q_e} t \quad (1.5)$$

where q_t and q_e are the adsorption amounts of pollutants (mg g^{-1}) at the specific time t (min) and at equilibrium respectively, k_1 and k_2 are the pseudo-first-order adsorption rate constant (min^{-1}) and pseudo-second-order adsorption rate constant (g mg^{-1} min^{-1}).

Adsorption Isotherms:

3 mg of adsorbent is added into 18 mL different concentrations of micro-pollutants solutions, and the suspension is stirred at room temperature for 2 h to reach the adsorption equilibrium. After adsorption, 1 mL of each suspension is filtered through Whatman 0.45 μm inorganic membrane, and the resulting filtrated solution is subjected to UV-visible absorption spectroscopy to measure the remaining concentration (0-500 ppm for MB and 0-400 ppm for TC).

The Langmuir and Freundlich isothermal model are commonly used to study the adsorption behavior of adsorbents on pollutants and the maximum adsorption amount. The Langmuir and Freundlich isothermal model are represented by equations 1.6 and 1.7, respectively.

$$\frac{c_e}{q_e} = \frac{1}{q_{max} \times k_L} + \frac{c_e}{q_{max}} \tag{1.6}$$

$$\ln q_e = \ln k_F + \frac{1}{n} \ln c_e \tag{1.7}$$

where q_e is the equilibrium adsorption amount of pollutant (mg g^{-1}), q_{max} is the maximum adsorption capacity of adsorbent at adsorption equilibrium (mg g^{-1}), c_e is the remaining pollutant concentration after the adsorption equilibrium (mg L^{-1}), k_L is the Langmuir isothermal adsorption equilibrium constant (L mg^{-1}), k_F and n are associated with the uptake coefficients of Freundlich.

Model Equation:

The Weber-Morris intraparticle diffusion (IPD) model is a widely used kinetic model to evaluate the contribution of intraparticle diffusion to the adsorption process. It is mainly applied to analyze whether the adsorption rate is controlled by intraparticle diffusion, and to further explore the adsorption mechanism. The linear form of the Weber–Morris intraparticle diffusion model is expressed as follows:

$$q_t = K_{id} t^{1/2} + C \tag{1.8}$$

Where q_t (mg g^{-1}) is the amount of adsorbate adsorbed per unit mass of adsorbent at any time. K_{id} (mg g^{-1} $min^{-1/2}$) is the rate constant for intra-particle diffusion, which reflects the rate of intra-particle diffusion during the adsorption process; a higher value indicates a faster rate of intra-particle diffusion. t (min) represents the adsorption time. C (mg g^{-1}) is an experiment-dependent constant that is positively correlated with the thickness of the boundary layer surrounding the adsorbent.

Gibbs Free Energy:

The Gibbs free energy (ΔG^0) is calculated using the following formula:

$$\Delta G = -RT \ln(K_L) \tag{1.9}$$

where T (in kelvins, 25 °C) is the solution temperature, R (8.314 J mol^{-1} K^{-1}) is the ideal gas constant, and K_L (L g^{-1}) is the adsorption equilibrium constant obtained by fitting the Langmuir isotherm model. K_L should be unitless to get the correct units, the Langmuir isotherm coefficient K_L was converted into a dimensionless constant by multiplying it by 1000.

Activation Energy:

The activation energy (E_a) is calculated using the linear form of the Arrhenius equation:

$$\ln k = -\frac{E_a}{RT} + \ln A \tag{1.10}$$

where E_a (J·mol^{-1}) is the apparent activation energy of the reaction; R (8.314 J mol^{-1} K^{-1}) is the ideal

gas constant; T (K) is the reaction temperature and k (g mg^{-1} min^{-1}) is the second-order rate constant. Since the adsorption process follows a pseudo-second-order kinetic model, the apparent activation energy is calculated using the rate constant k_2 of this model. Plotting a graph with 1/T on the x-axis and ln k on the y-axis, E_a can be determined from the slope of the curve.

Effect of pH and temperature:

5 mg PAF-525-9 is added to a fixed concentration of micro-pollutant solution at different pH values and temperatures for 2 h to reach adsorption equilibrium. After adsorption, 1 mL of each suspension is filtered through Whatman 0.45 μm inorganic membrane, and the resulting filtrated solution is subjected to UV-visible absorption spectroscopy to measure the concentration (where pH = 2, 3, 4, 5, 6 and 7, T = 25 °C, 35 °C and 45 °C). The pH values of micro-pollutant solutions are adjusted by adding 1M HCl or NaOH solutions.

Adsorption cyclicity:

200 mg PAF-525-9 is added into 250 mL micro-pollutant solutions (200 ppm for MB and 50 ppm for TC) under stirring for 1 h. And then the suspension is filtered. PAF-525-9 is collected by vacuum filtration. PAF-525-9 is stirred in a 200 mL of 0.1 mol L^{-1} HCl methanol solution at overnight for desorption, filtered to obtain the adsorbent and then dried under vacuum at 60 °C. The adsorption-desorption process is repeated five times.

Synthesis of PAF-525-9-X

The PAF-525-9-X samples were synthesized following the same synthetic procedure of PAF-525-9, with the dosage of chlorosulfonic acid being the sole modified parameter. Specifically, the dosage of chlorosulfonic acid used for synthesizing PAF-525-9-50% was 50% of that required for PAF-525-9, while the corresponding dosage for PAF-525-9-25% was reduced to 25% of the amount adopted for the PAF-525-9.

Synthesis of meso-PAM-15-1 Precursors

0.2 g of phenyl-functionalized SiO_2 was ultrasonically dispersed in 0.7 mL of toluene and 0.7 mL porogen (toluene), followed by the addition of 0.21 g Span 80, and the mixture was stirred vigorously for 2 h under continuous mechanical stirring (300 rpm) to form a homogeneous oil phase. Subsequently, water phase containing 0.013 g $K_2S_2O_8$, 0.067 g $CaCl_2$ and 15 mL H_2O is added dropwise at a rate of 1.5 mL min^{-1}. After finishing dropwise addition of water phase, the high internal phase emulsion is polymerized at 60 °C for 48 h.

The samples are further extracted with ethanol using Soxhlet extraction for 2 d to remove the emulsifier. Subsequently, the SiO_2 spheres were etched using an ethanol solution of sodium hydroxide for 1 d, followed by Soxhlet extraction with ethanol for 1 d and freeze dried for 1 d to obtain meso-PAM-15-1.

Synthesis of meso-PAFs-525-4

1 g meso-PAM-15-1 and 50 mL DCE are added in 100 mL round bottom flask to thoroughly swell porous framework for 1 d. 10 mL $ClSO_3H$ is dropwise added into the reaction system under an ice water bath, followed by stirring at room temperature for 1 d to complete sulfonation. Finally, the obtained sample is quenched in ice water, washed by distilled water to neutral, extracted with ethanol for 2 d, and then dried under vacuum at 60 °C to obtain the meso-PAFs-525-4.

Theoretical Method:

The molecular geometries of PAF-525-9, MB, TC, as well as the MB/PAF-525-9 and TC/PAF-525-9 complexes, were optimized at the M06-2X/6-31G(d) level of theory[1]. Vibrational frequency calculations were further performed at the identical level to confirm that all optimized structures corresponded to true local minima without imaginary frequencies. All computational calculations were implemented using the Gaussian 16 program package[2]. The molecular electrostatic potential (ESP) distributions and intermolecular weak interactions were visualized via the Multiwfn program, employing the independent gradient model based on Hirshfeld partition (IGMH)[3-5].

Supporting Figures:

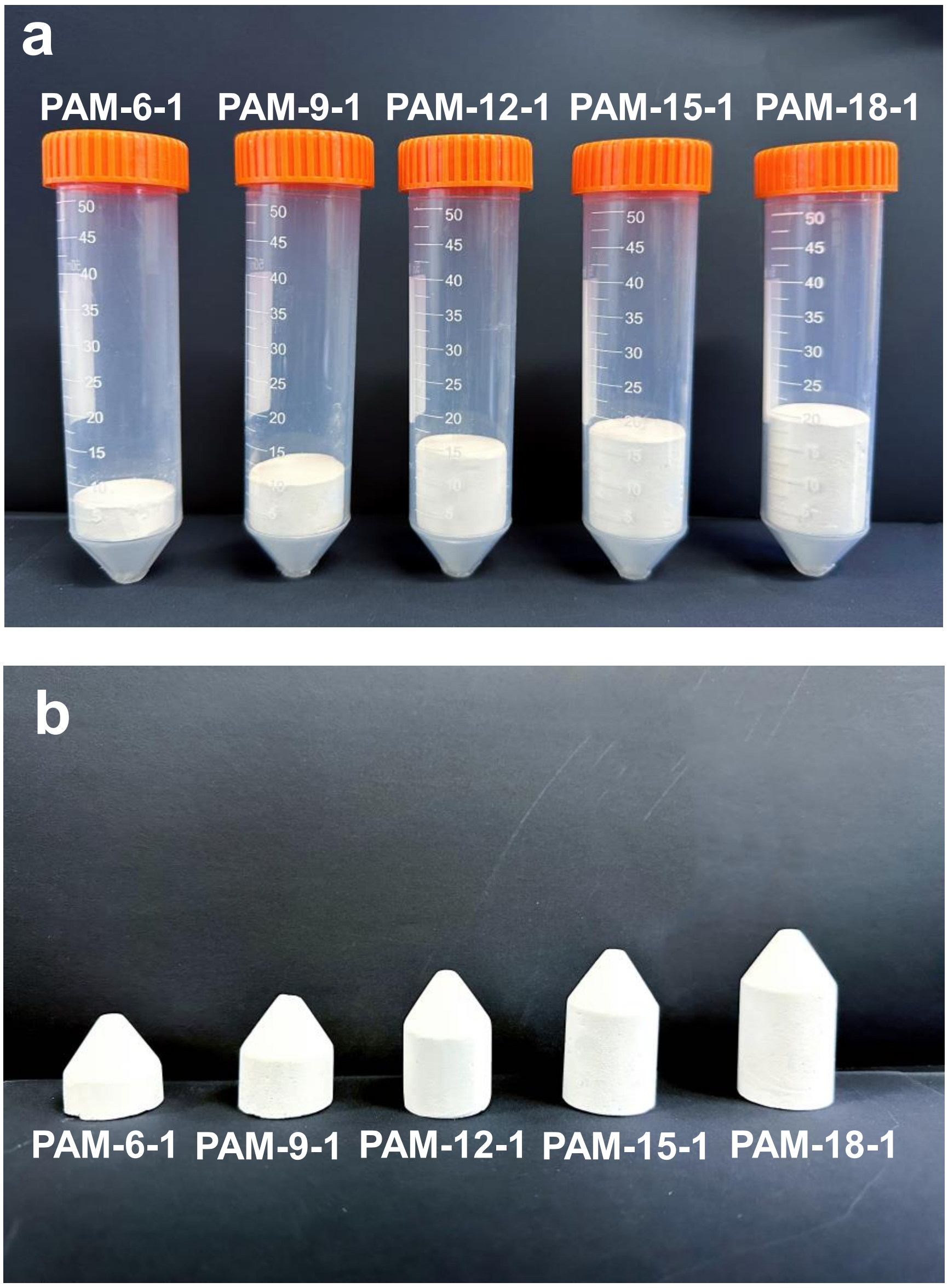

Figure S1. Optical photograph of PAM-6-1, PAM-9-1, PAM-12-1, PAM-15-1 and PAM-18-1.

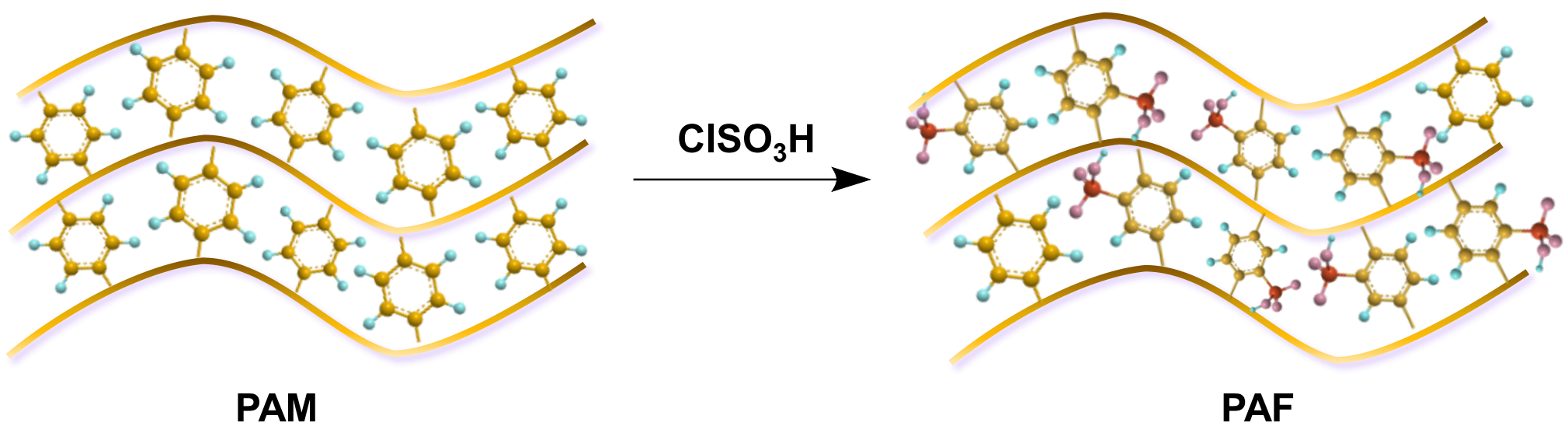

Figure S2. Chemical structure of PAM precursors and PAFs materials.

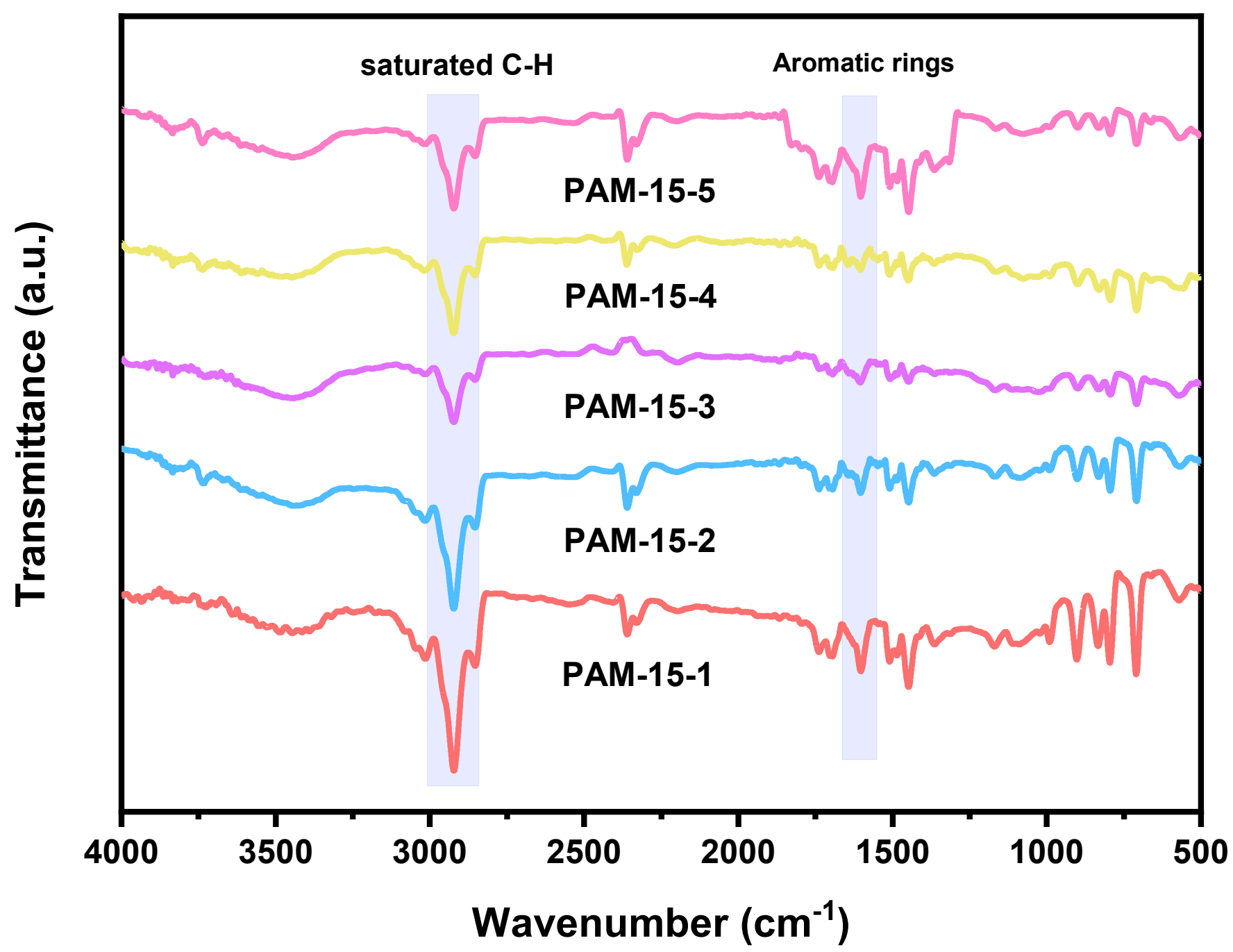

Figure S3. FT-IR spectra of PAM-15-1, PAM-15-2, PAM-15-3, PAM-15-4 and PAM-15-5.

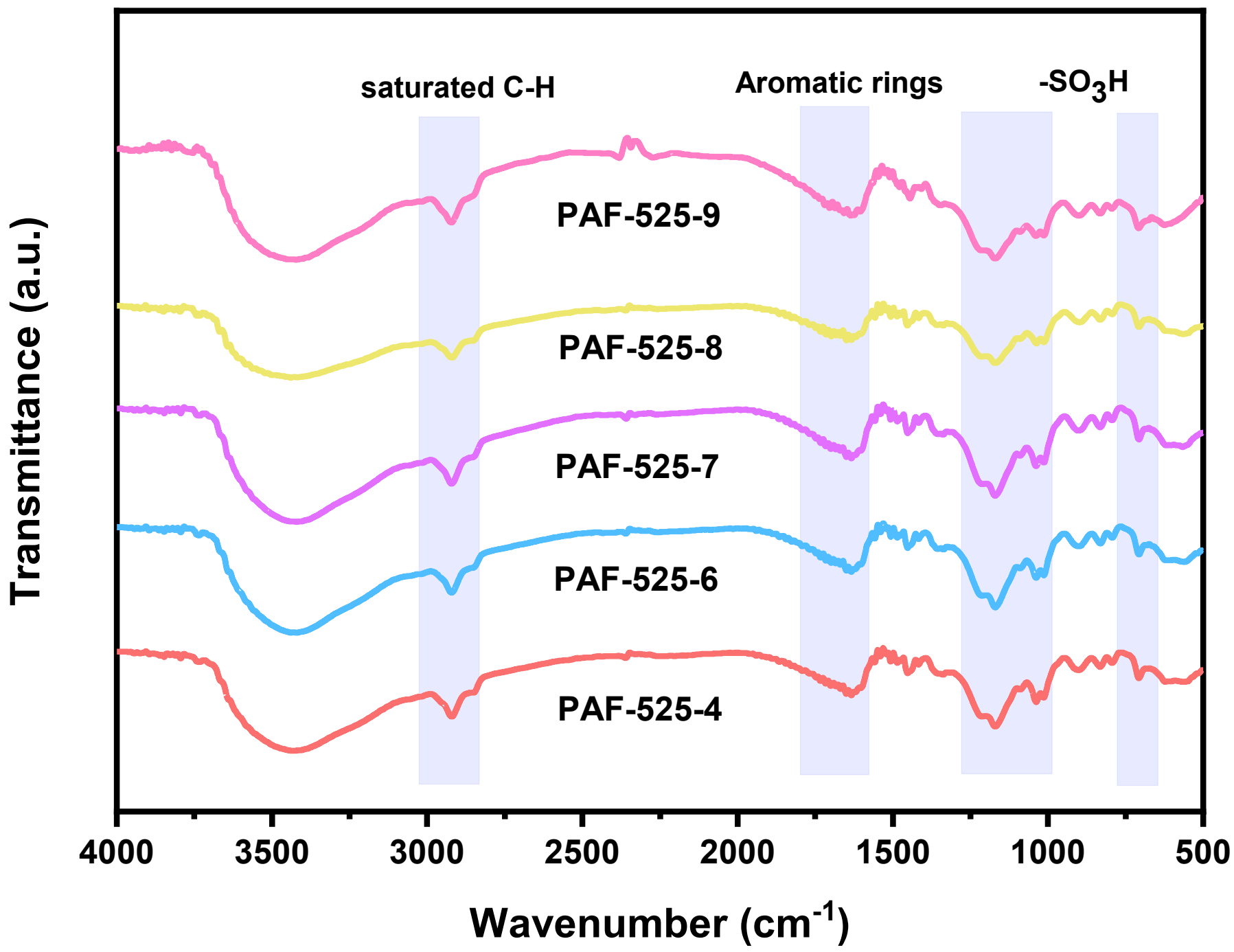

Figure S4. FT-IR spectra of PAF-525-4, PAF-525-6, PAF-525-7, PAF-525-8 and PAF-525-9.

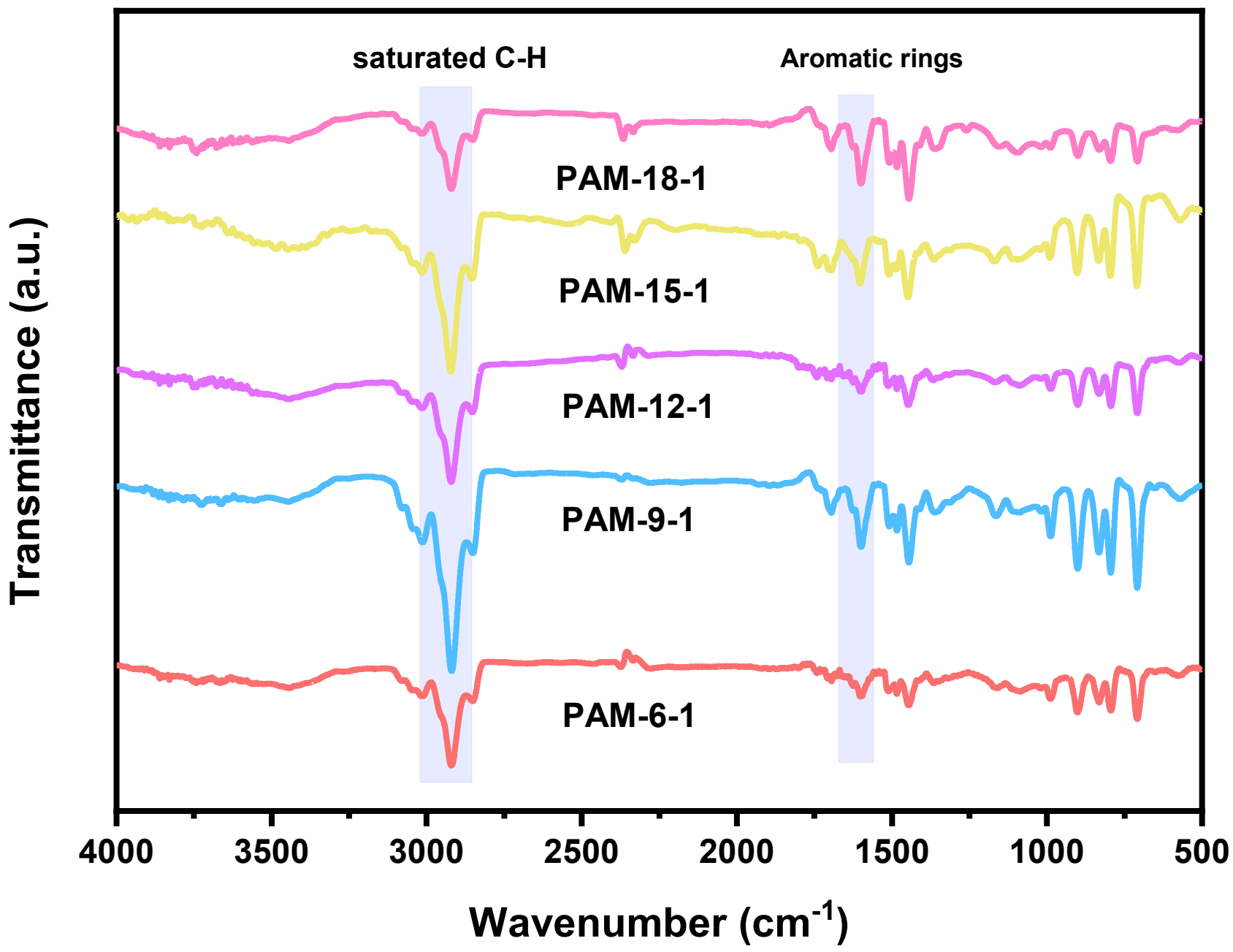

Figure S5. FT-IR spectra of PAM-6-1, PAM-9-1, PAM-12-1, PAM-15-1 and PAM-18-1.

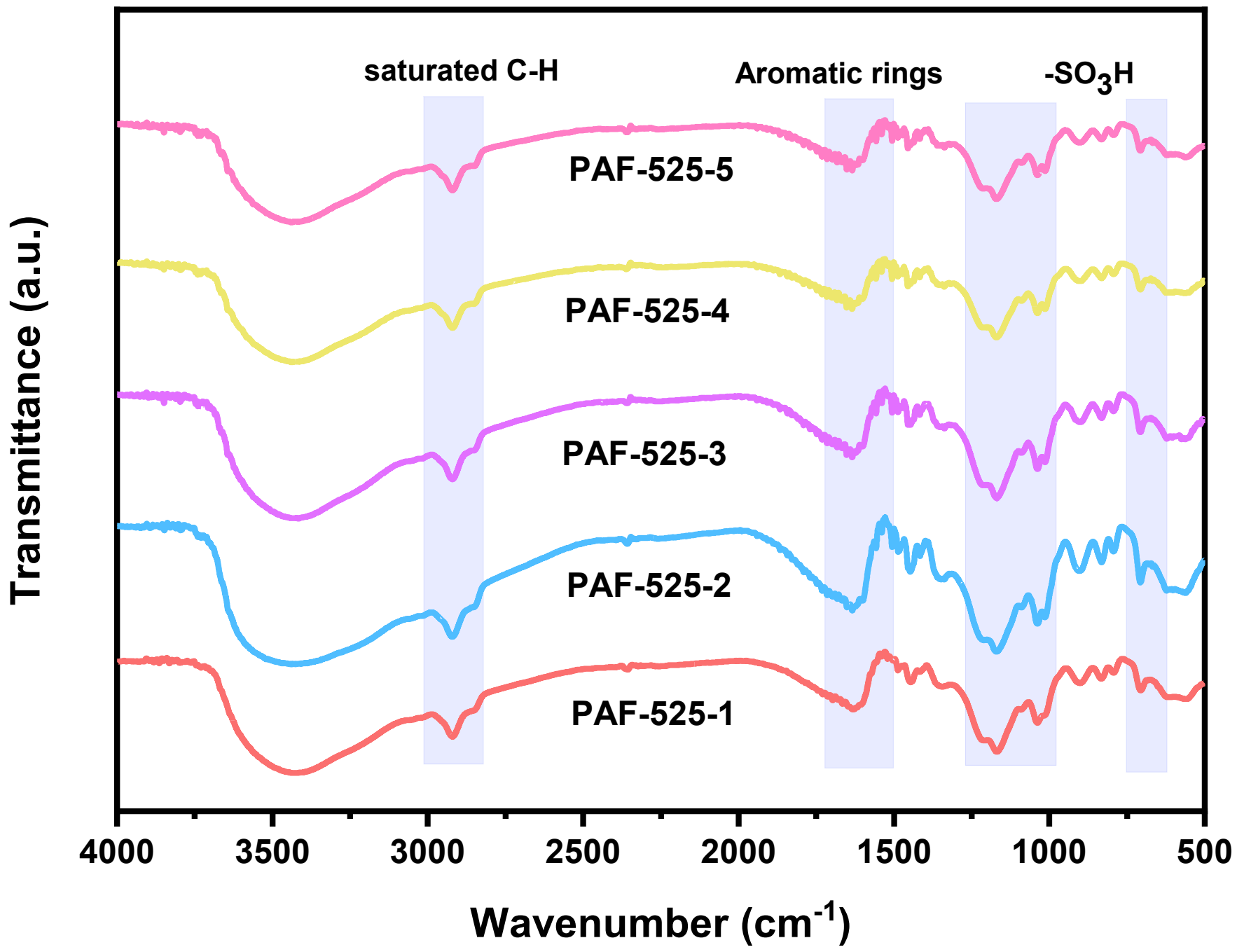

Figure S6. FT-IR spectra of PAF-525-1, PAF-525-2, PAF-525-3, PAF-525-4 and PAF-525-5.

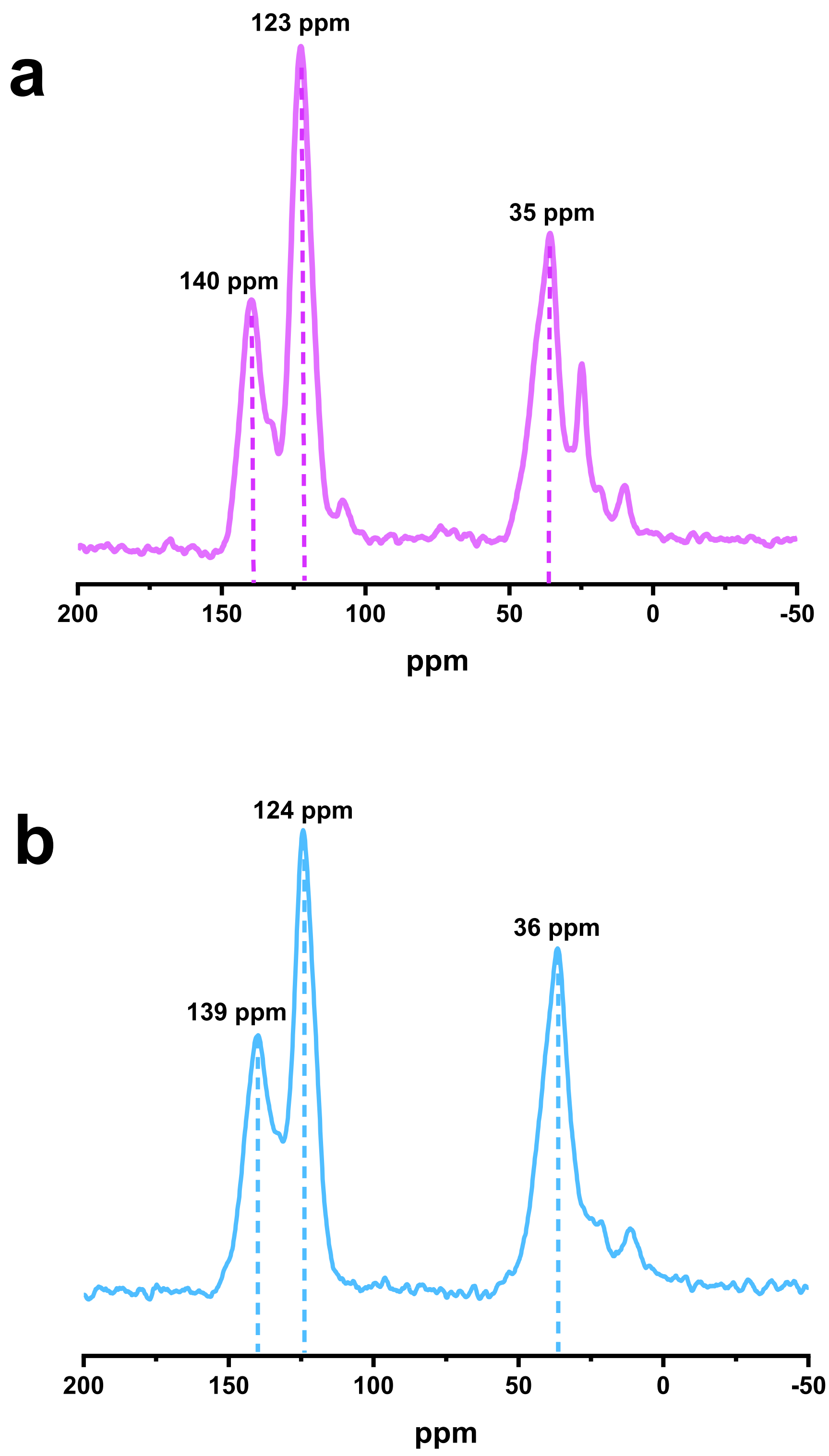

Figure S7. Solid-state ^{13}C CP-MAS NMR spectra of PAM-15-5 (a) and PAF-525-9 (b).

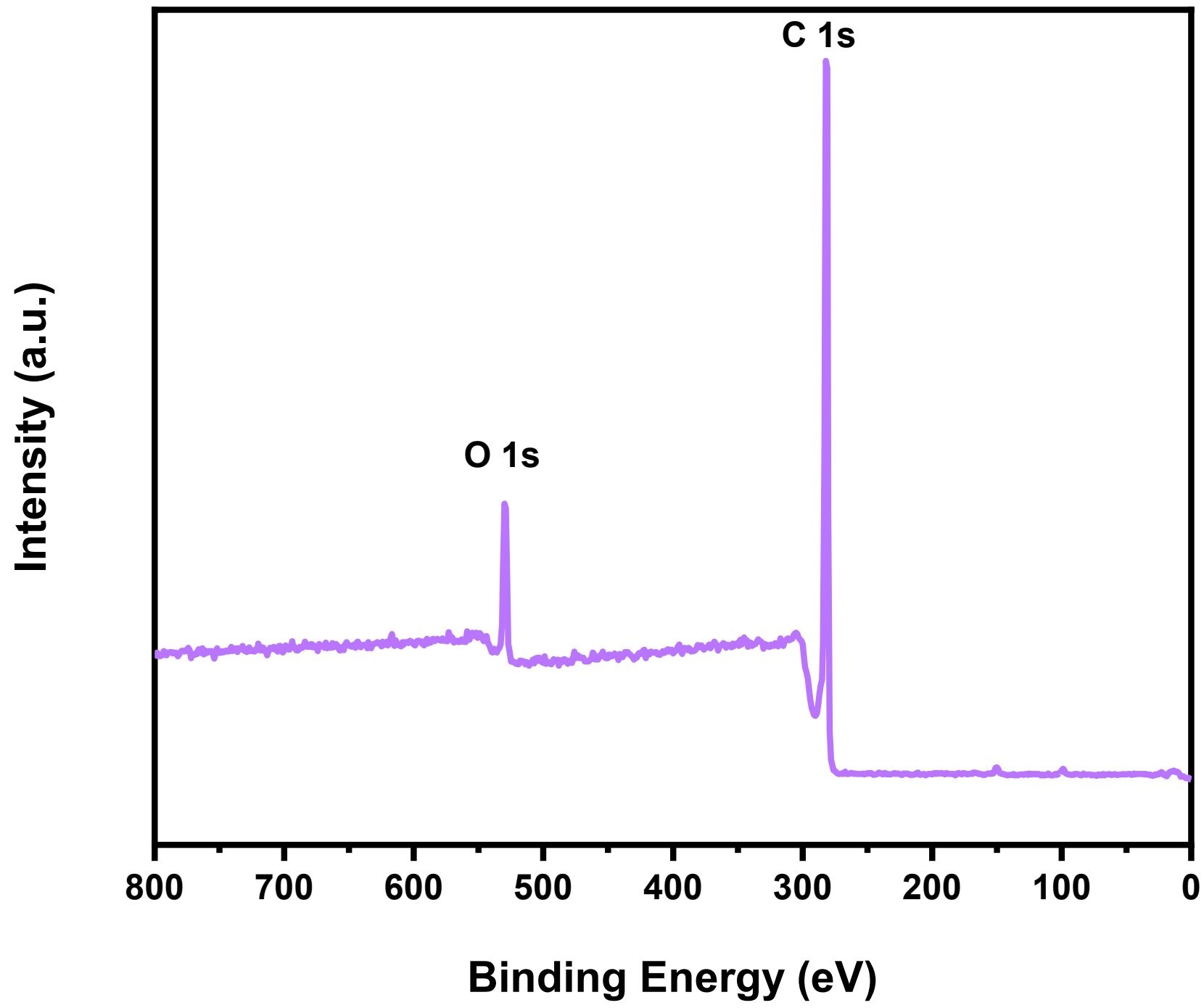

Figure S8. XPS spectrum of PAM-15-5.

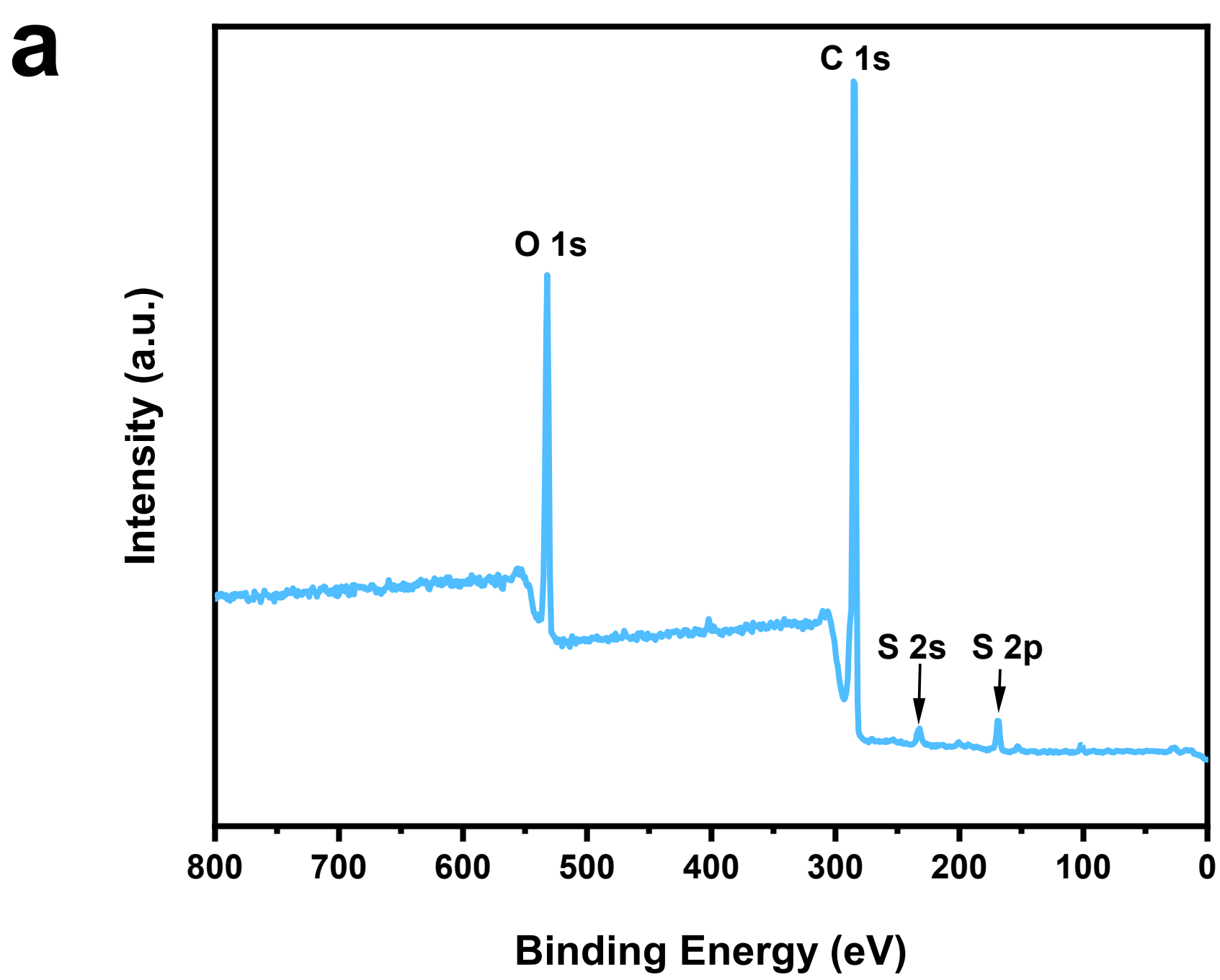

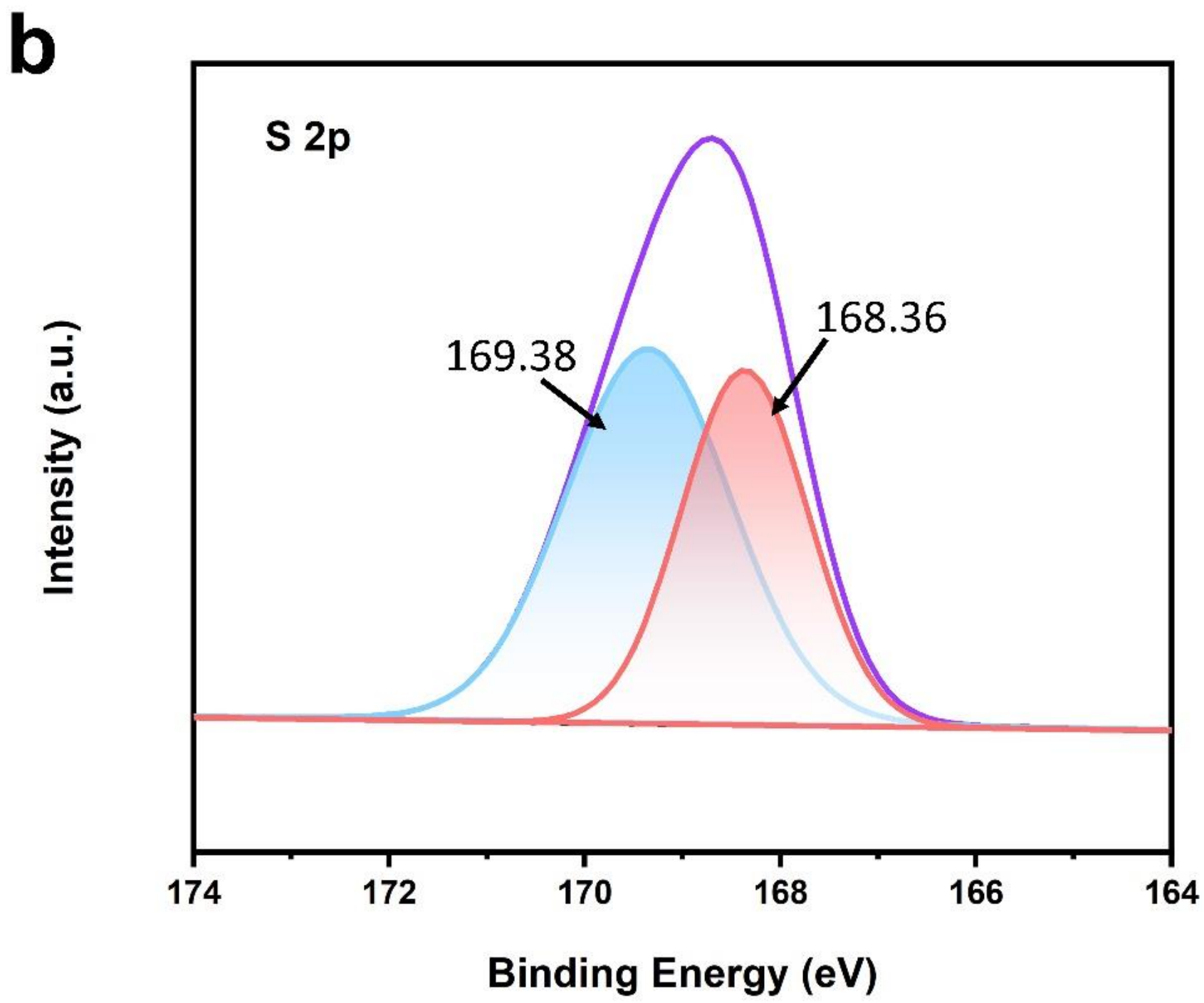

Figure S9. (a) XPS spectrum and (b) S 2p high resolution XPS spectra of PAF-525-9.

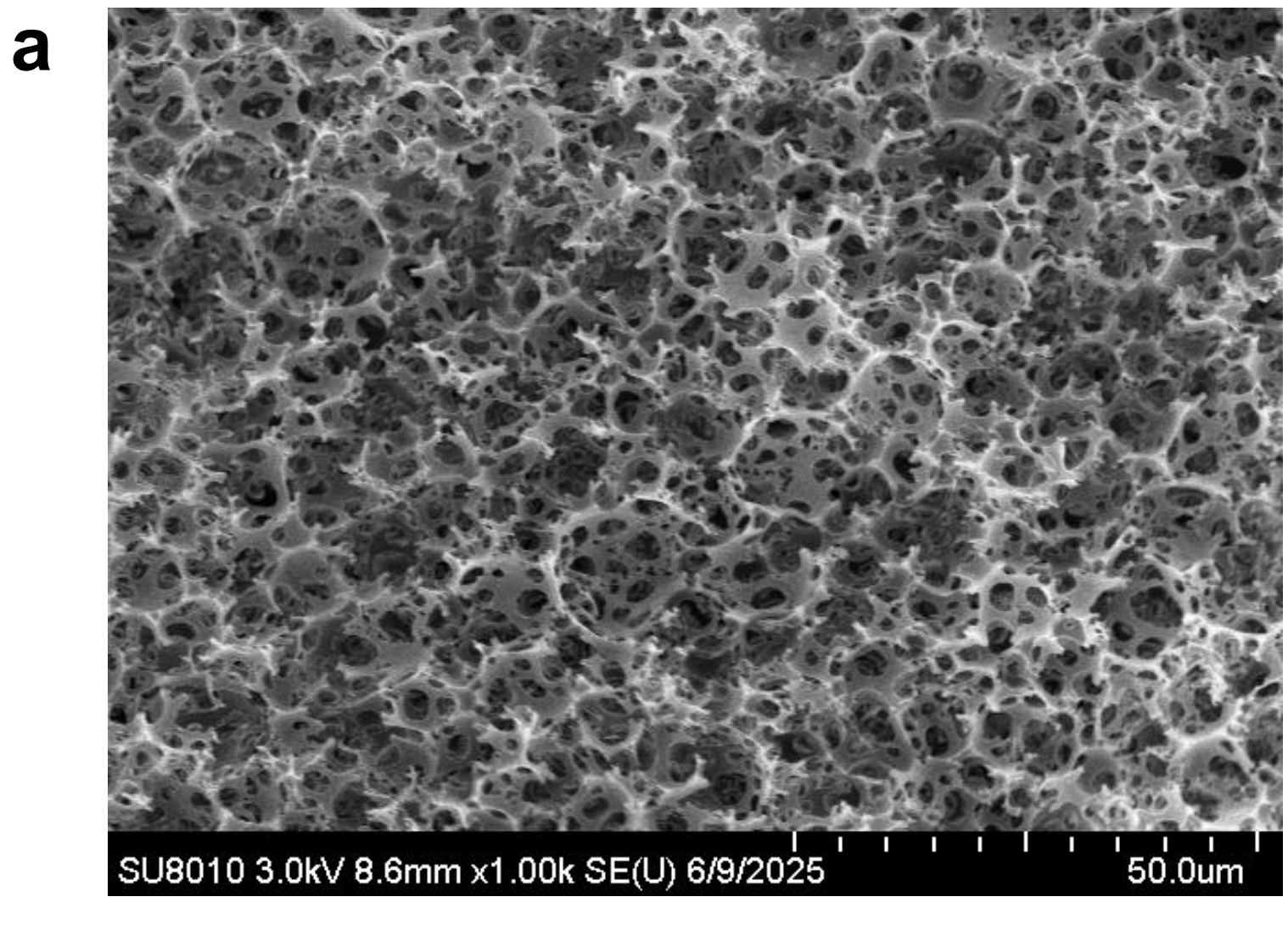

b

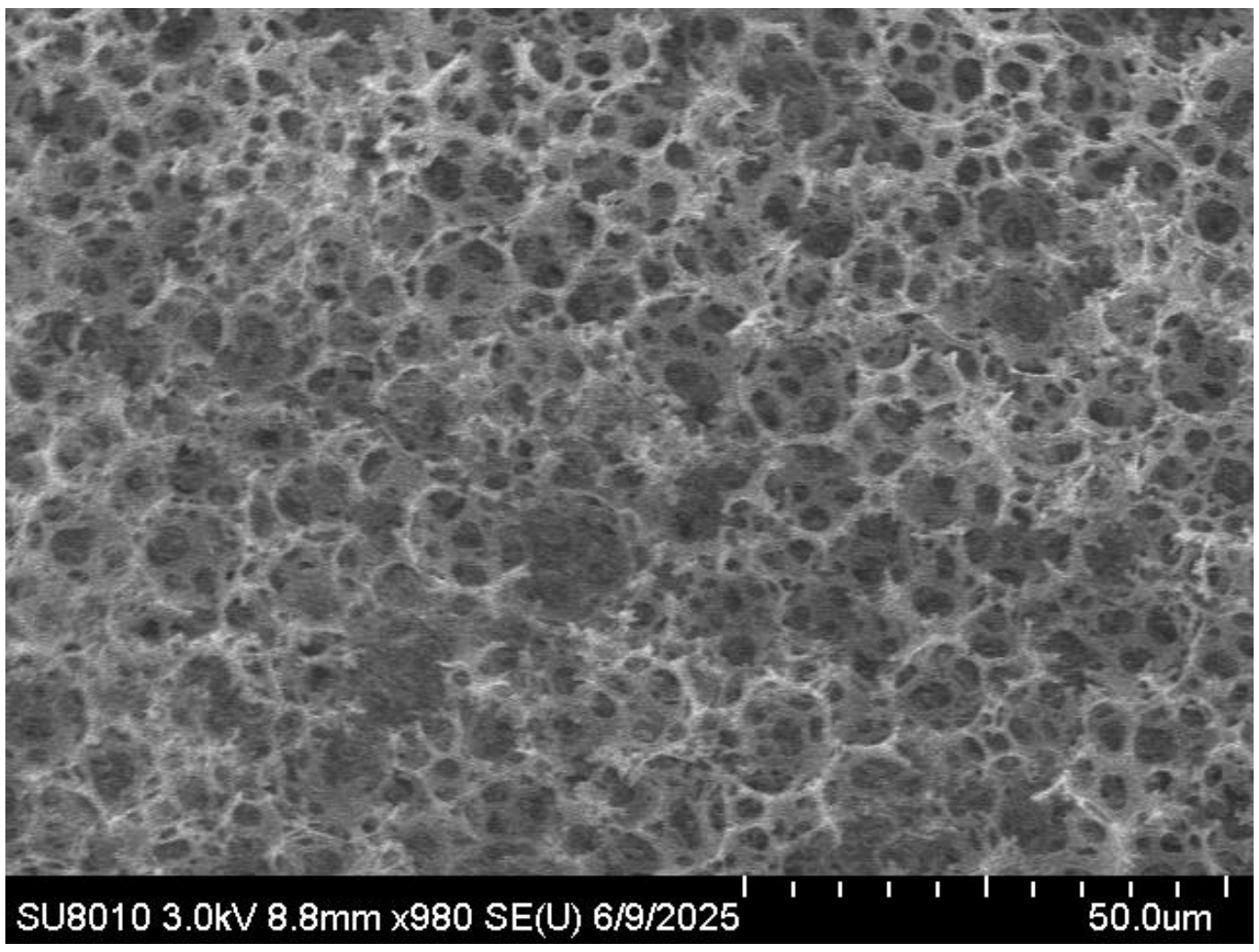
SU8010 3.0kV 8.8mm x980 SE(U) 6/9/2025
50.0um

c

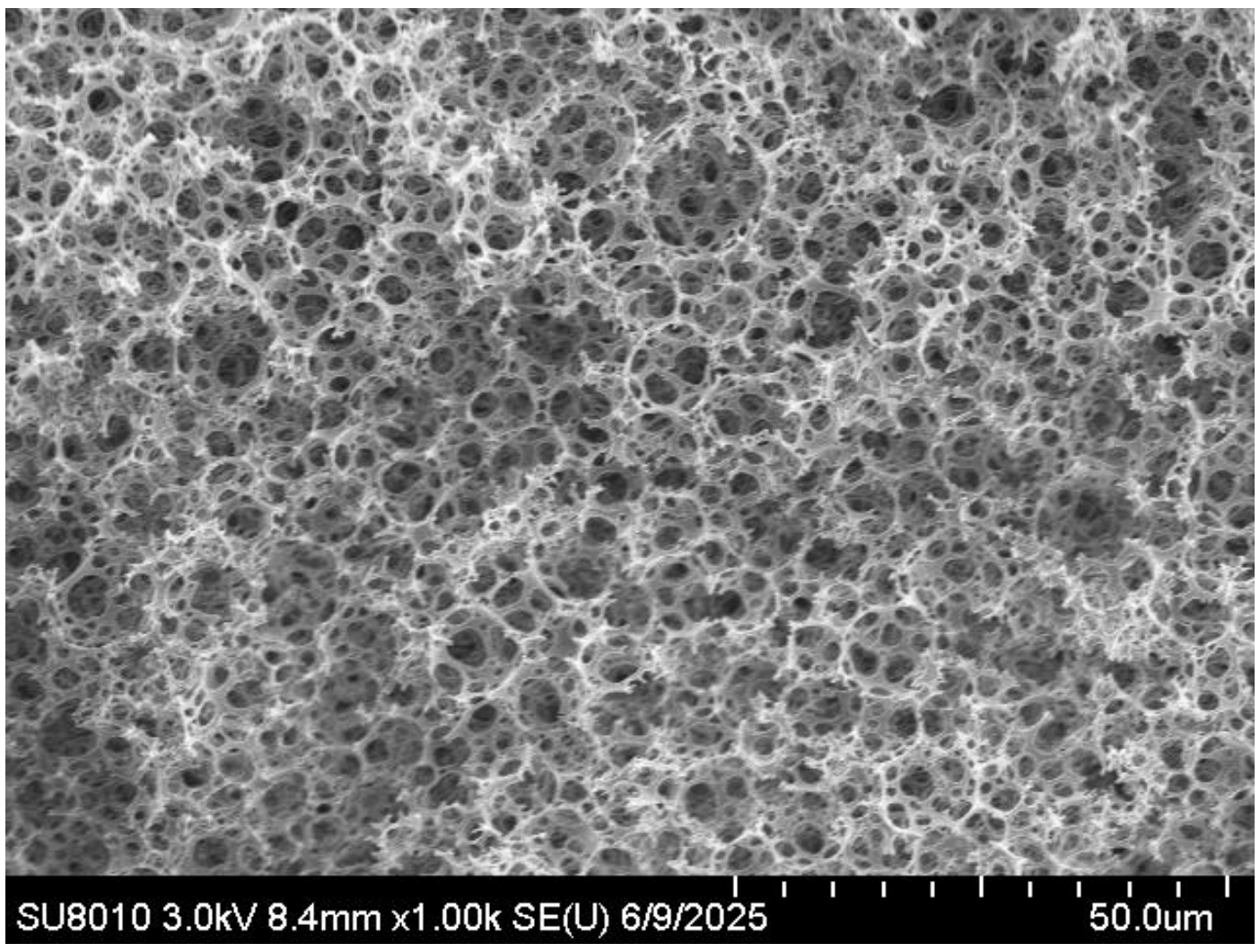
SU8010 3.0kV 8.4mm x1.00k SE(U) 6/9/2025
50.0um

d

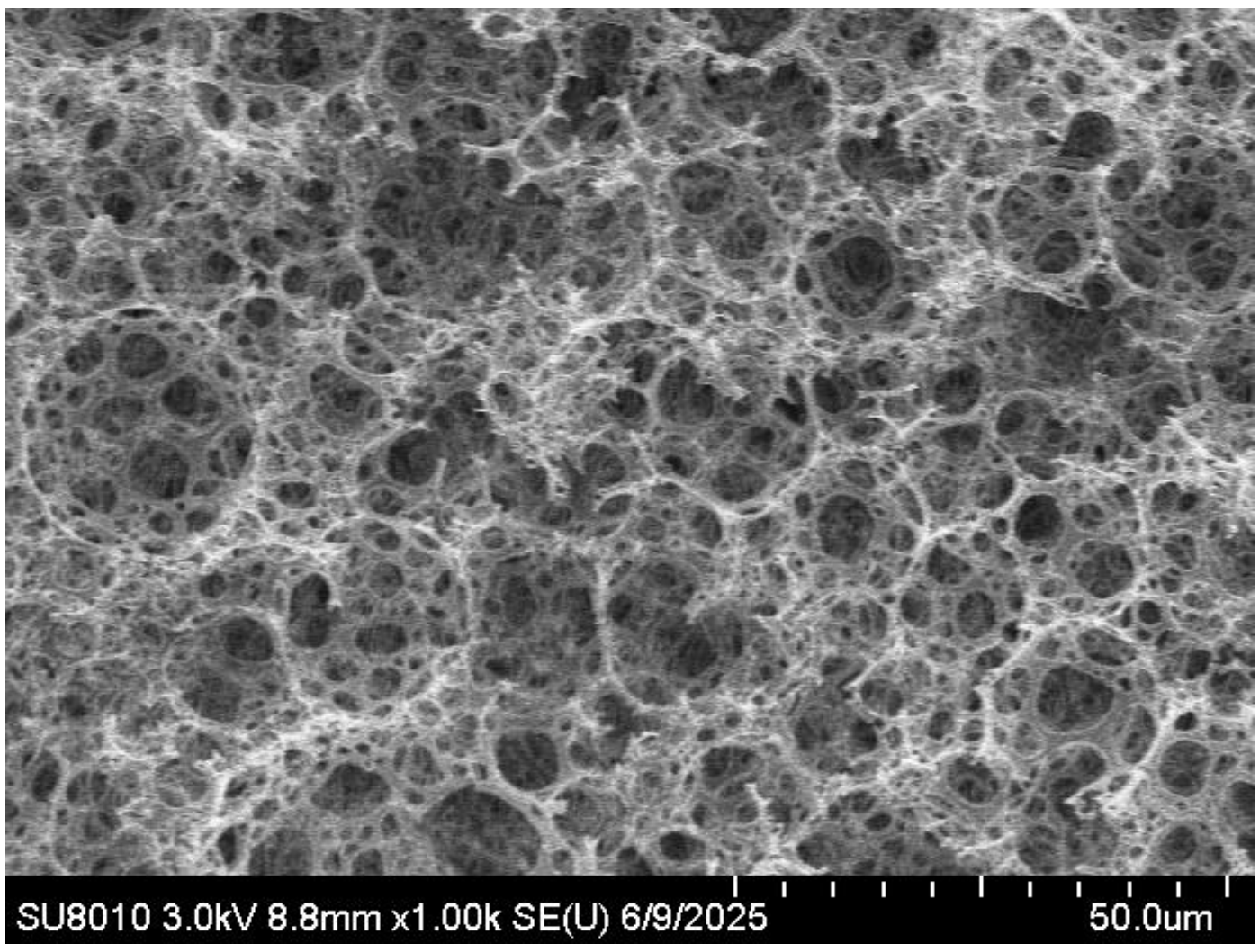
SU8010 3.0kV 8.8mm x1.00k SE(U) 6/9/2025
50.0um

e

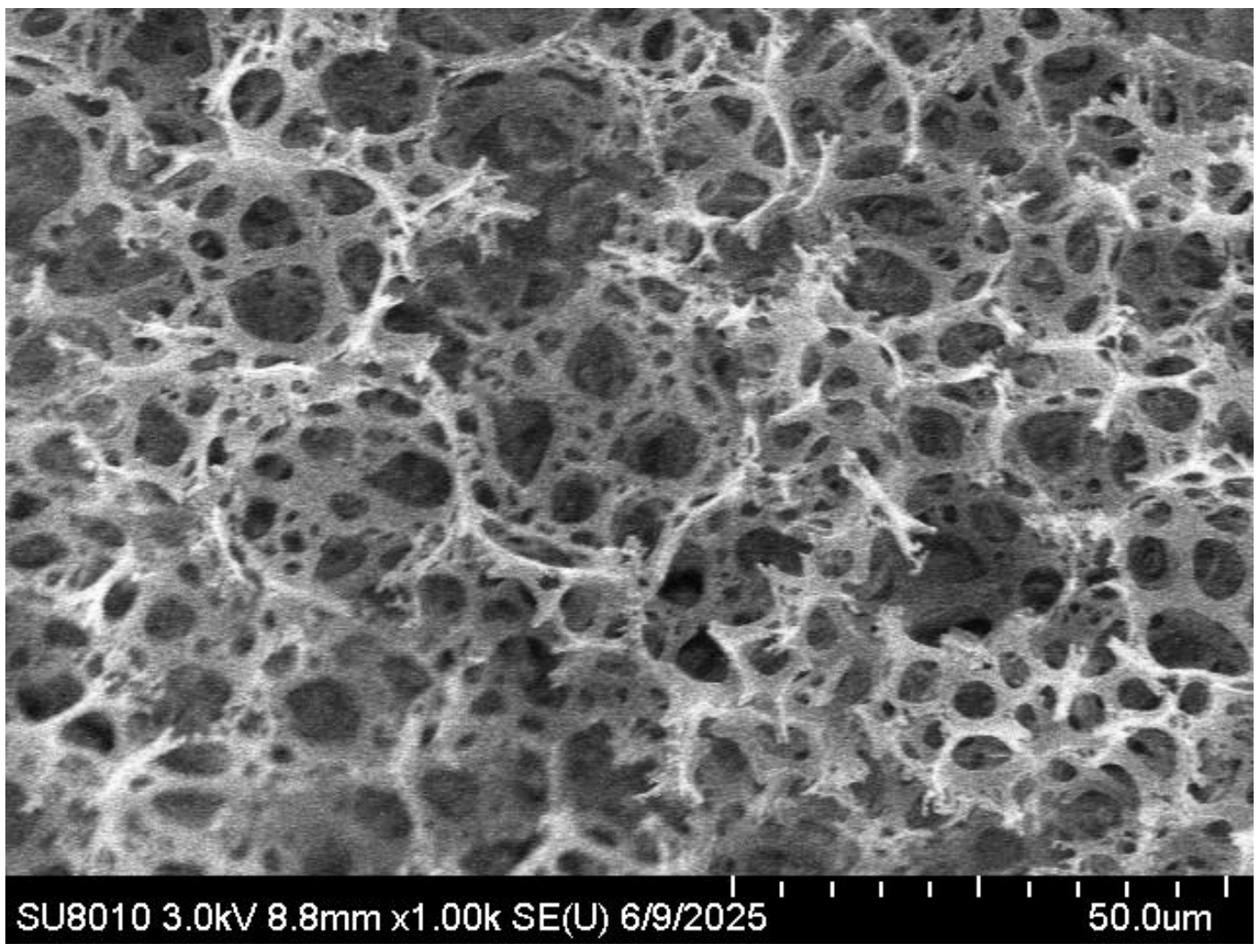

Figure S10. FE-SEM images of PAF-525-1 (a), PAF-525-2 (b), PAF-525-3 (c), PAF-525-4 (d) and PAF-525-5 (e) at 50 μm scale bars.

a

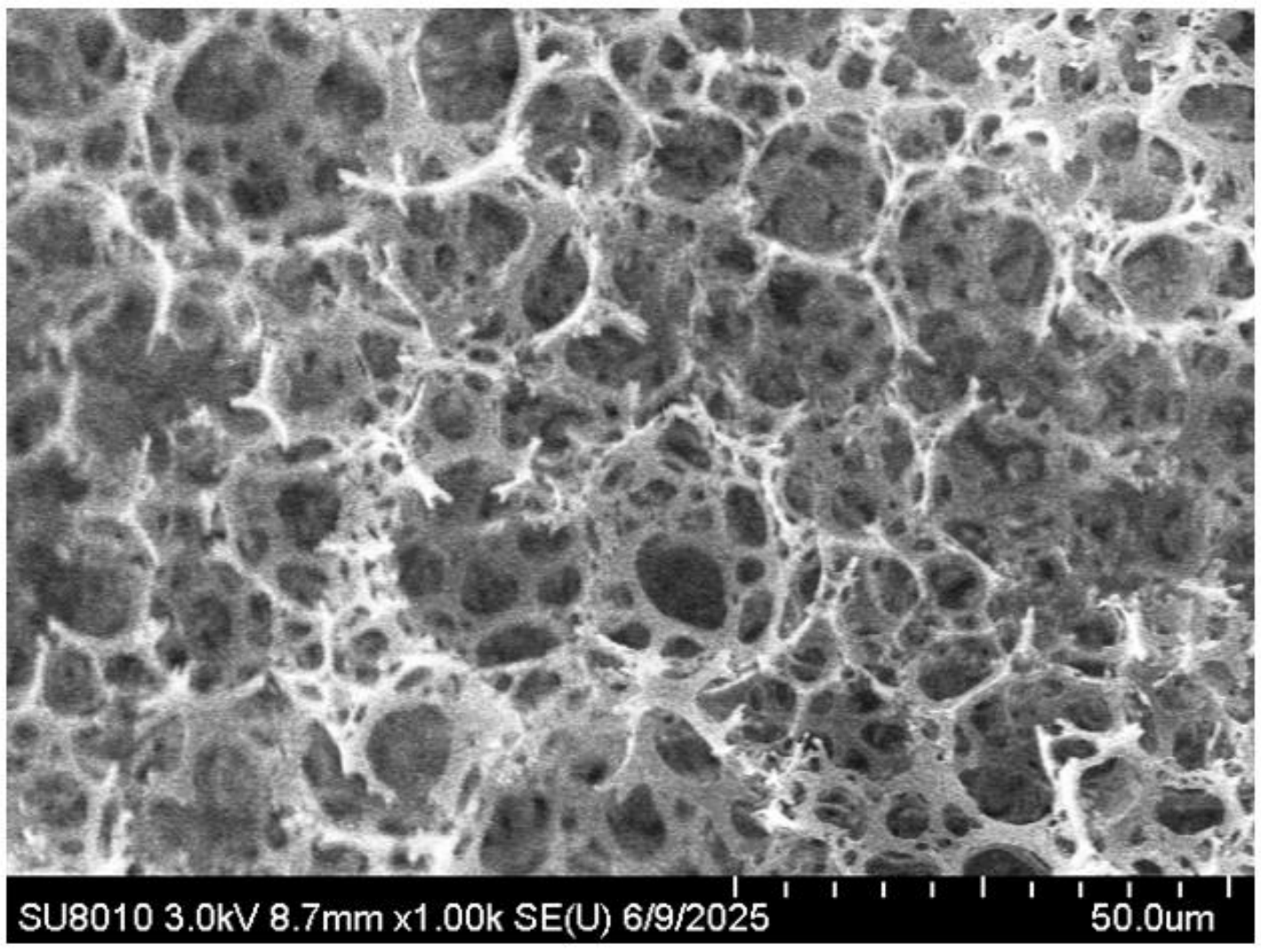

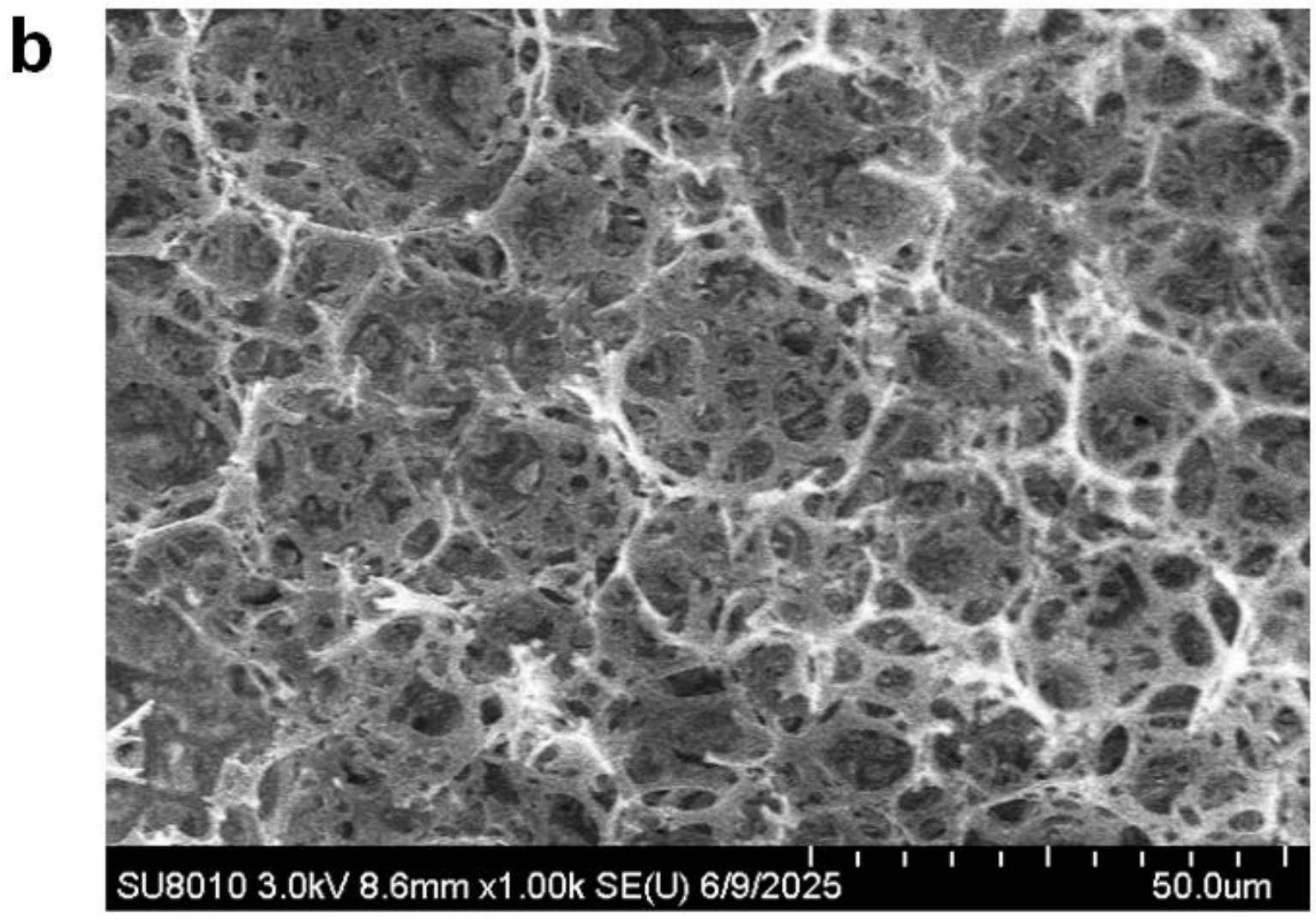

Figure S11. FE-SEM images of PAF-525-4 (a) and PAF-525-9 (b) at 50 μm scale bars.

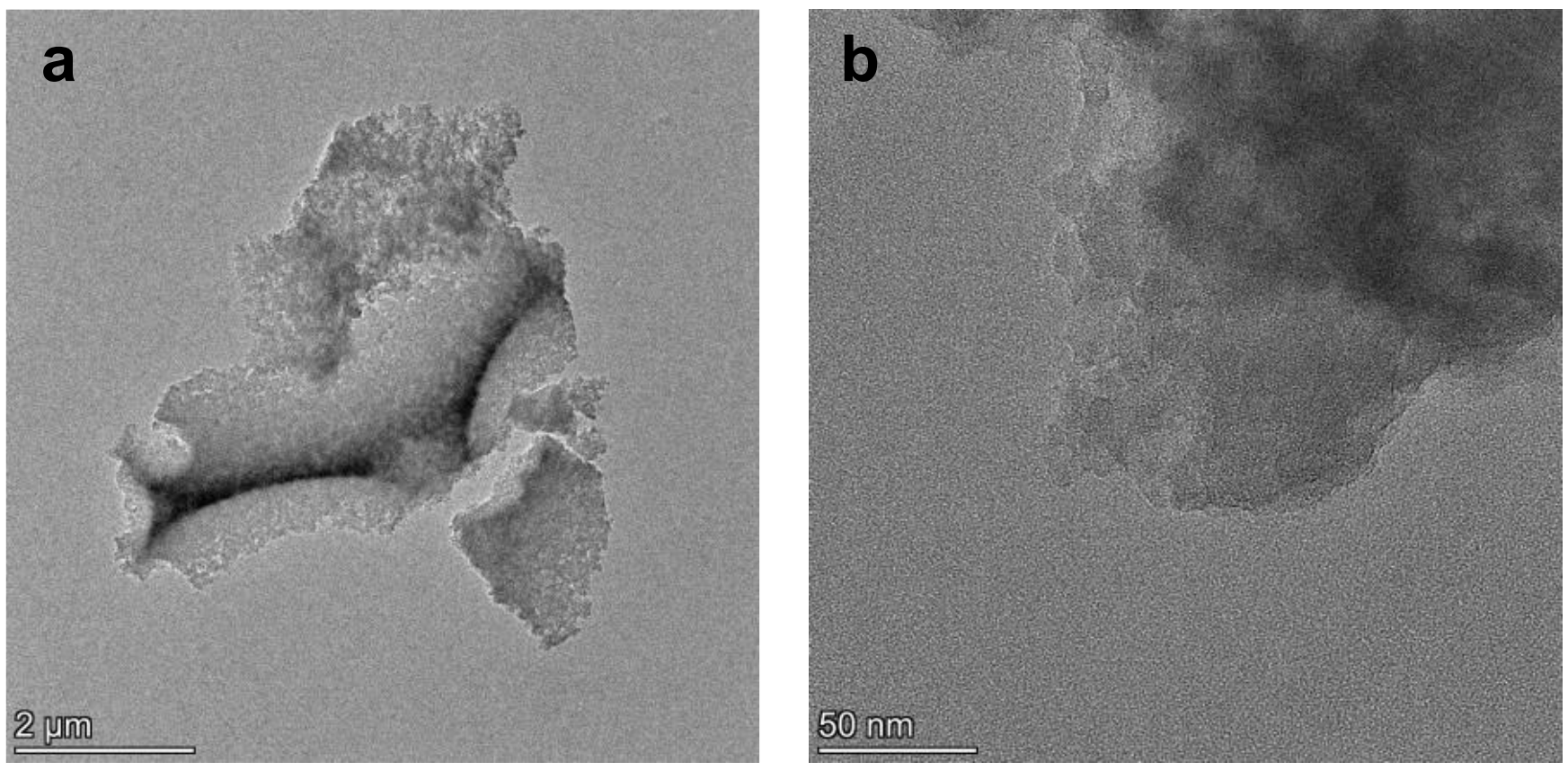

Figure S12. HR-TEM images of PAF-525-4 at 2 μm and 50 nm scale bars.

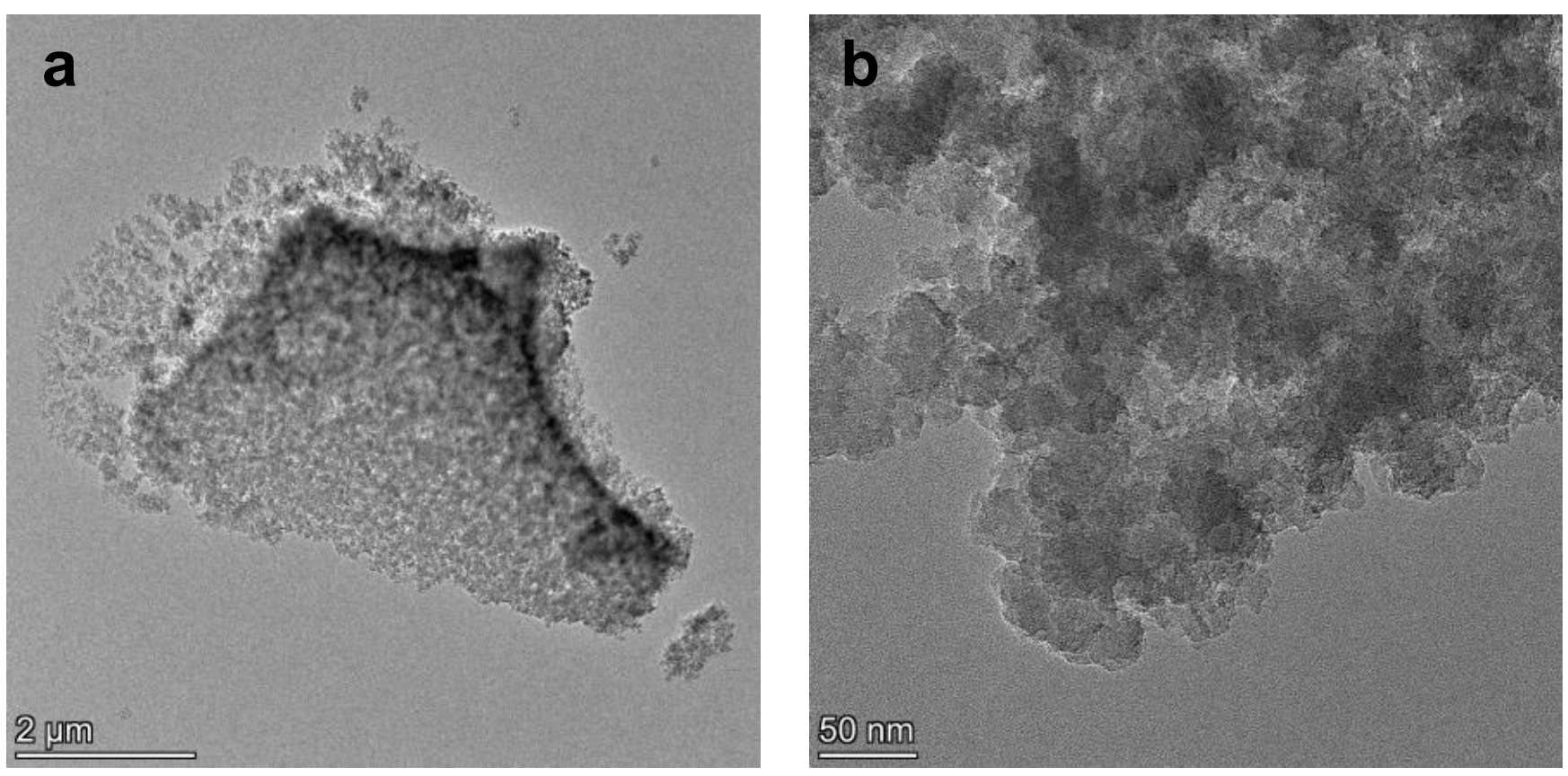

Figure S13. HR-TEM images of PAF-525-6 at 2 μm and 50 nm scale bars.

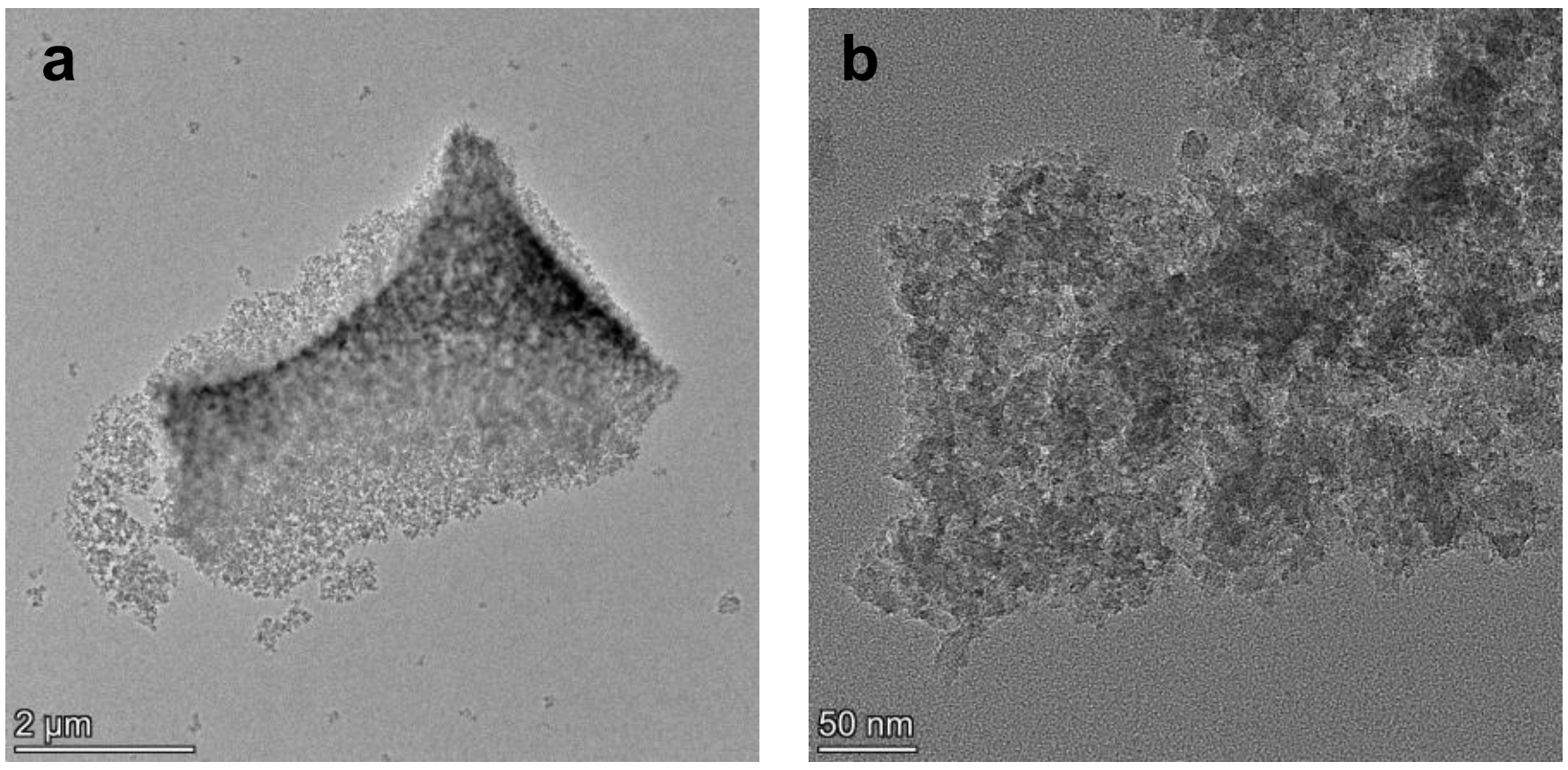

Figure S14. HR-TEM images of PAF-525-7 at 2 μm and 50 nm scale bars.

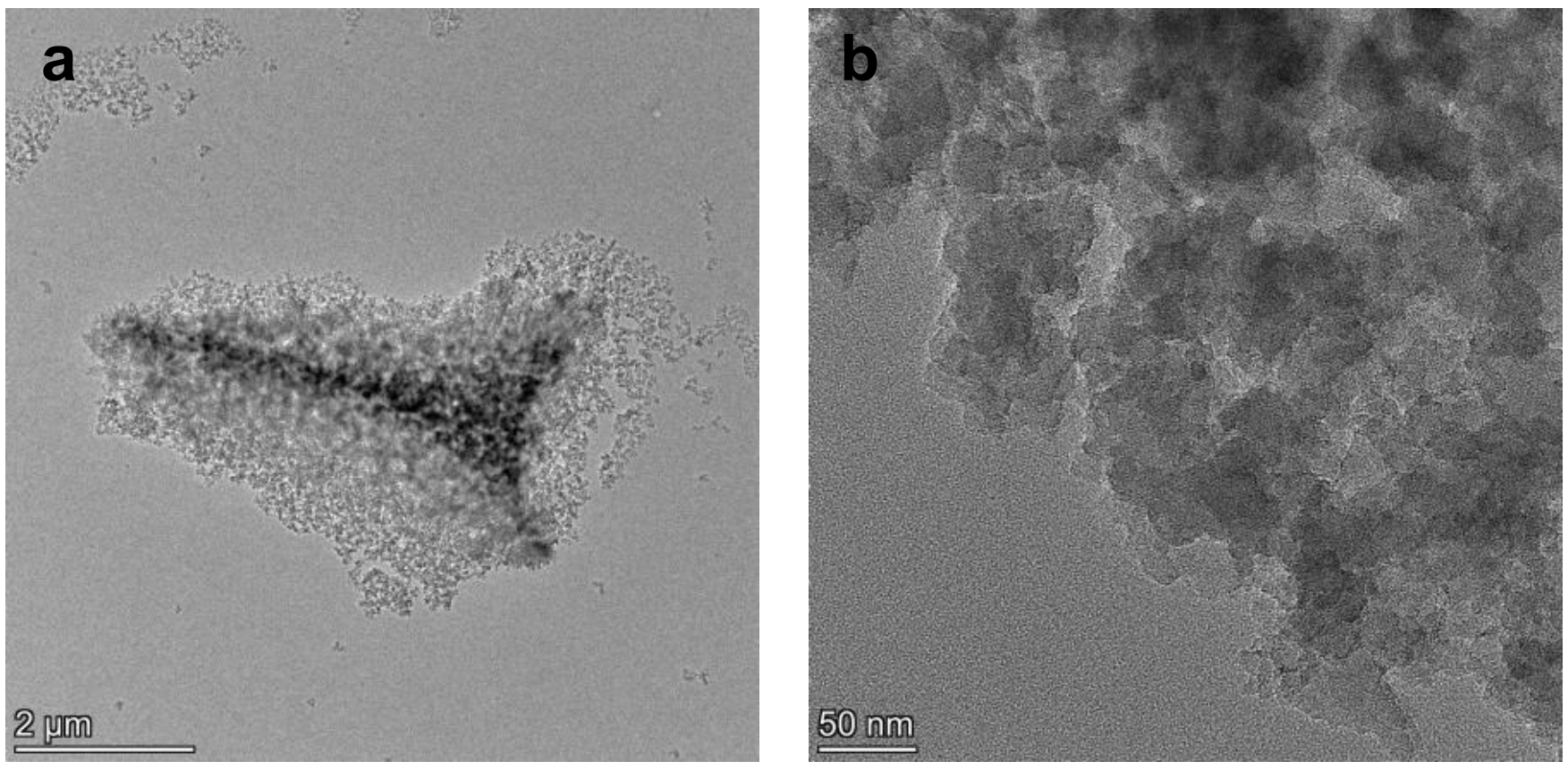

Figure S15. HR-TEM images of PAF-525-8 at 2 μm and 50 nm scale bars.

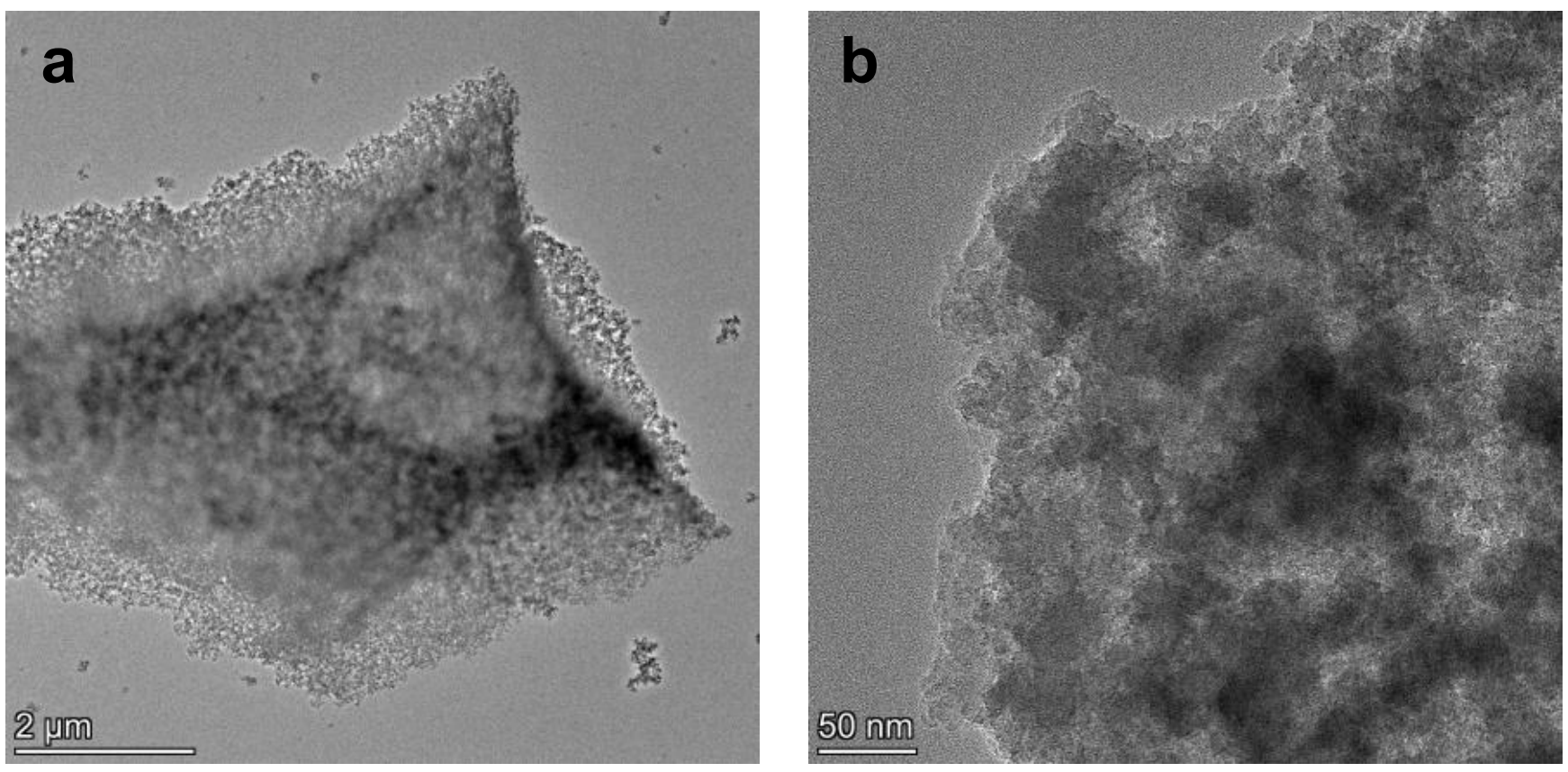

Figure S16. HR-TEM images of PAF-525-9 at 2 μm and 50 nm scale bars.

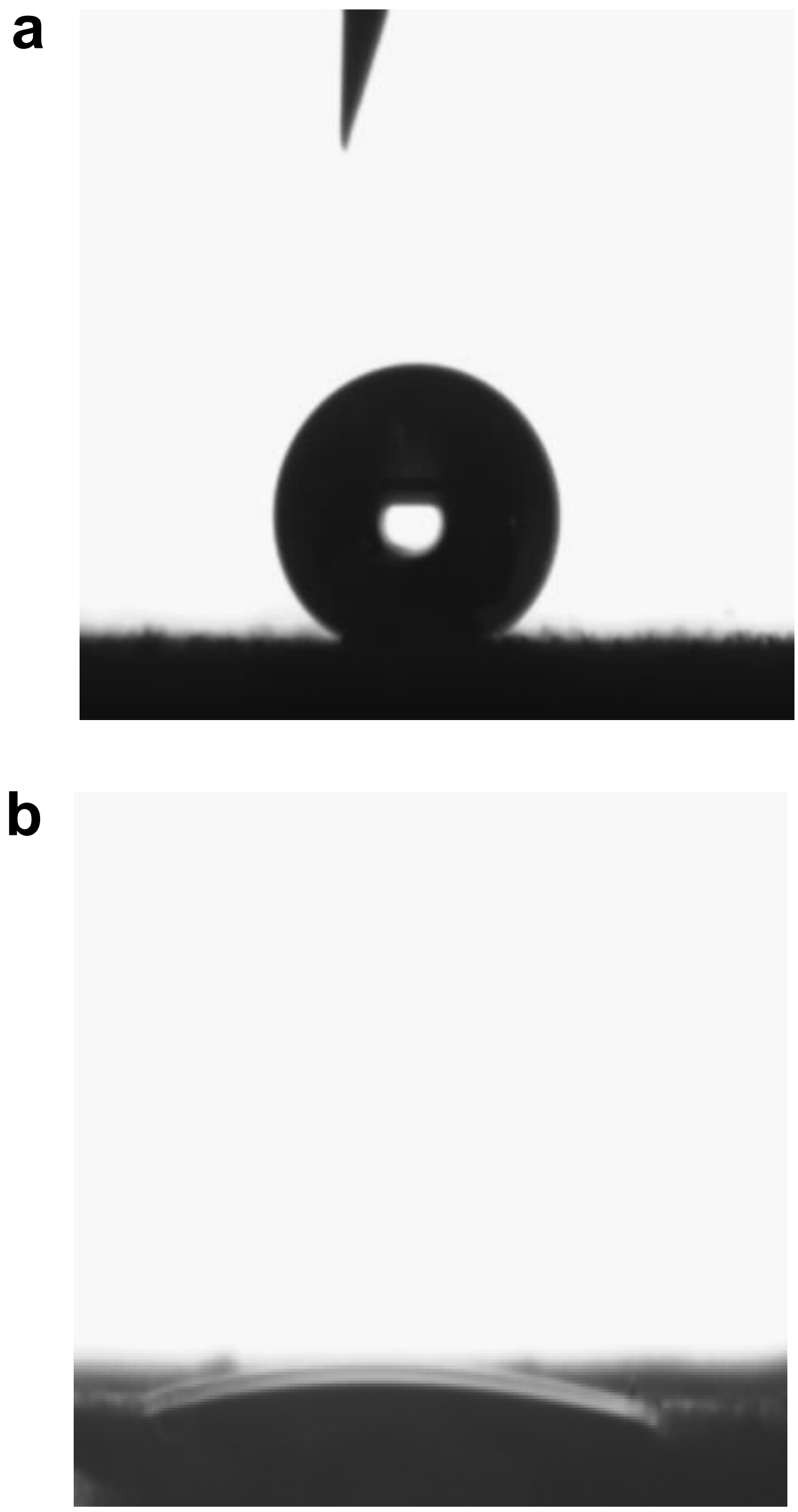

Figure S17. Water contact angle of PAM-15-5 (a) and PAF-525-9 (b).

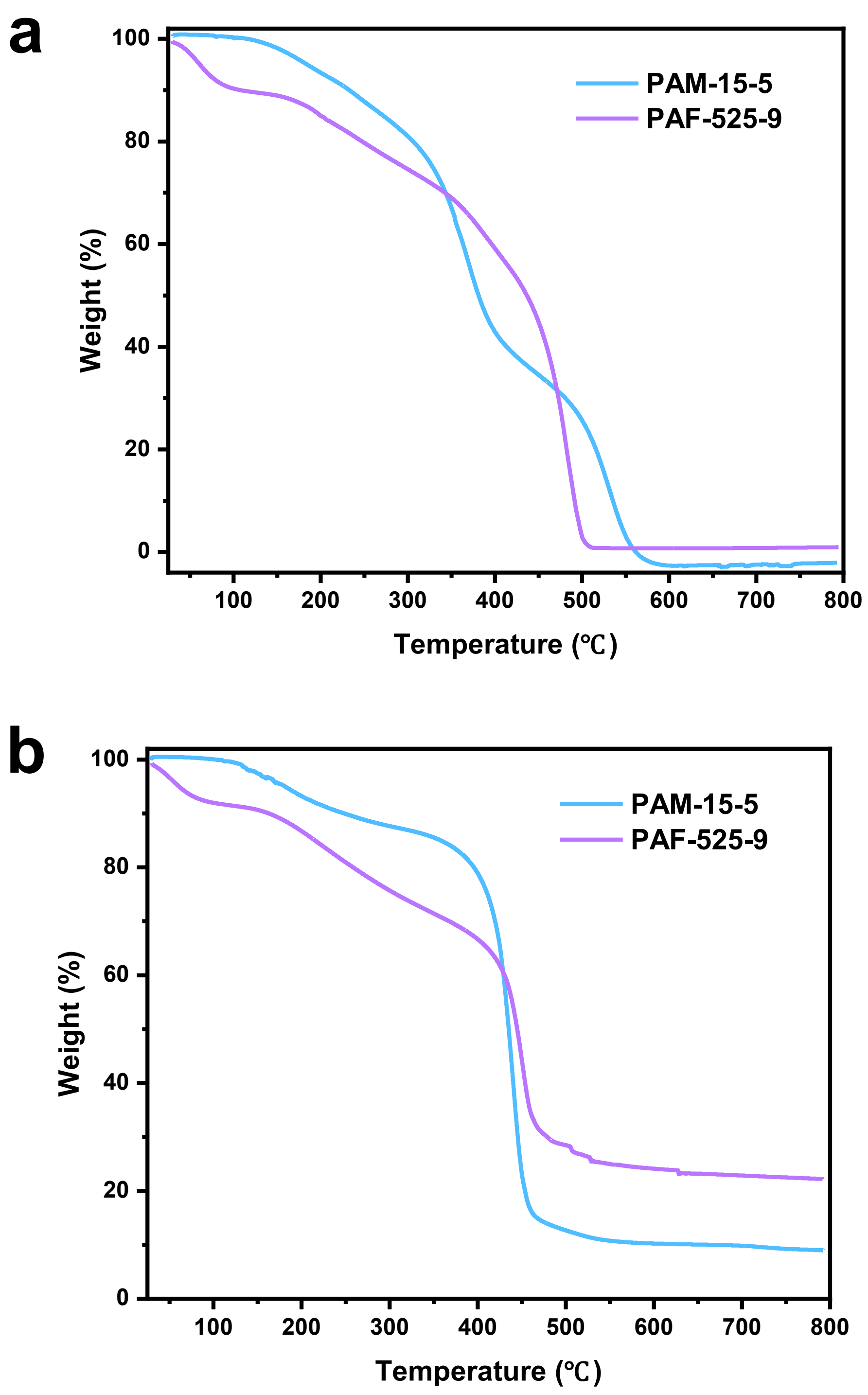

Figure S18. TGA profiles of PAM-15-5 and PAF-525-9 in air atmosphere (a) and nitrogen atmosphere (b).

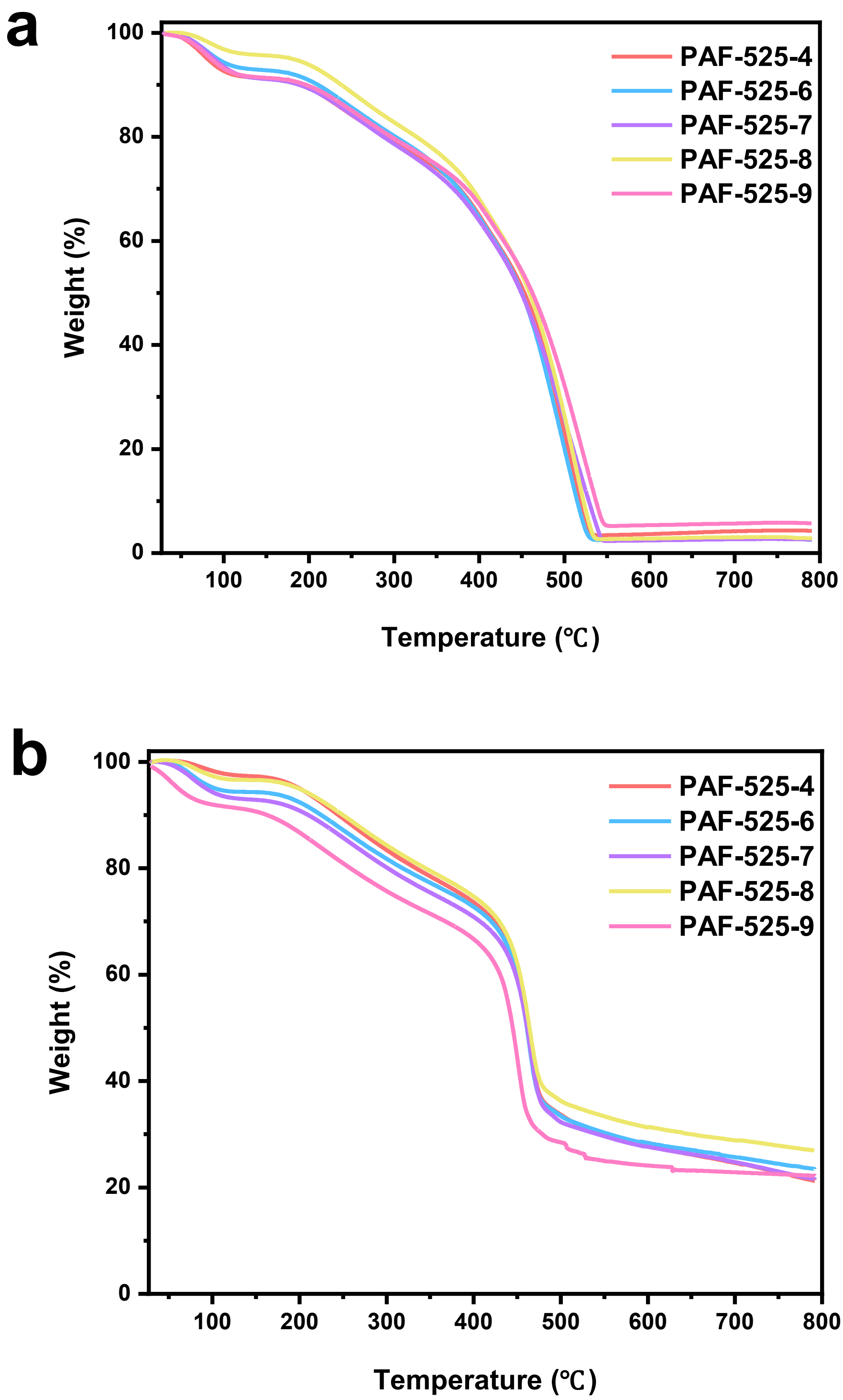

Figure S19. TGA profiles of PAFs materials in air atmosphere (a) and nitrogen atmosphere (b).

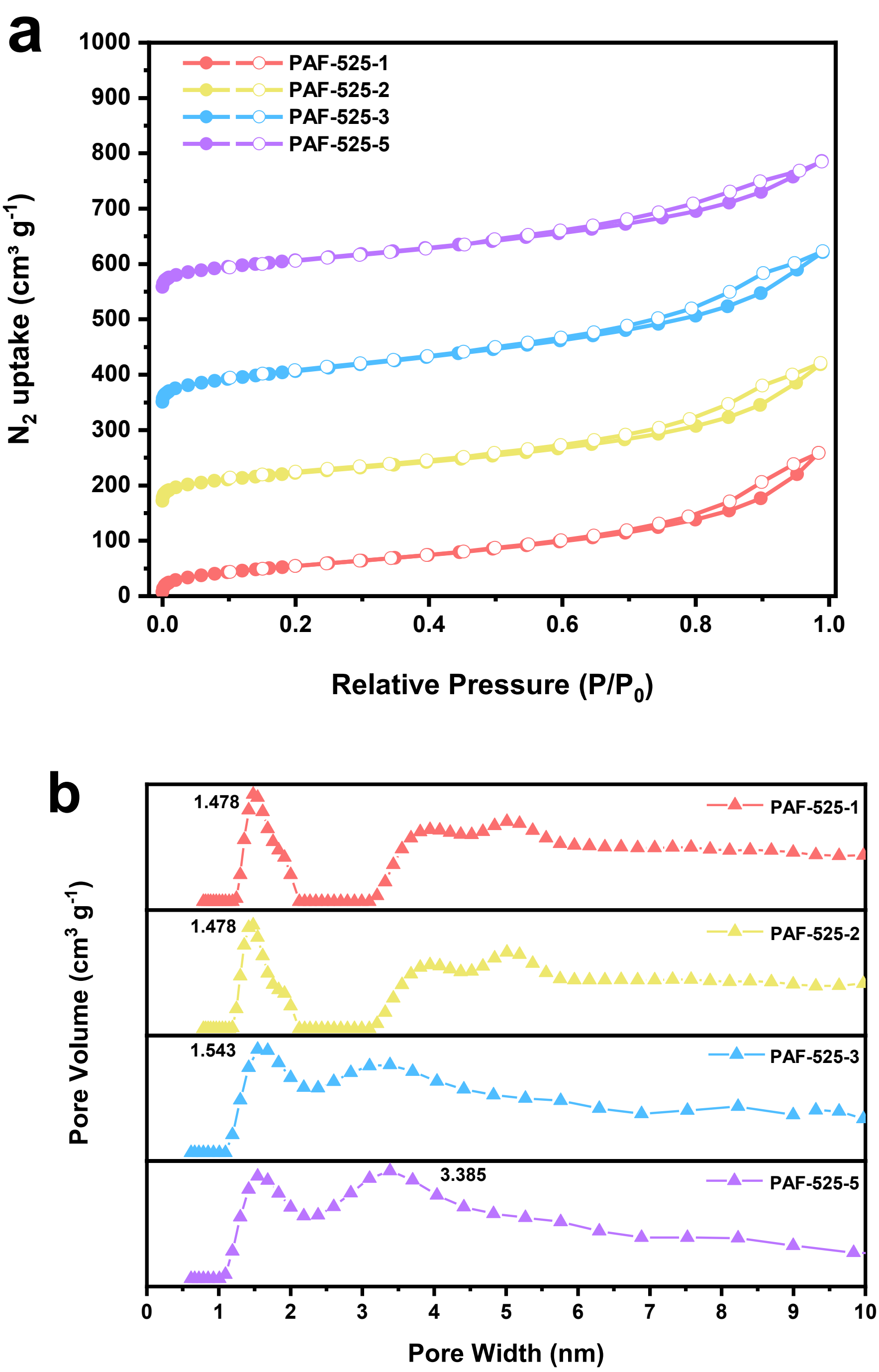

Figure S20. (a) N_2 adsorption and desorption isotherms of PAF-525-1-PAF-525-3 and PAF-525-5 at 77.3 K and (b) Pore size distributions of PAF-525-1-PAF-525-3 and PAF-525-5 calculated by using density functional theory (DFT) methods (slit pore models and differential pore volumes).

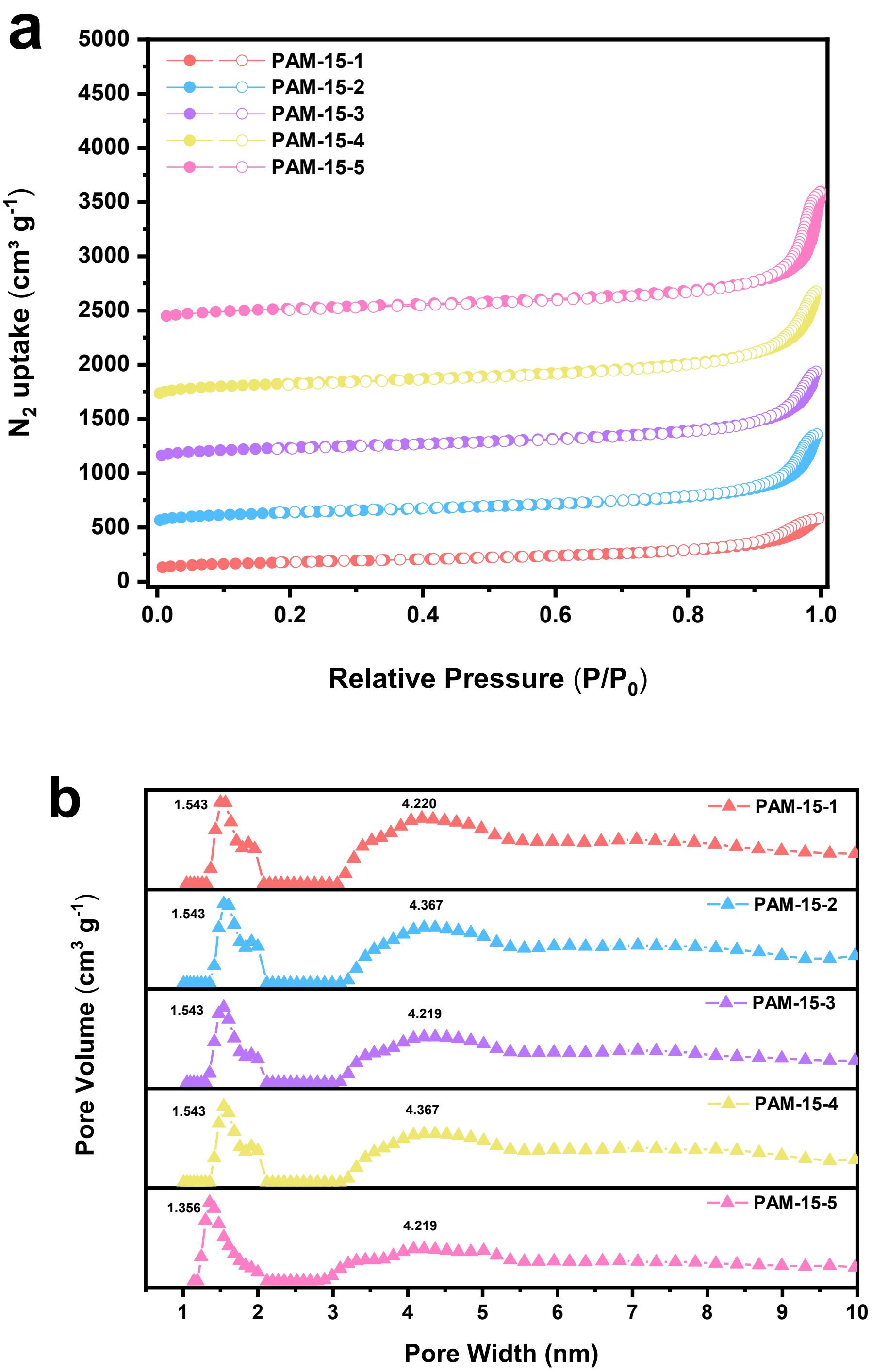

Figure S21. (a) N_2 adsorption and desorption isotherms of PAM-15-X at 77.3 K and (b) Pore size distributions of PAM-15-X calculated by using density functional theory (DFT) methods (slit pore models and differential pore volumes).

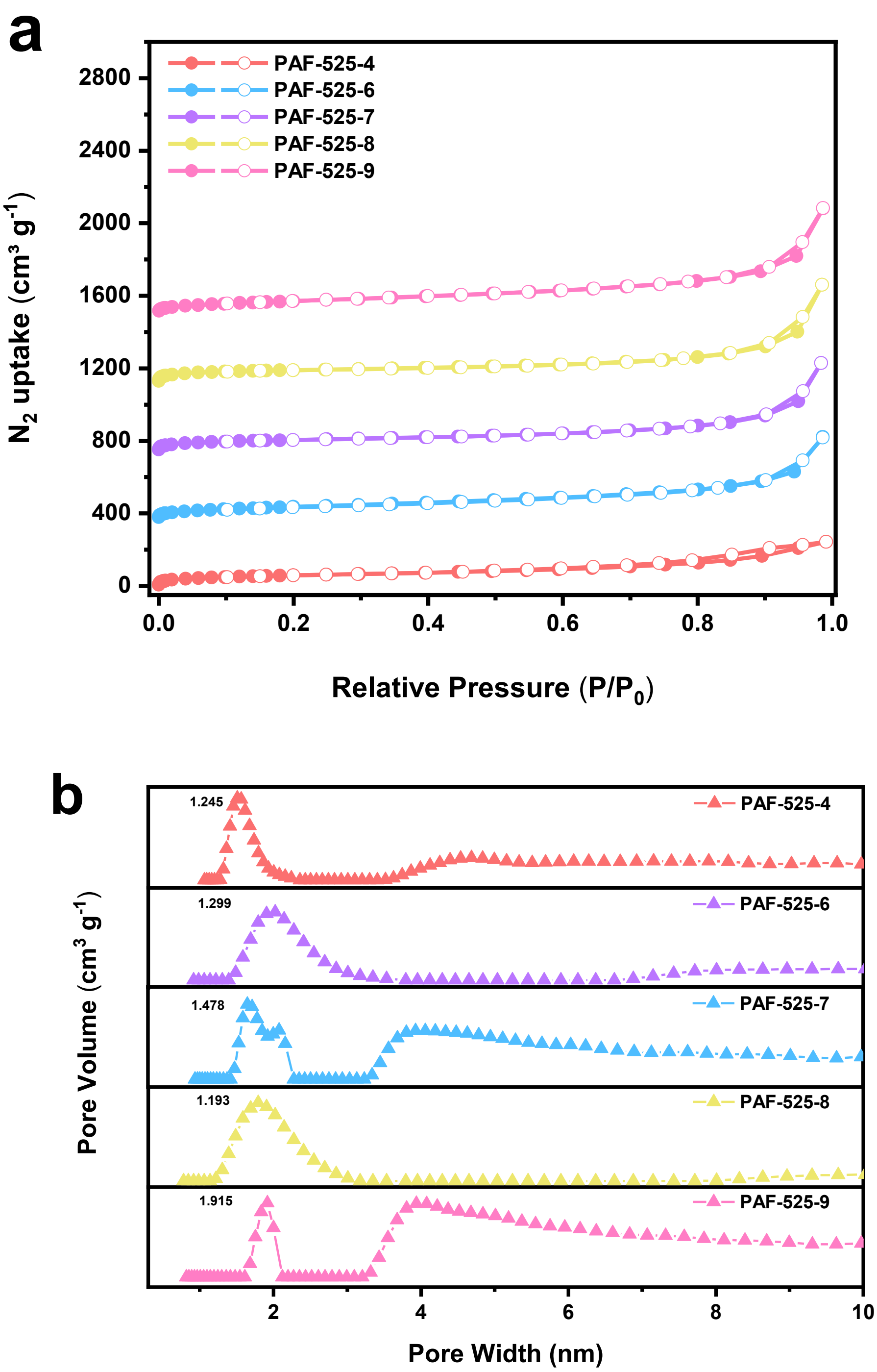

Figure S22. (a) N_2 adsorption and desorption isotherms of PAF-525-4 and PAF-525-6-PAF-525-9 at 77.3 K and (b) Pore size distributions of PAF-525-4 and PAF-525-6-PAF-525-9 calculated by using density functional theory (DFT) methods (slit pore models and differential pore volumes).

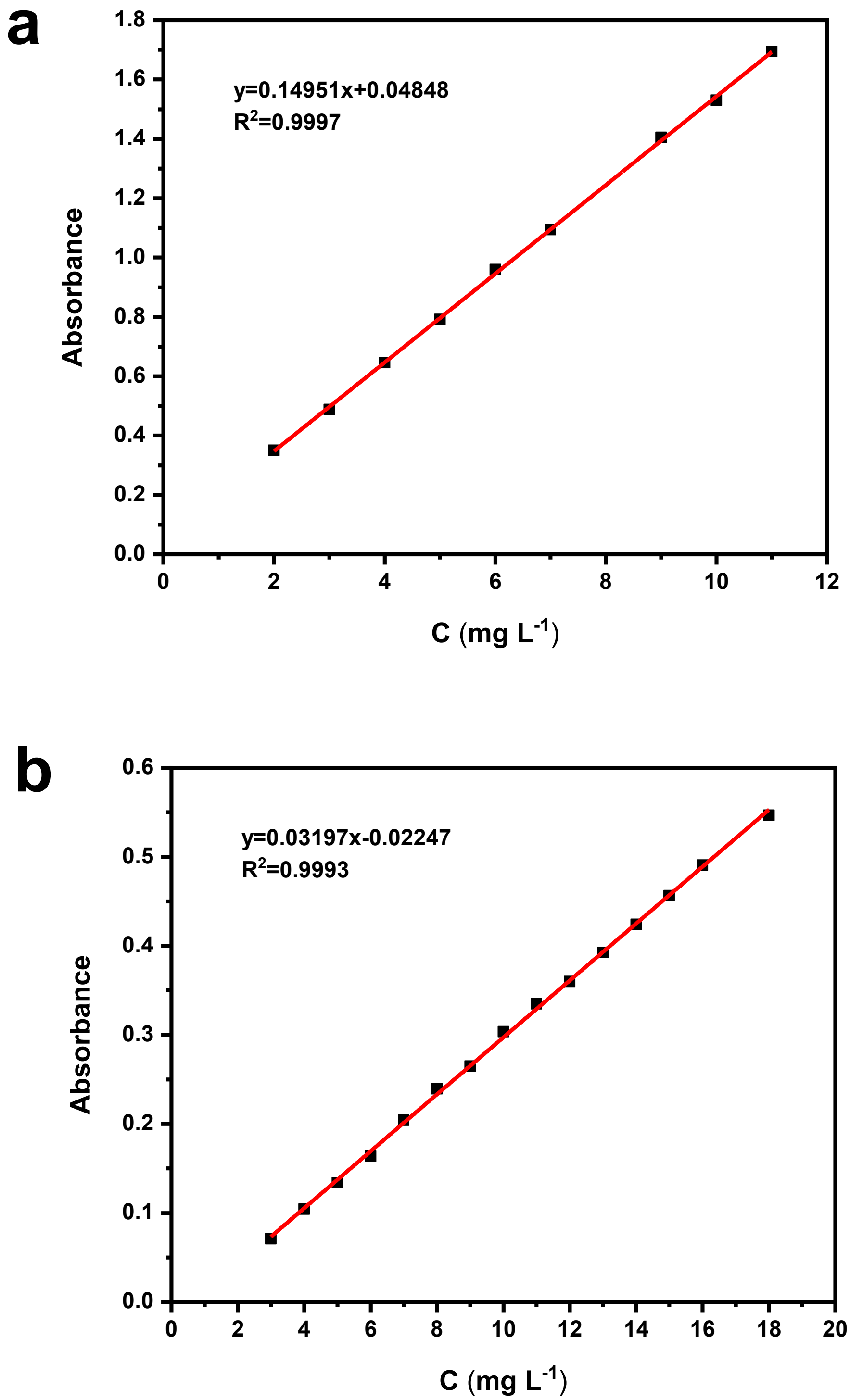

Figure S23. Standard curves of MB (a) and TC (b).

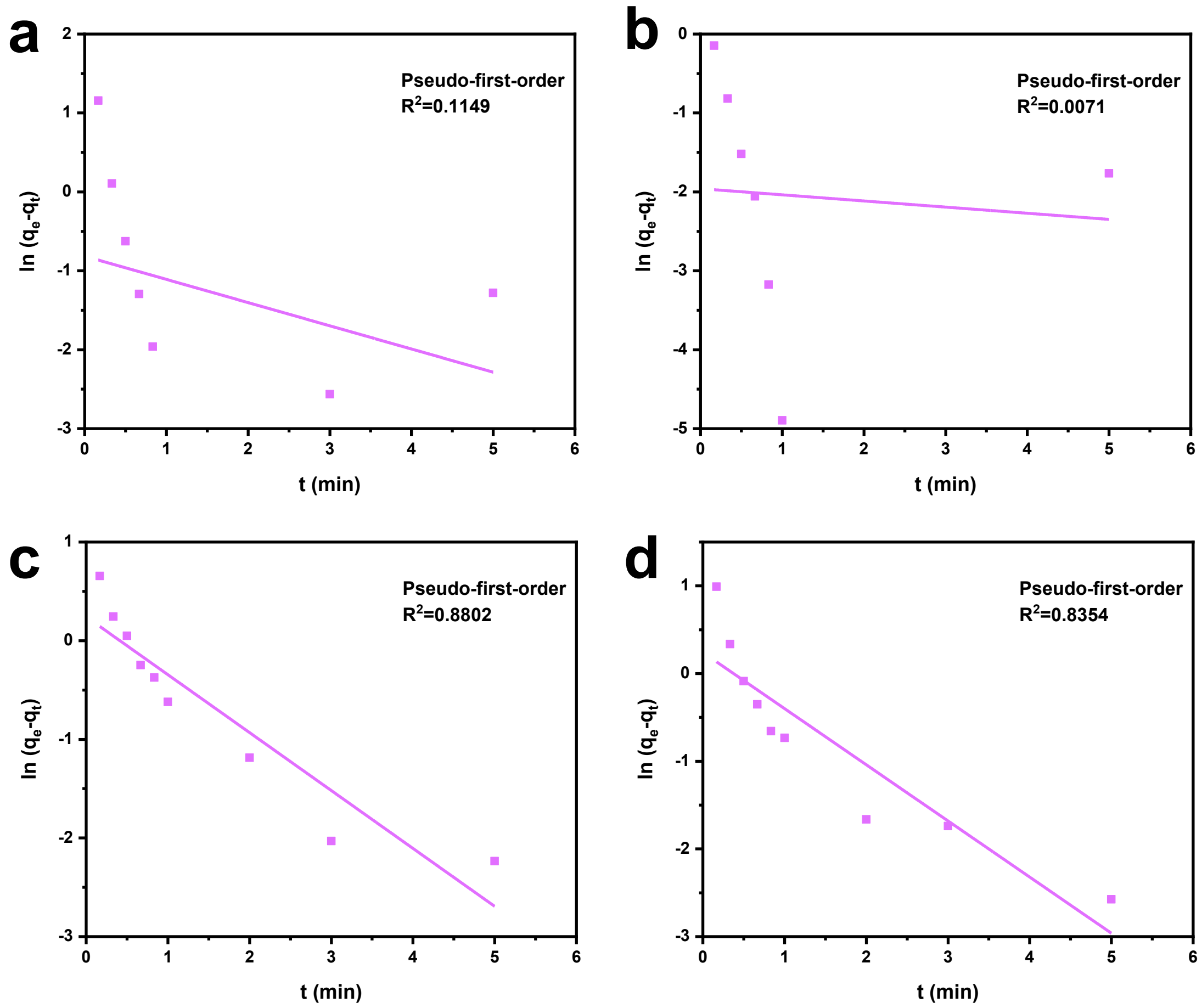

Figure S24. Pseudo-first-order kinetic data of PAF-525-6 (a), PAF-525-7 (b), PAF-525-8 (c) and PAF-525-9 (d) for MB at 200 ppm.

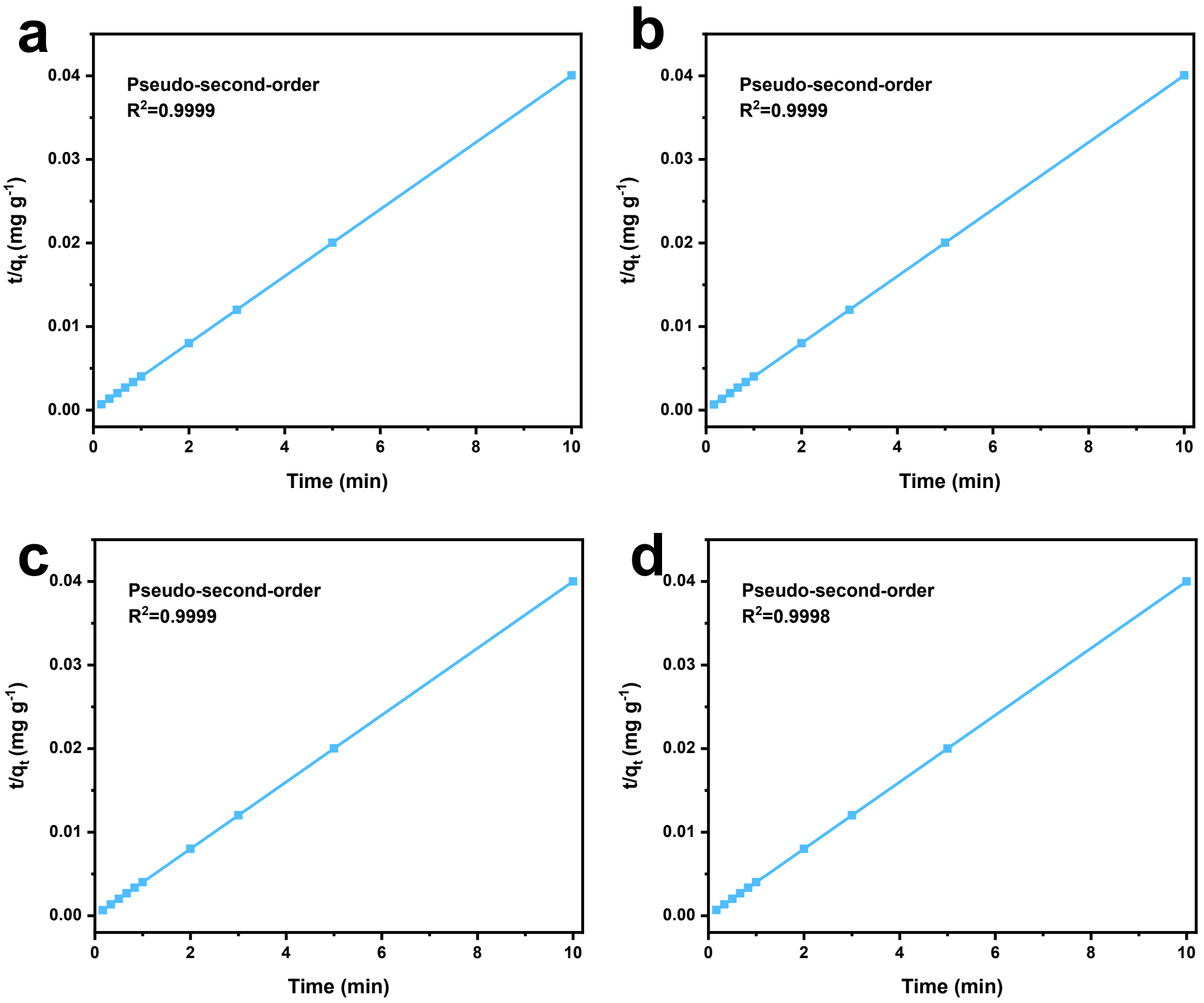

Figure S25. Pseudo-second-order kinetic data of PAF-525-6 (a), PAF-525-7 (b), PAF-525-8 (c) and PAF-525-9 (d) for MB at 200 ppm.

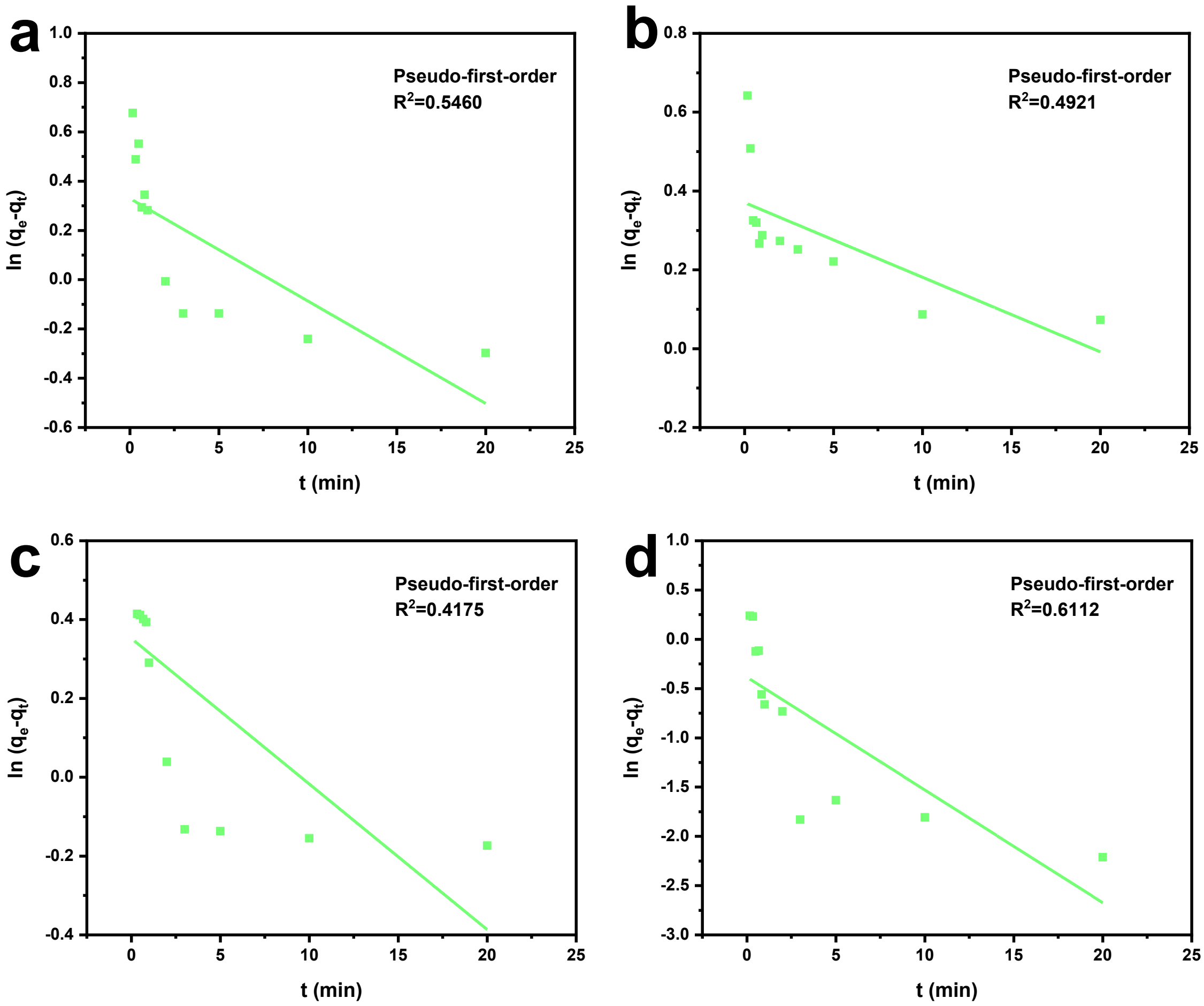

Figure S26. Pseudo-first-order kinetic data of PAF-525-6 (a), PAF-525-7 (b), PAF-525-8 (c) and PAF-525-9 (d) for TC at 50 ppm.

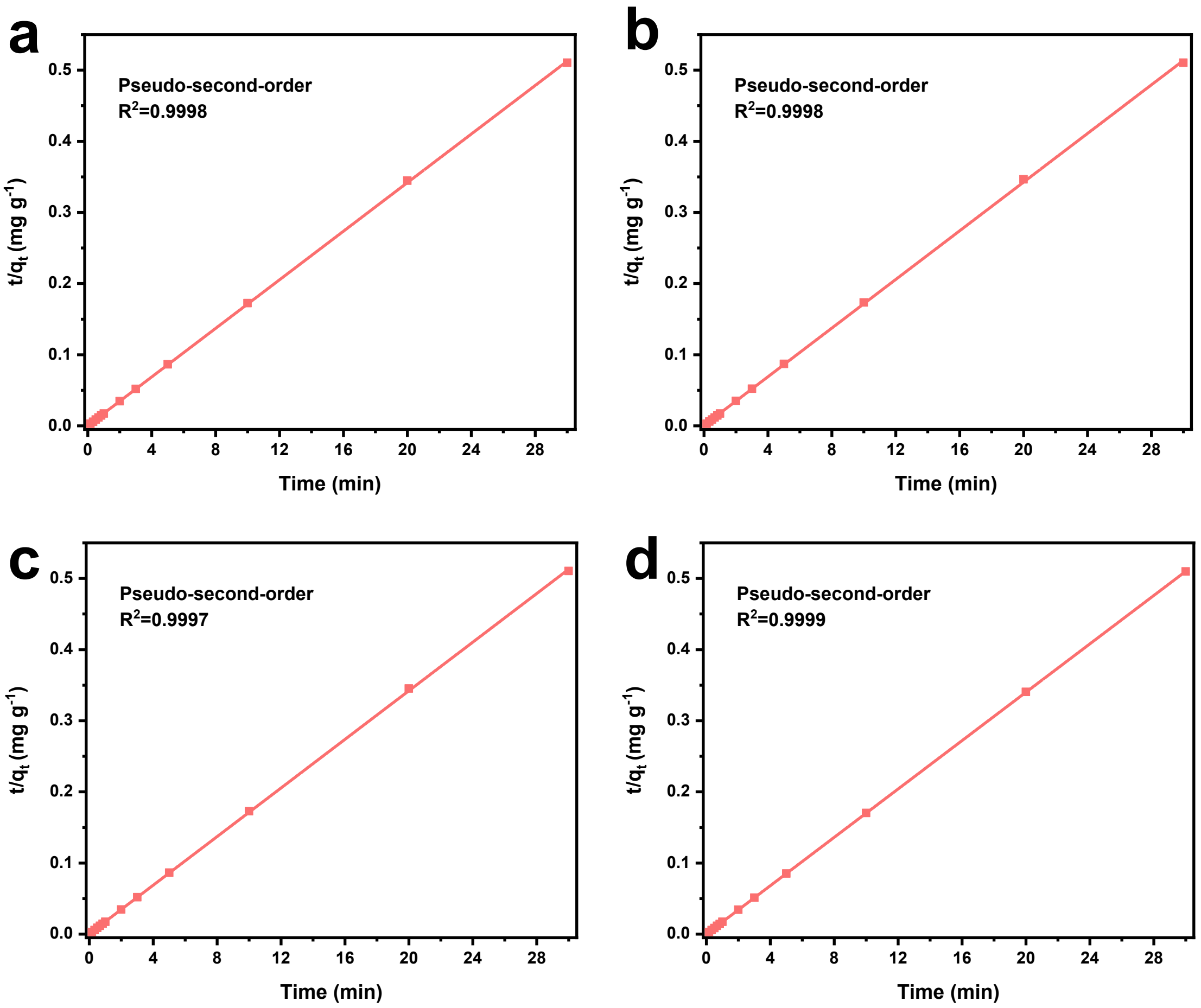

Figure S27. Pseudo-second-order kinetic data of PAF-525-6 (a), PAF-525-7 (b), PAF-525-8 (c) and PAF-525-9 (d) for TC at 50 ppm.

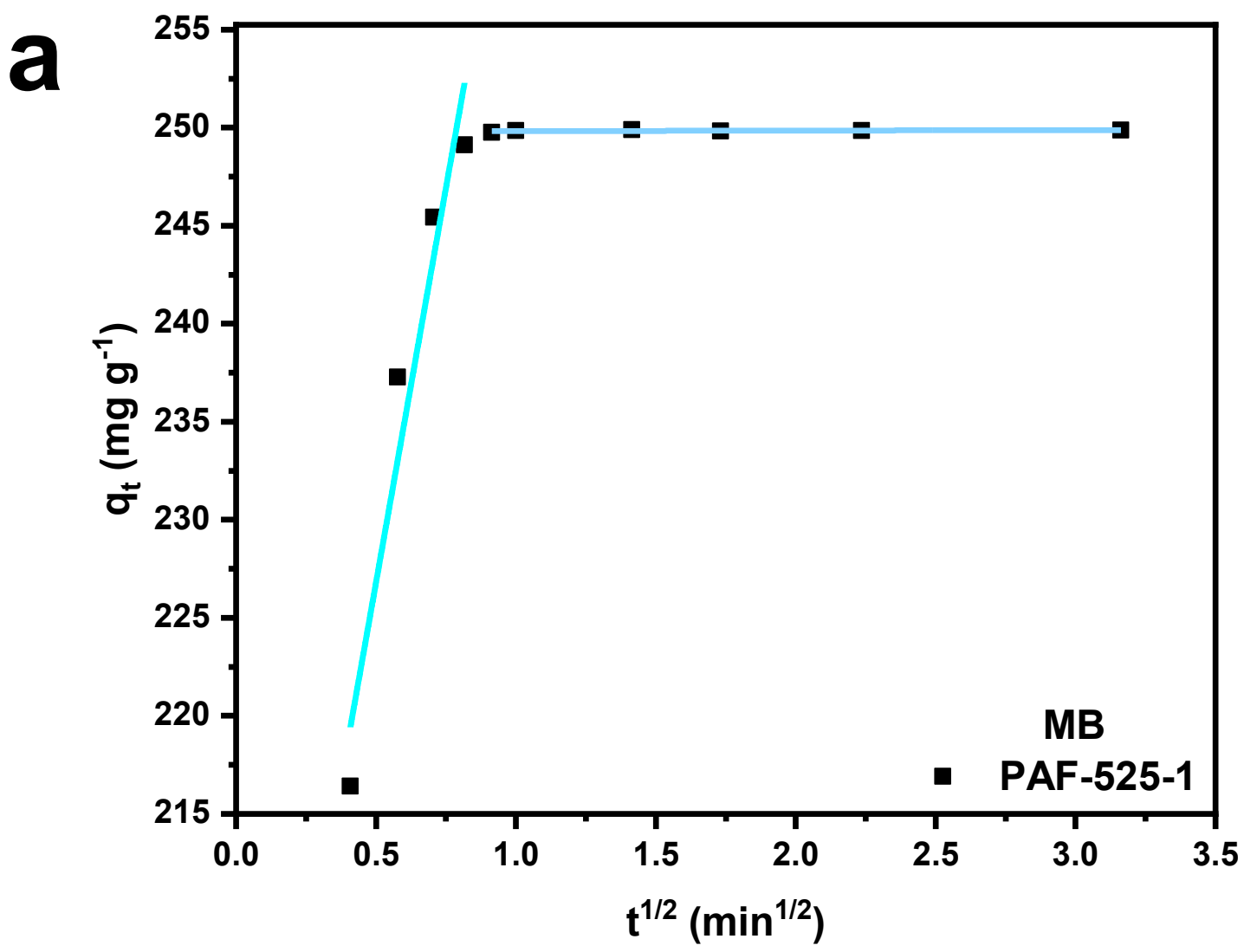

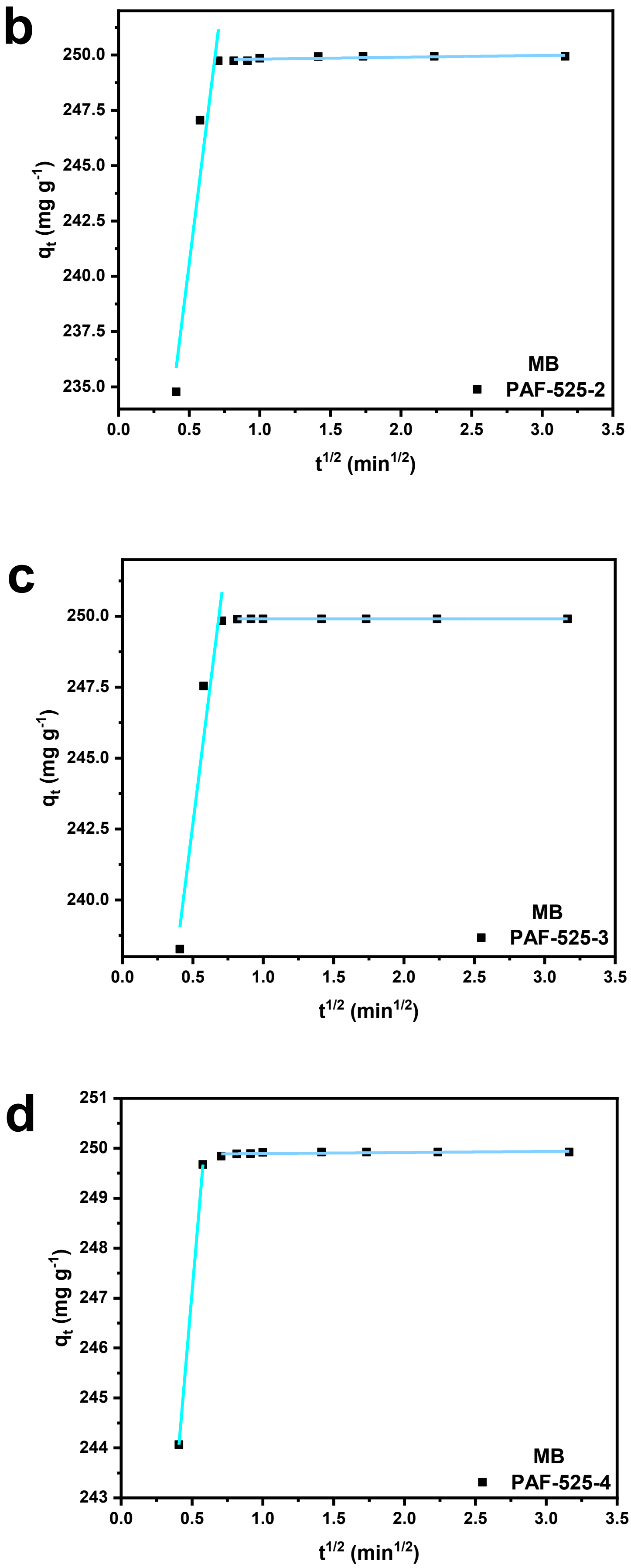

b
250.0
247.5
245.0
242.5
240.0
237.5
235.0
q_t (mg g^{-1})
0.0 0.5 1.0 1.5 2.0 2.5 3.0 3.5
$t^{1/2}$ ($min^{1/2}$)
MB
PAF-525-2
c
250.0
247.5
245.0
242.5
240.0
q_t (mg g^{-1})
0.0 0.5 1.0 1.5 2.0 2.5 3.0 3.5
$t^{1/2}$ ($min^{1/2}$)
MB
PAF-525-3
d
251
250
249
248
247
246
245
244
243
q_t (mg g^{-1})
0.0 0.5 1.0 1.5 2.0 2.5 3.0 3.5
$t^{1/2}$ ($min^{1/2}$)
MB
PAF-525-4

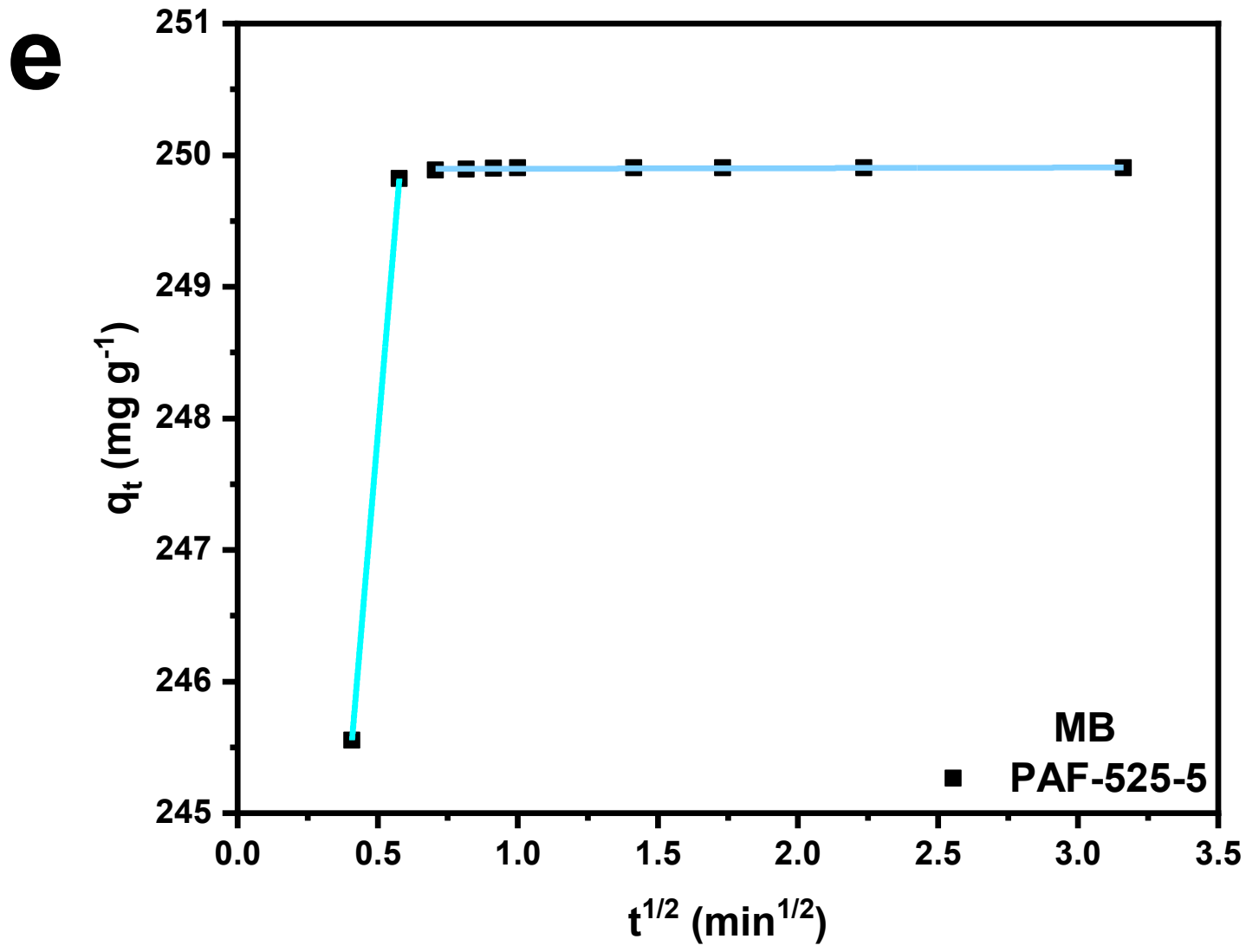

Figure S28. Intraparticle diffusion curve of PAF-525-1-PAF-525-5 for MB.

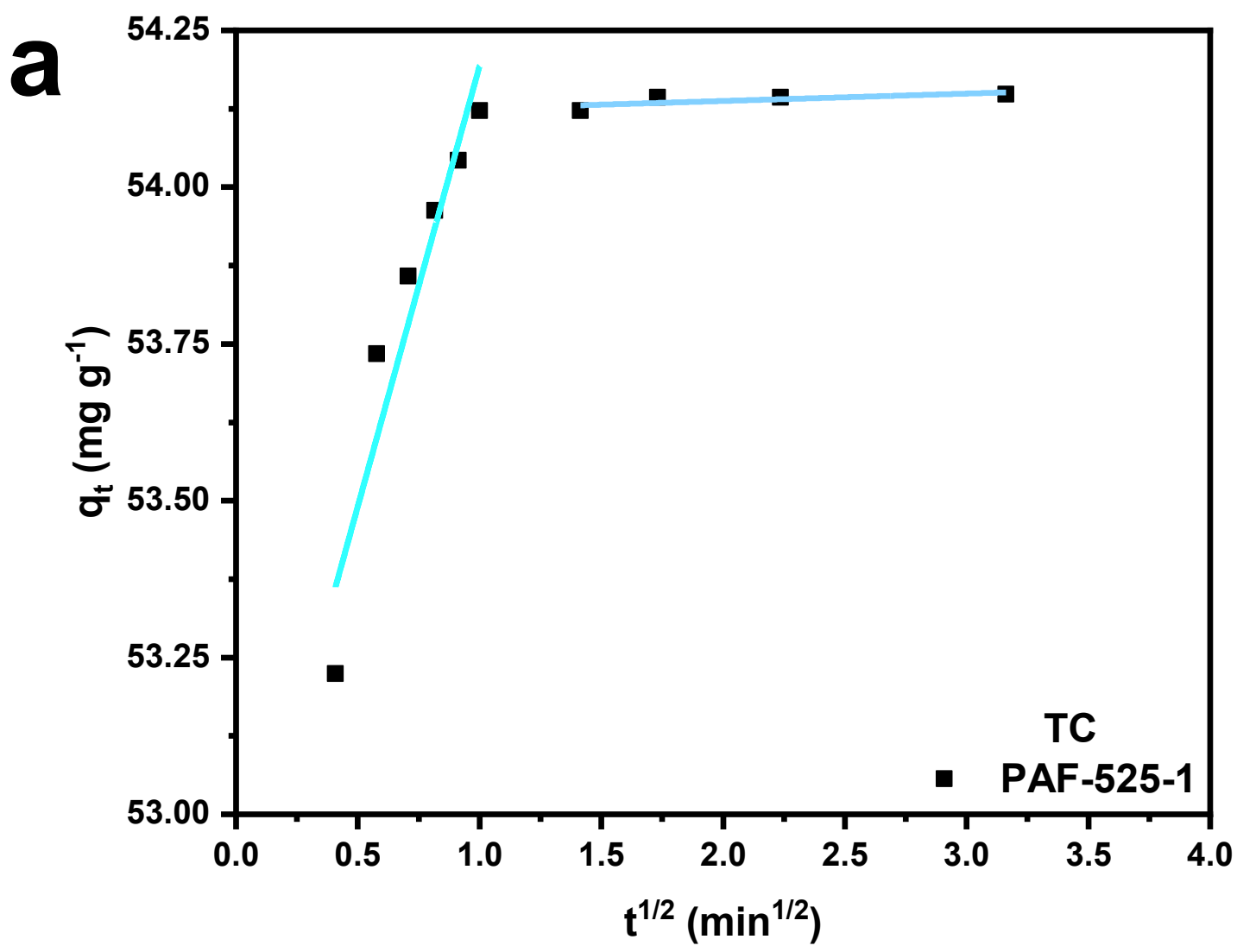

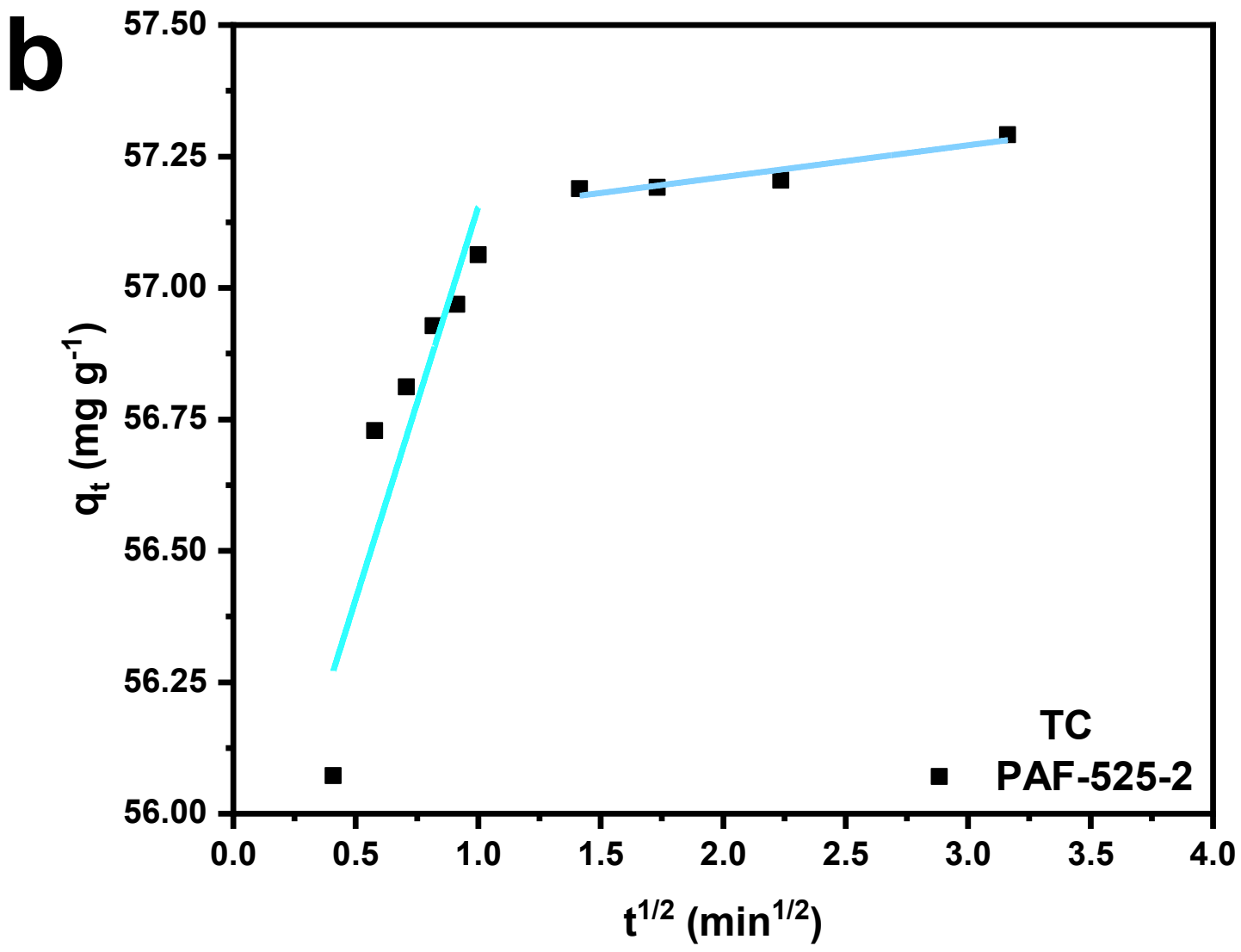

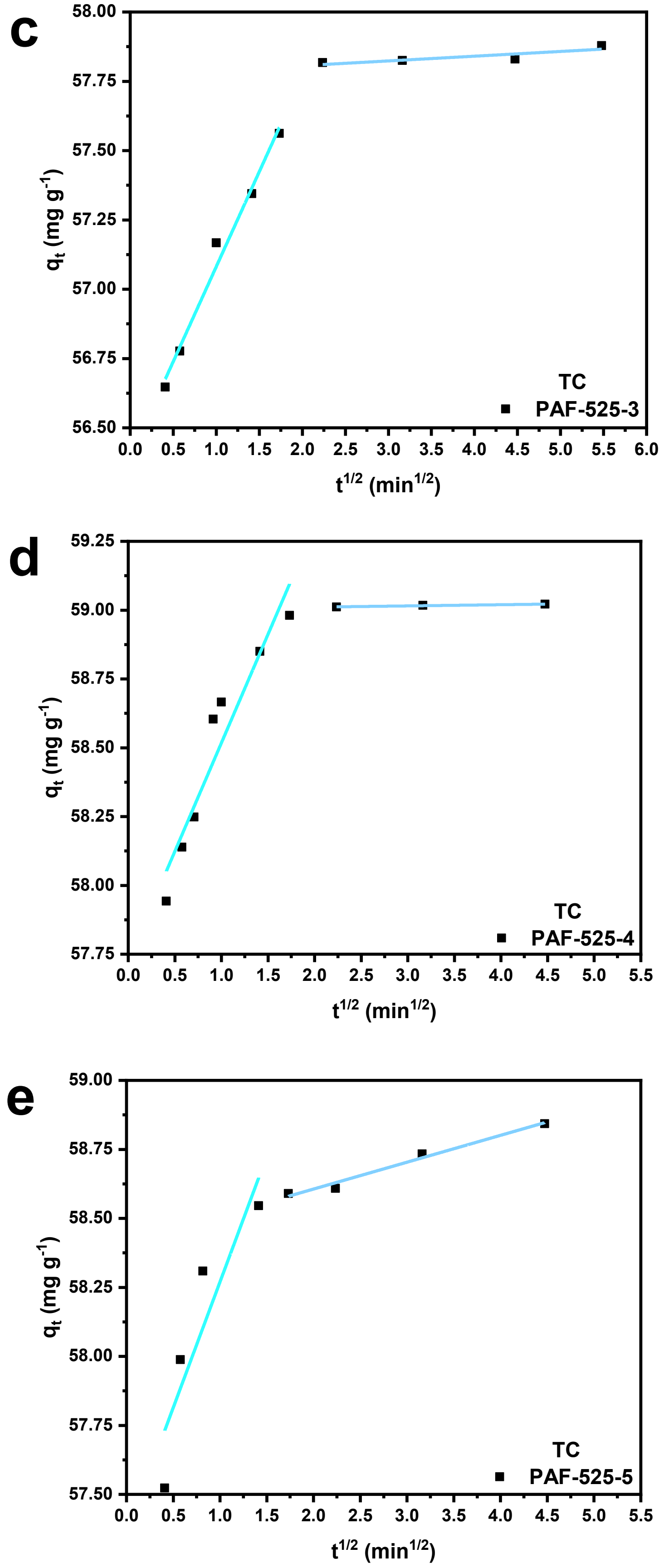

Figure S29. Intraparticle diffusion curve of PAF-525-1-PAF-525-5 for TC.

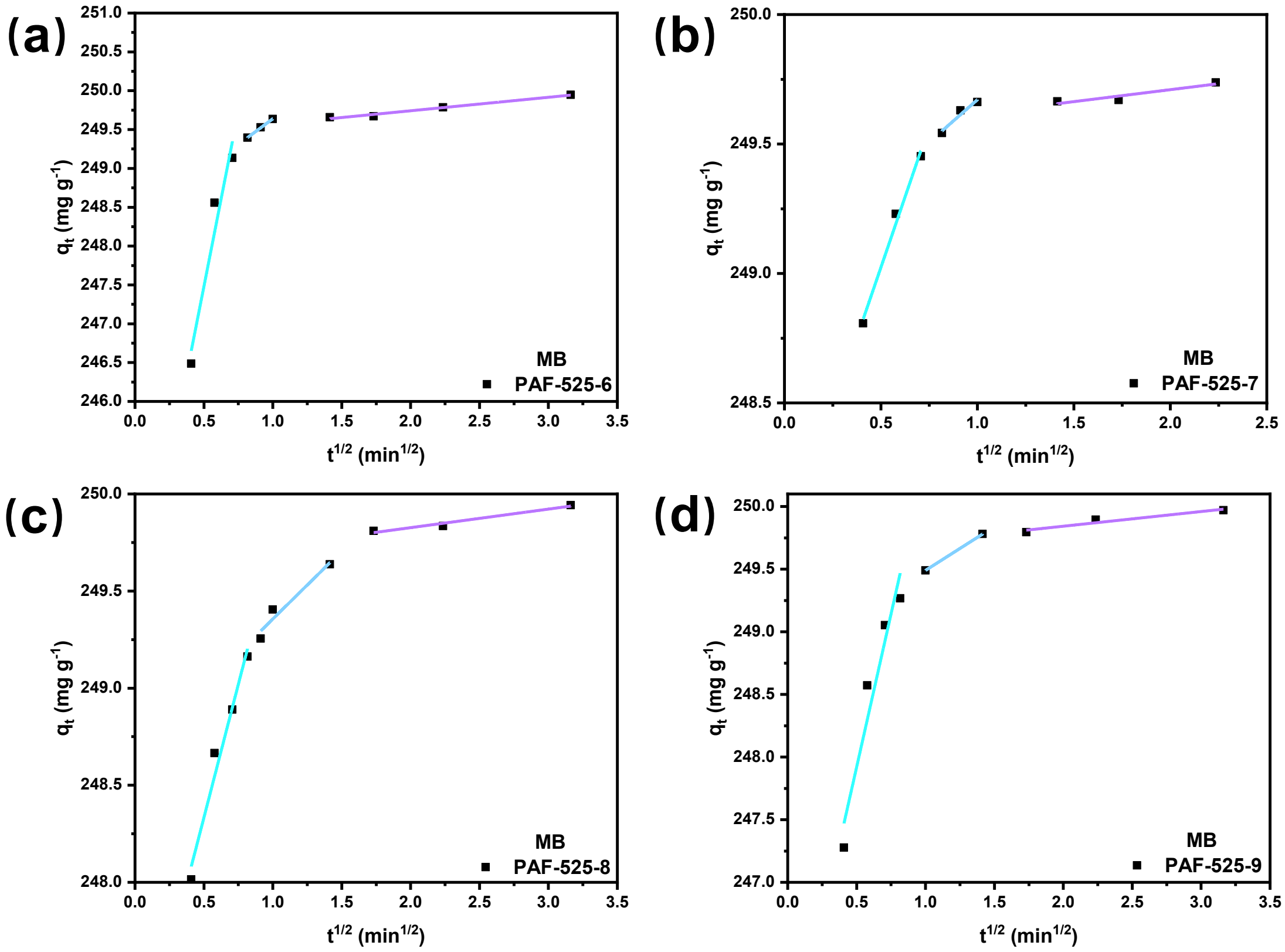

Figure S30. Intraparticle diffusion curve of PAF-525-6-PAF-525-9 for MB.

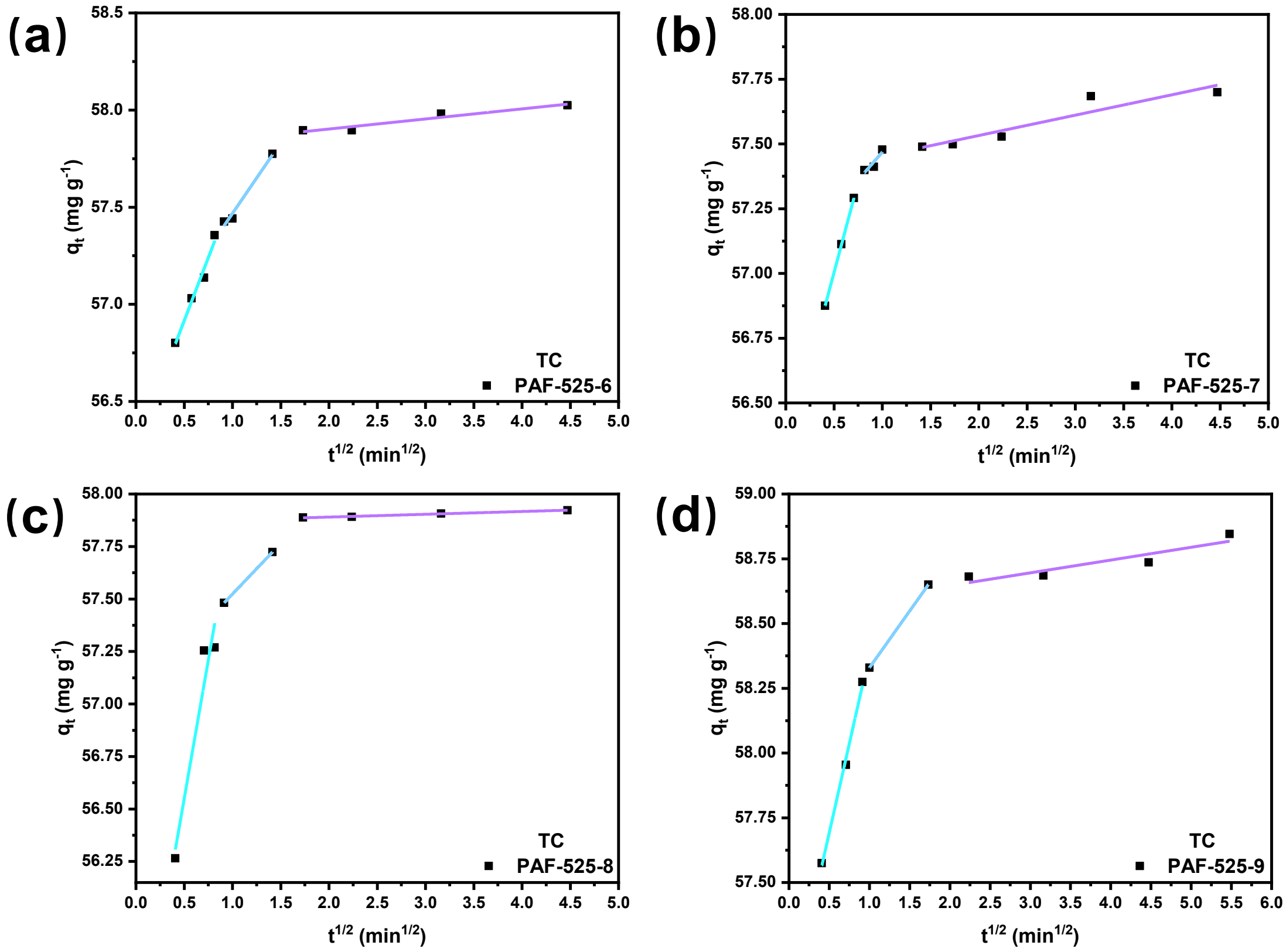

Figure S31. Intraparticle diffusion curve of PAF-525-6-PAF-525-9 for TC.

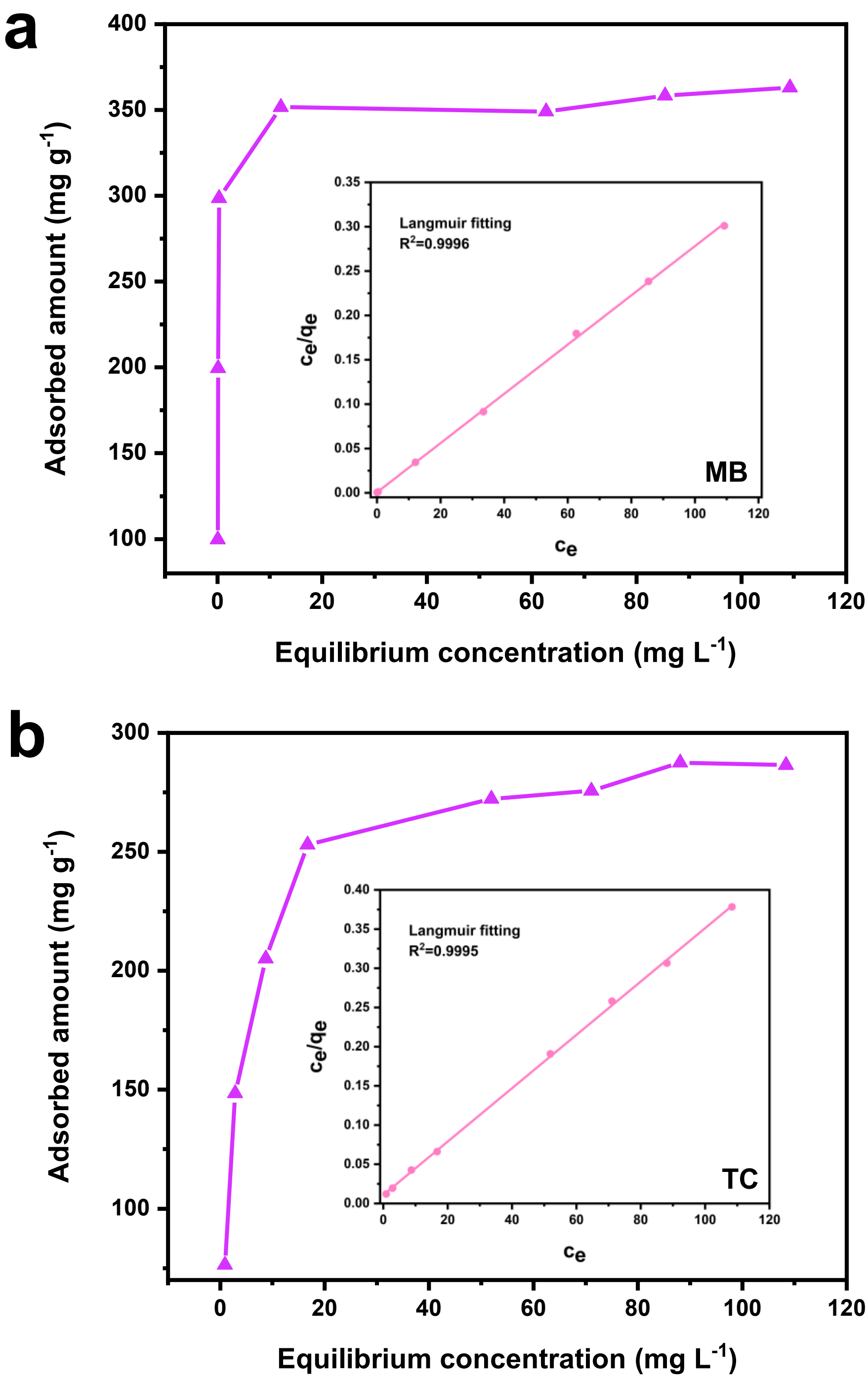

Figure S32. Relationship between equilibrium adsorption capacities and equilibrium concentrations, and the Langmuir isotherm model fitting curves of MB (a) and TC (b).

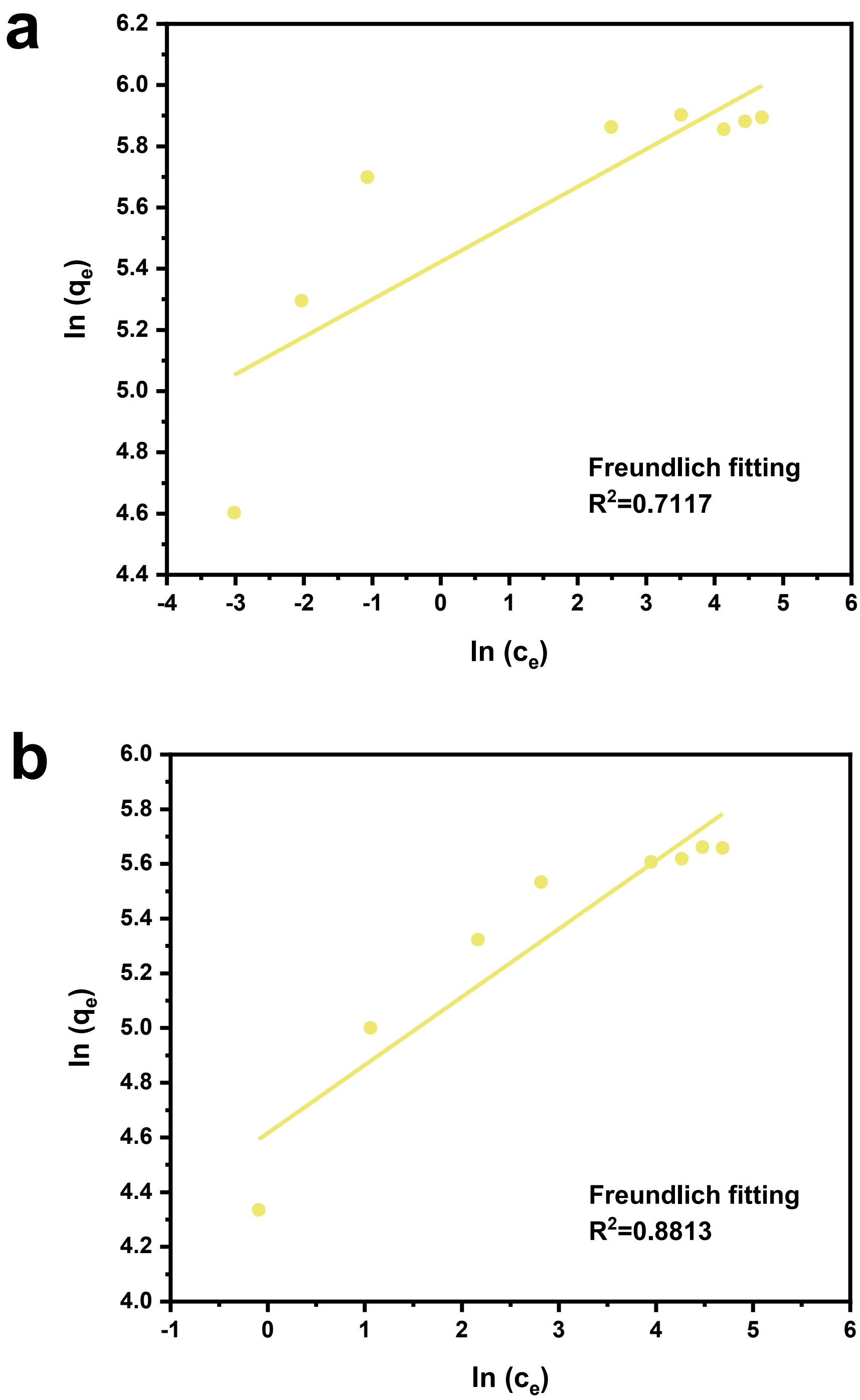

Figure S33. The Freundlich isotherm model fitting curves of MB (a) and TC (b).

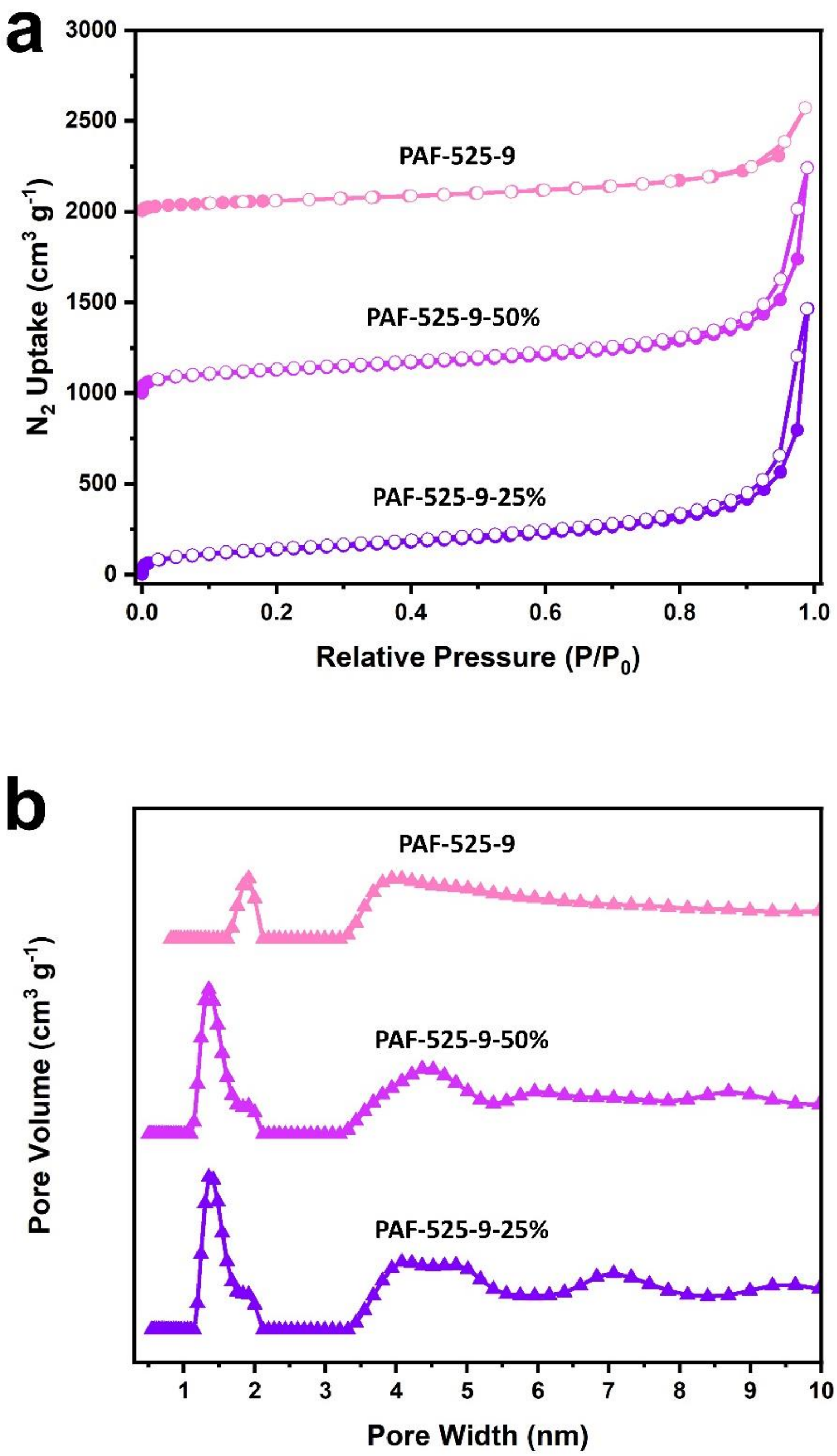

Figure S34. (a) N_2 adsorption and desorption isotherms of PAF-525-9, PAF-525-9-50% and PAF-525-9-25% at 77.3 K and (b) Pore size distributions of PAF-525-9, PAF-525-9-50% and PAF-525-9-25% calculated by using density functional theory (DFT) methods (slit pore models and differential pore volumes).

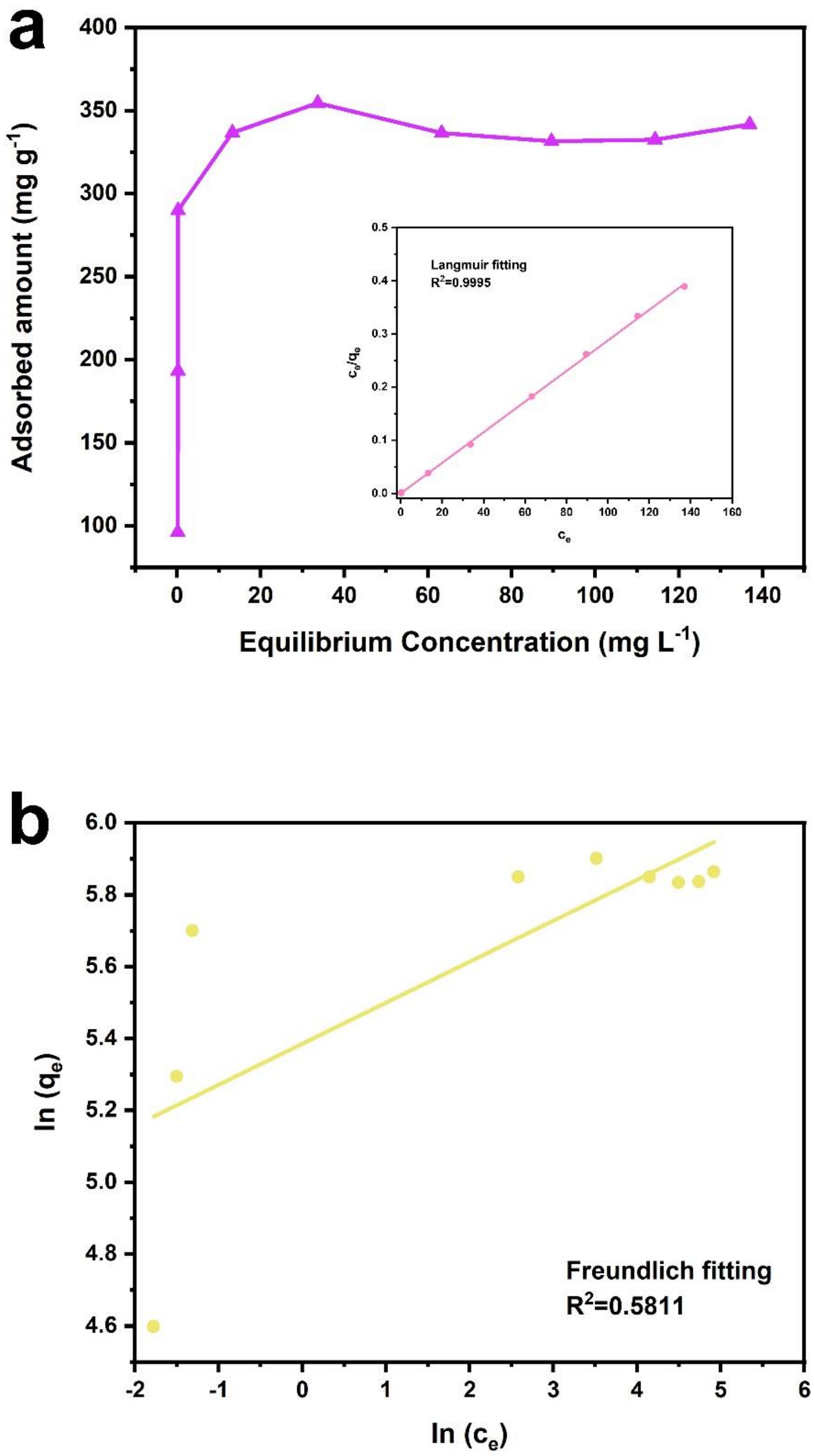

Figure S35. Relationship between equilibrium adsorption capacities and equilibrium concentrations, and the Langmuir isotherm model fitting curves (a) and the Freundlich isotherm model fitting curves (b) of PAF-525-9-50% toward MB.

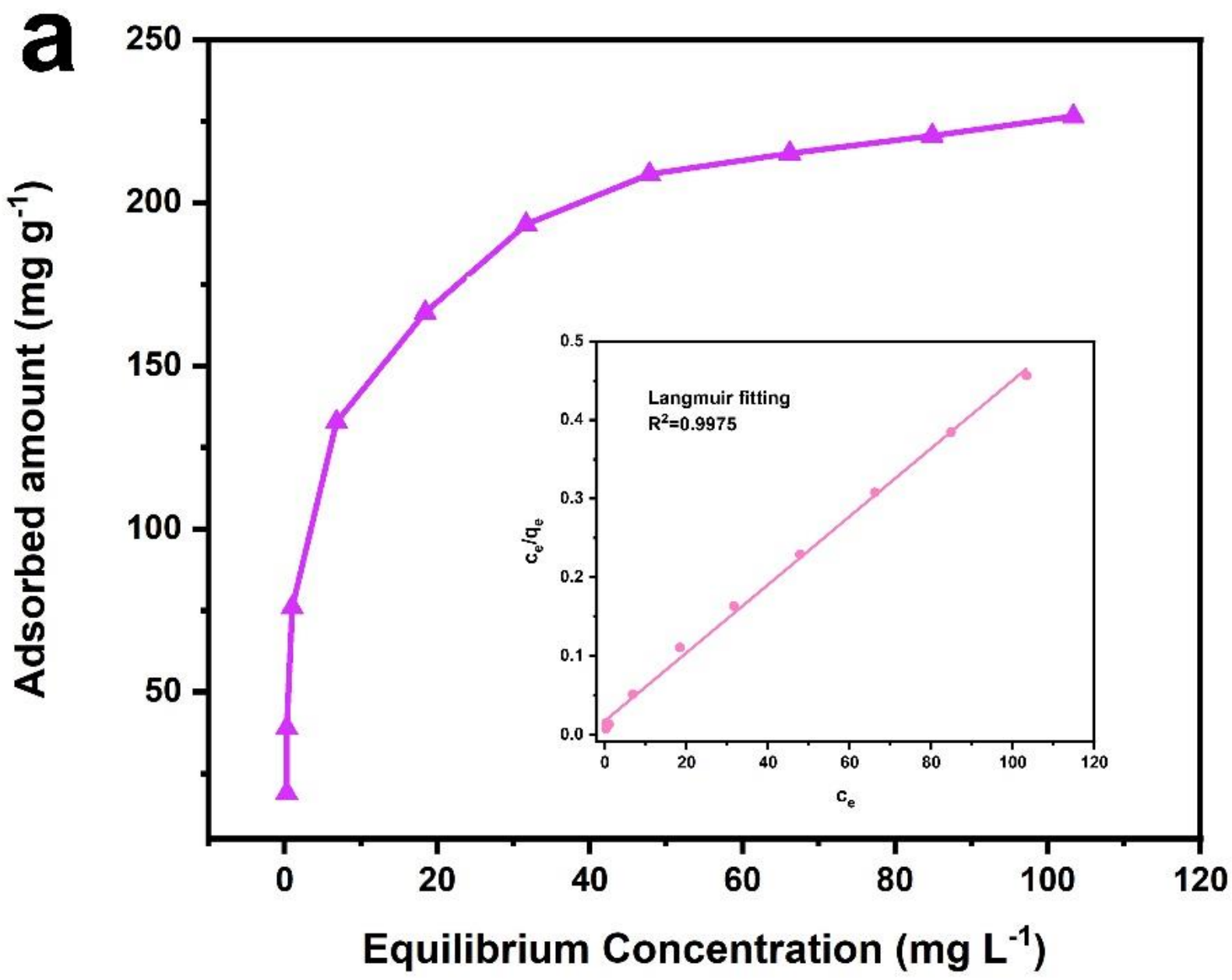

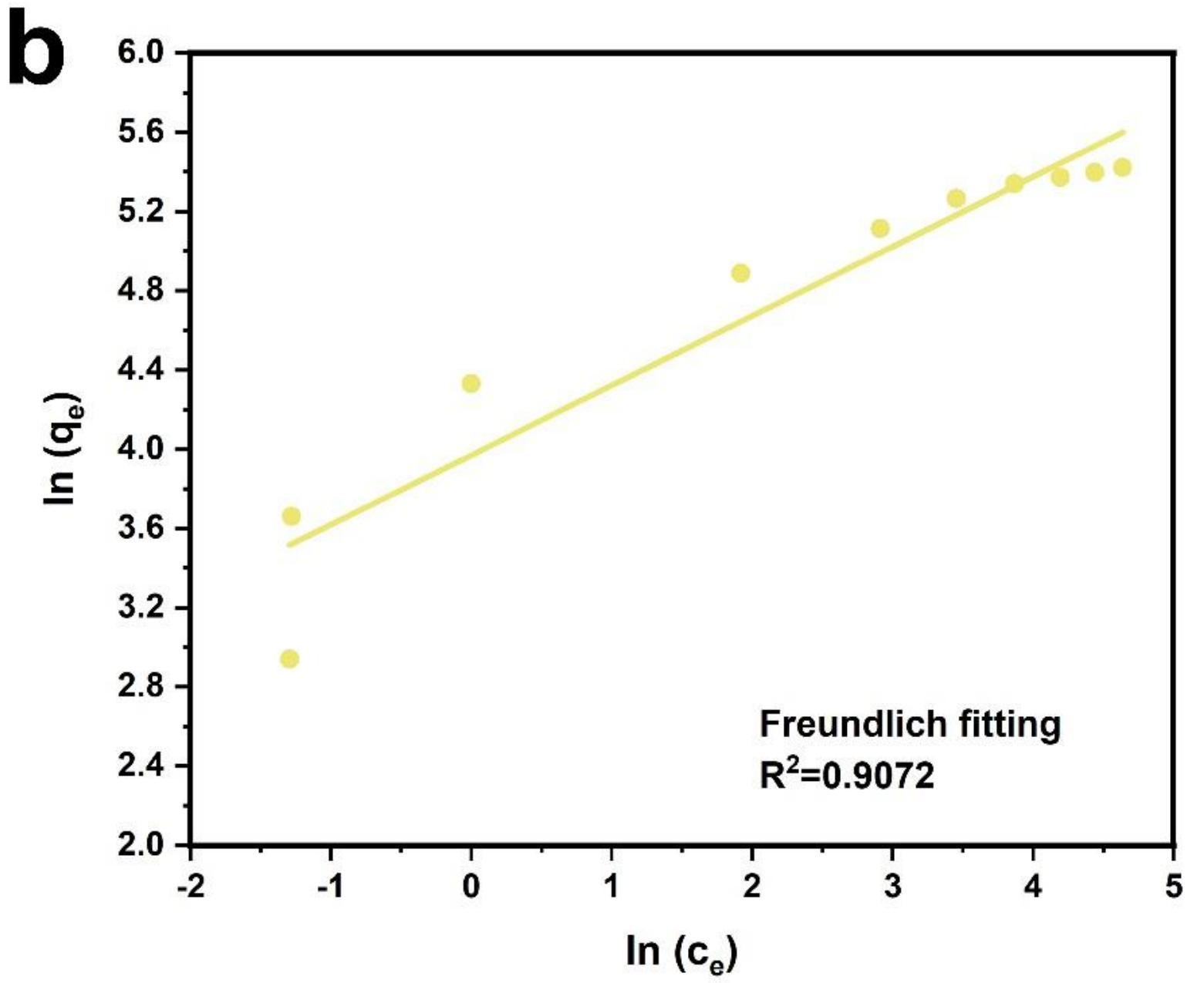

Figure S36. Relationship between equilibrium adsorption capacities and equilibrium concentrations, and the Langmuir isotherm model fitting curves (a) and the Freundlich isotherm model fitting curves (b) of PAF-525-9-50% toward TC.

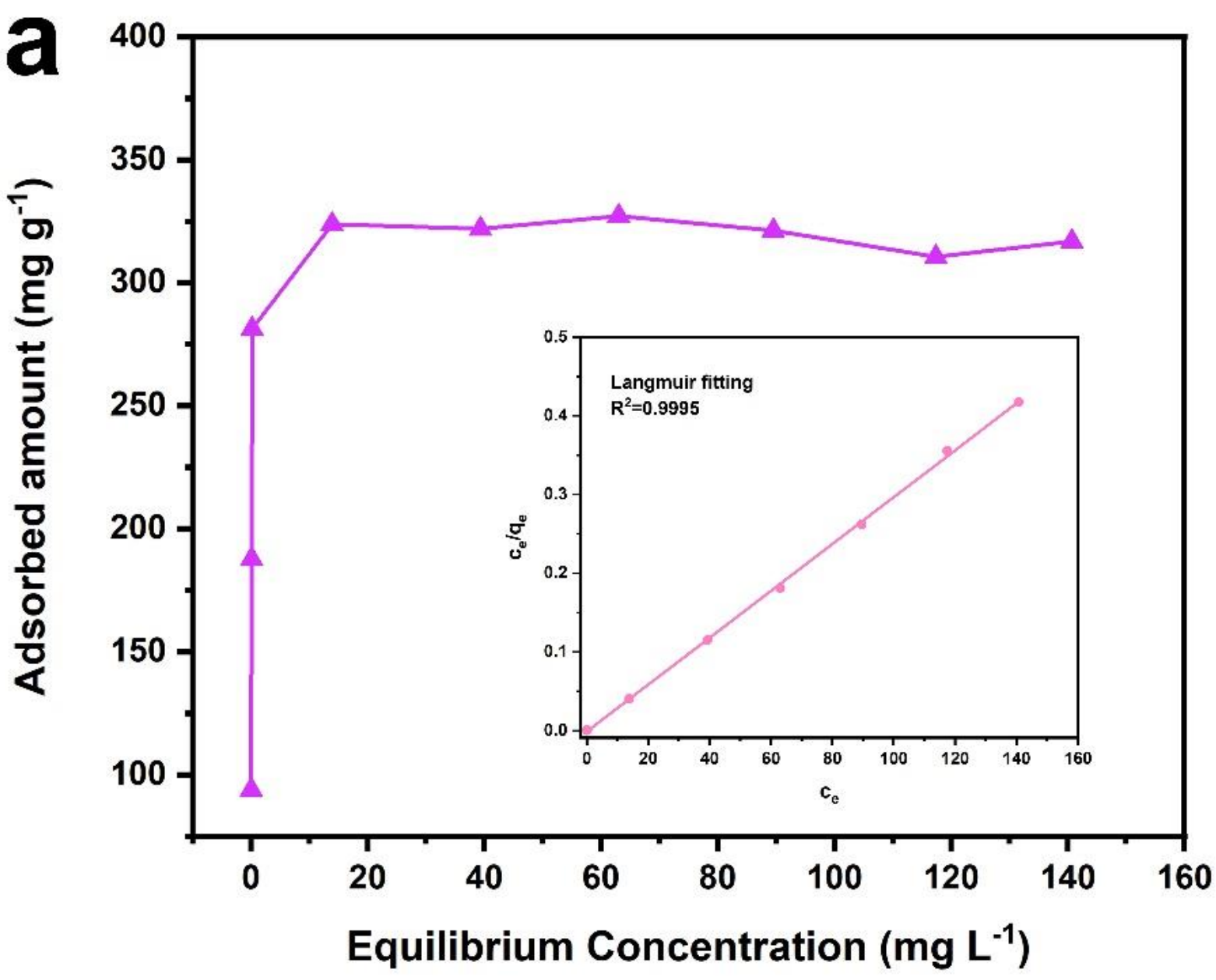

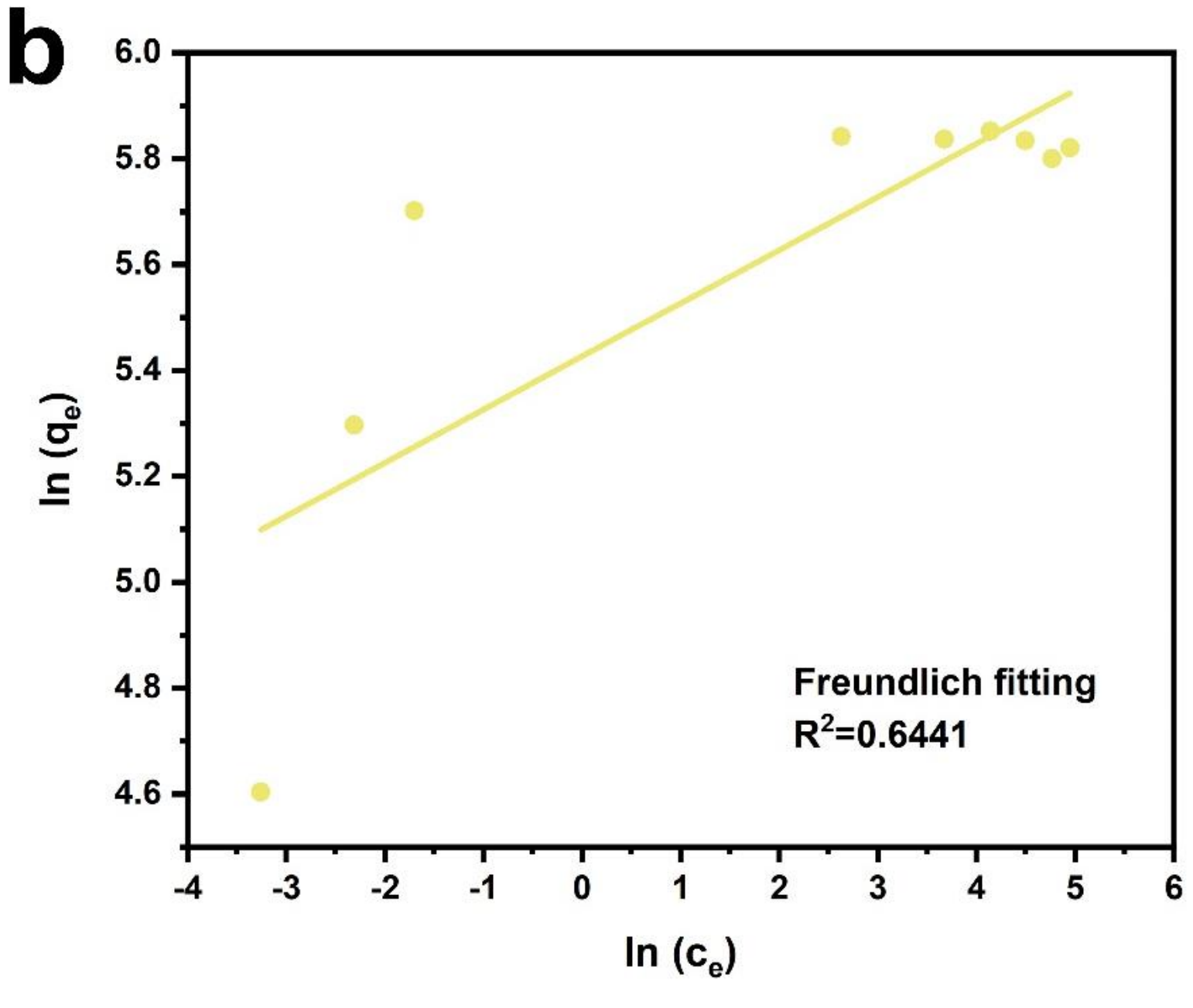

Figure S37. Relationship between equilibrium adsorption capacities and equilibrium concentrations, and the Langmuir isotherm model fitting curves (a) and the Freundlich isotherm model fitting curves (b) of PAF-525-9-25% toward MB.

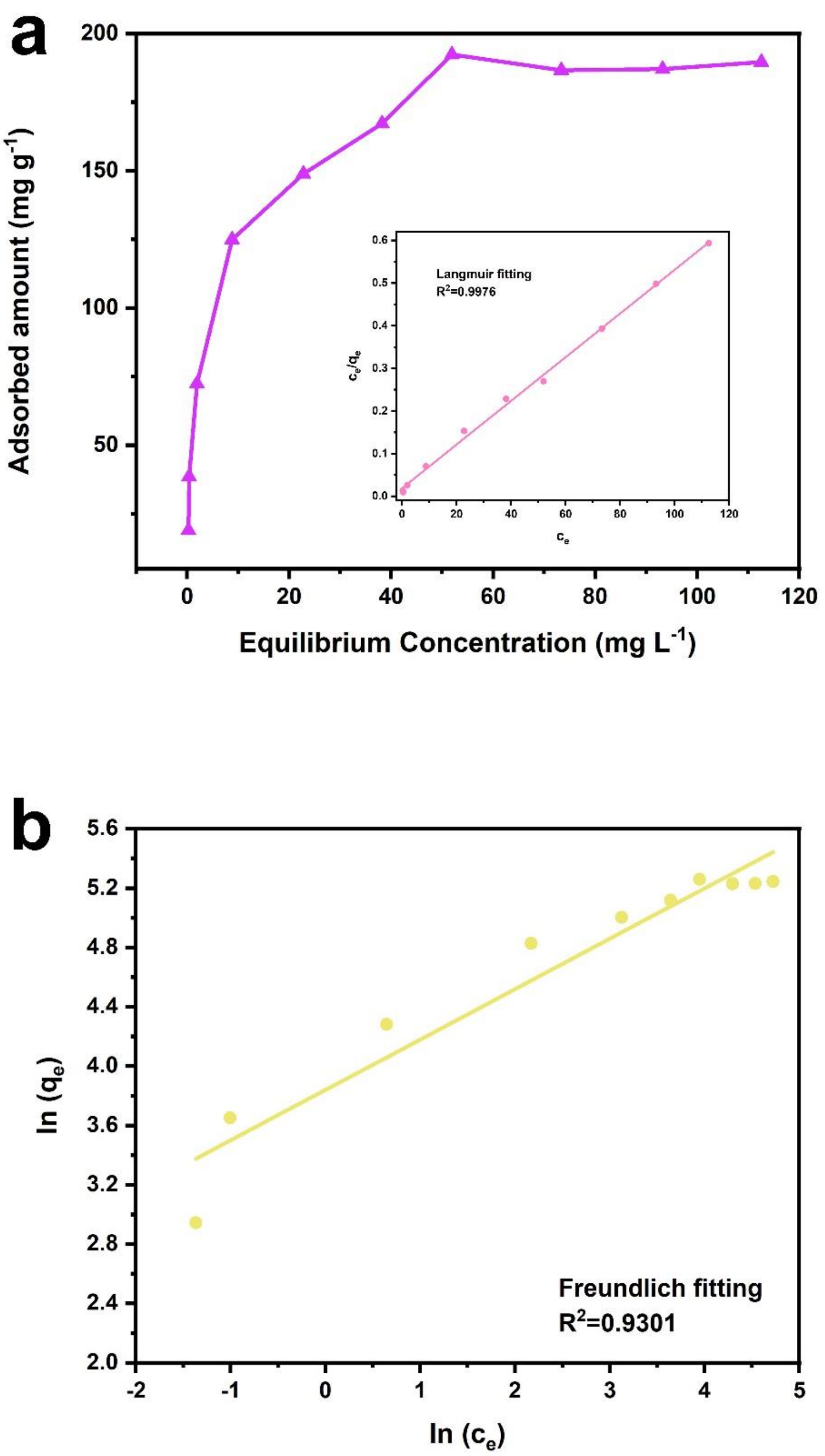

Figure S38. Relationship between equilibrium adsorption capacities and equilibrium concentrations, and the Langmuir isotherm model fitting curves (a) and the Freundlich isotherm model fitting curves (b) of PAF-525-9-25% toward TC.

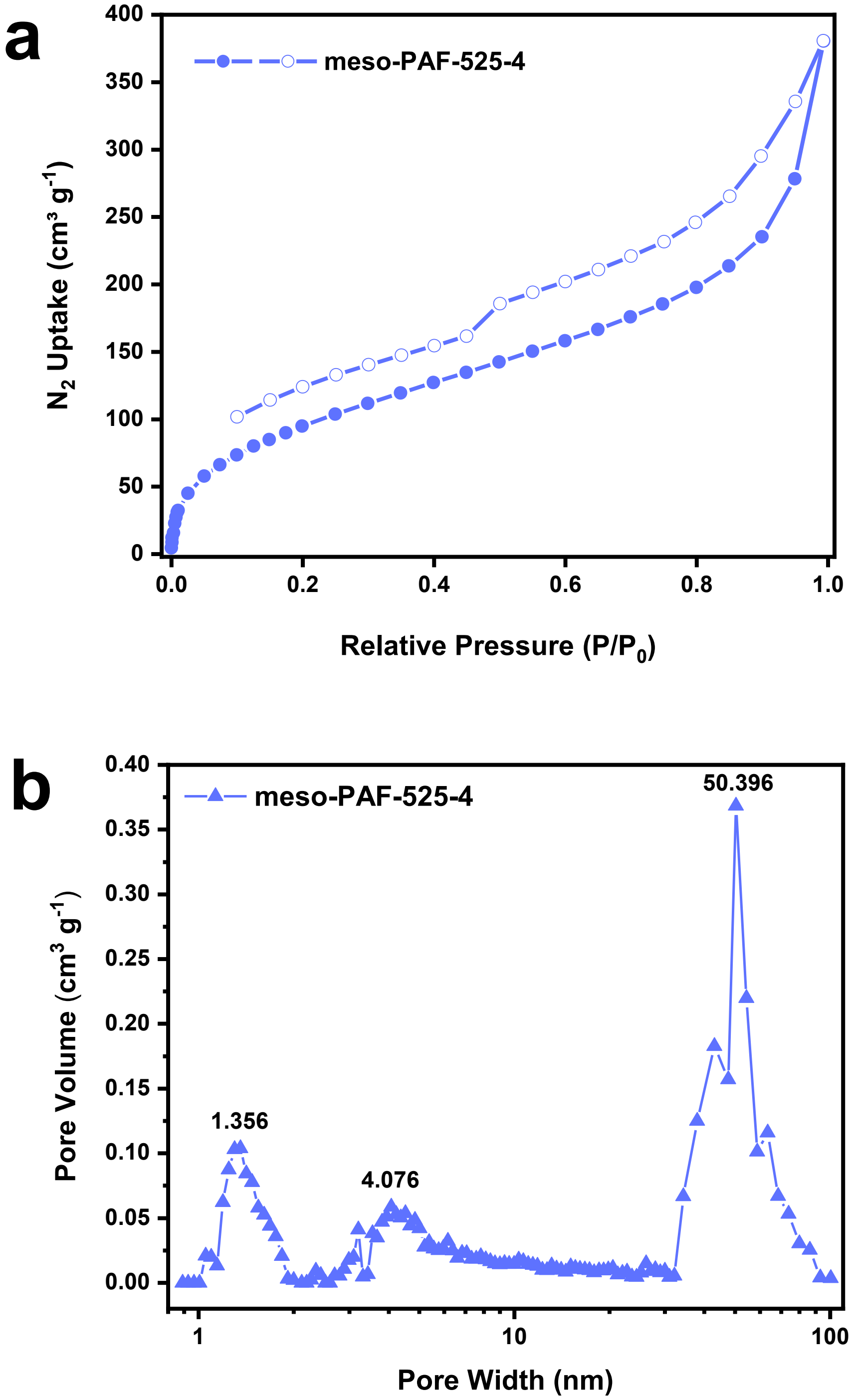
a
meso-PAF-525-4
N2 Uptake (cm3 g-1)
Relative Pressure (P/P0)
0
50
100
150
200
250
300
350
400
0.0
0.2
0.4
0.6
0.8
1.0
b
meso-PAF-525-4
Pore Volume (cm3 g-1)
Pore Width (nm)
1.356
4.076
50.396
0.00
0.05
0.10
0.15
0.20
0.25
0.30
0.35
0.40
1
10
100

c

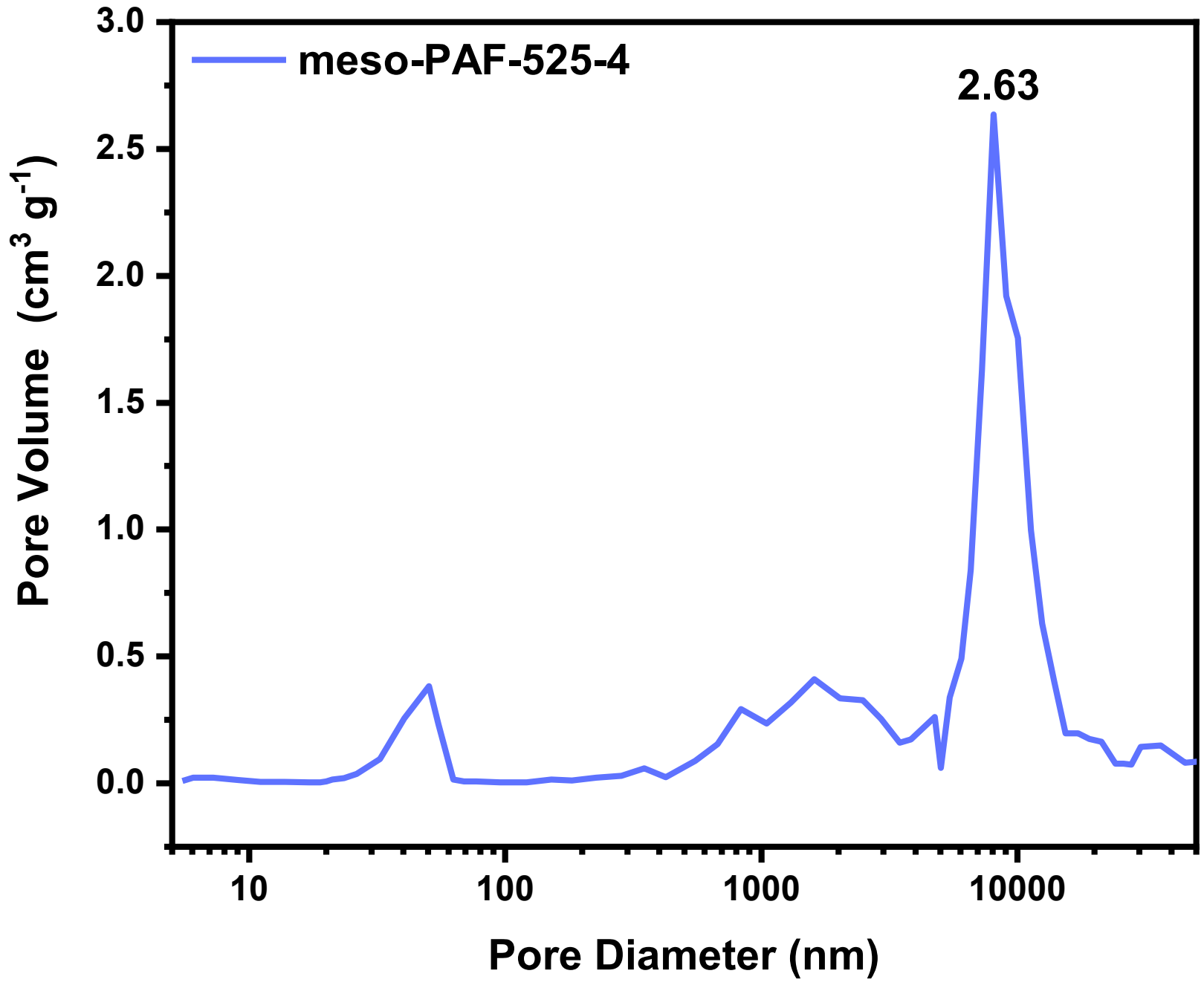

Figure S39. (a) N_2 adsorption and desorption isotherms of meso-PAF-525-4 at 77.3 K and (b) Pore size distributions of meso-PAF-525-4 calculated by using density functional theory (DFT) methods. (c) Pore size distributions of meso-PAF-525-4 calculated by mercury intrusion porosimetry.

a

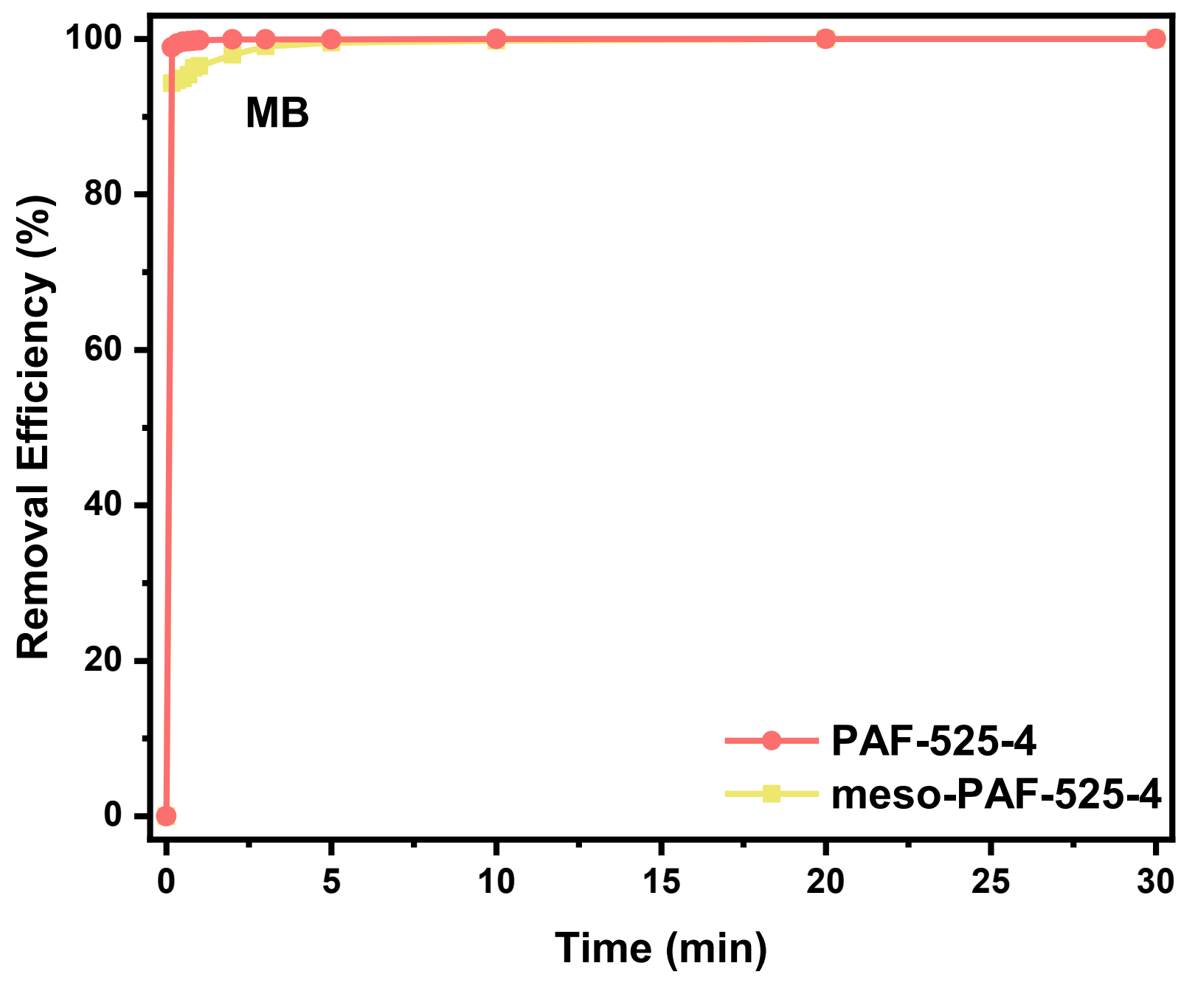

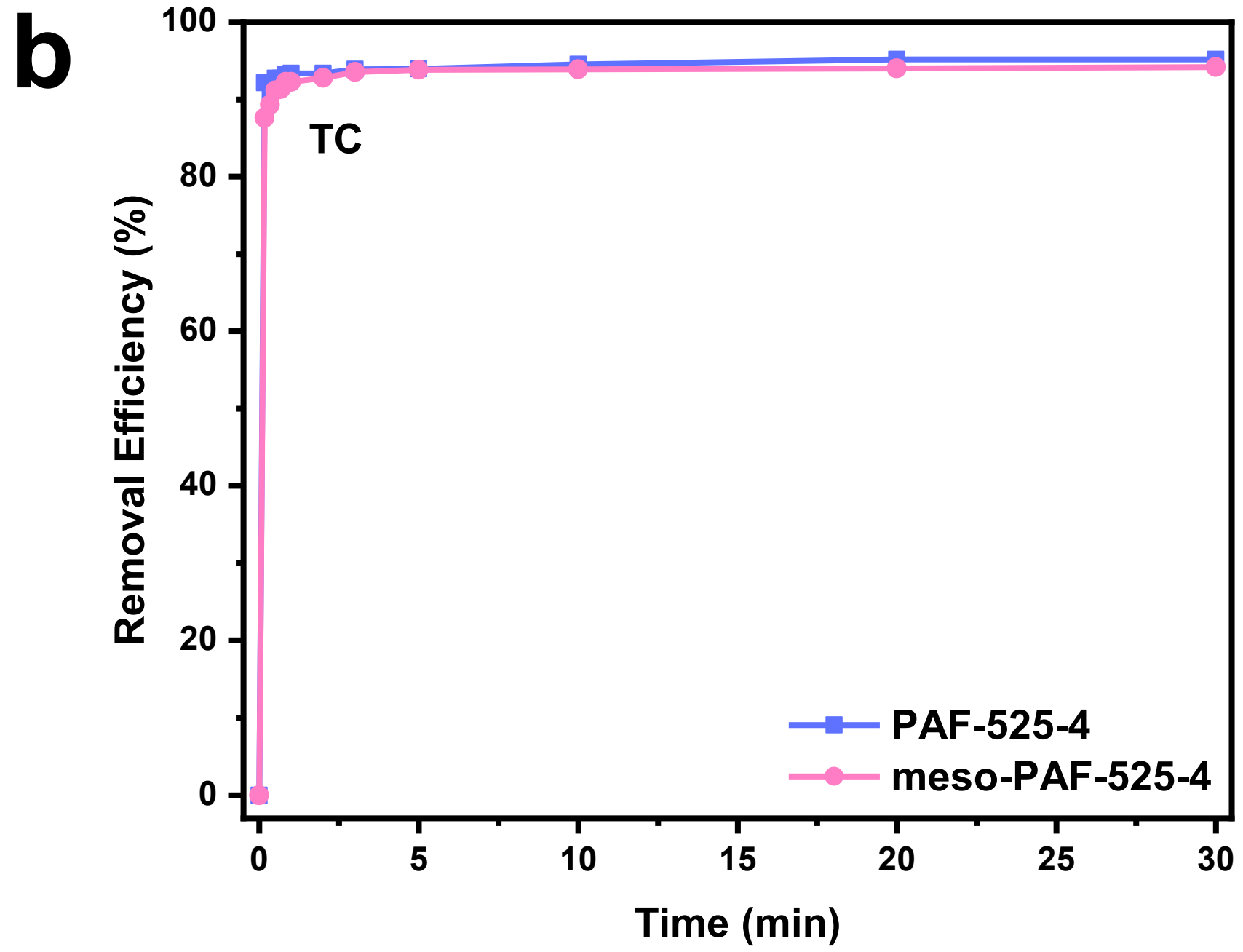

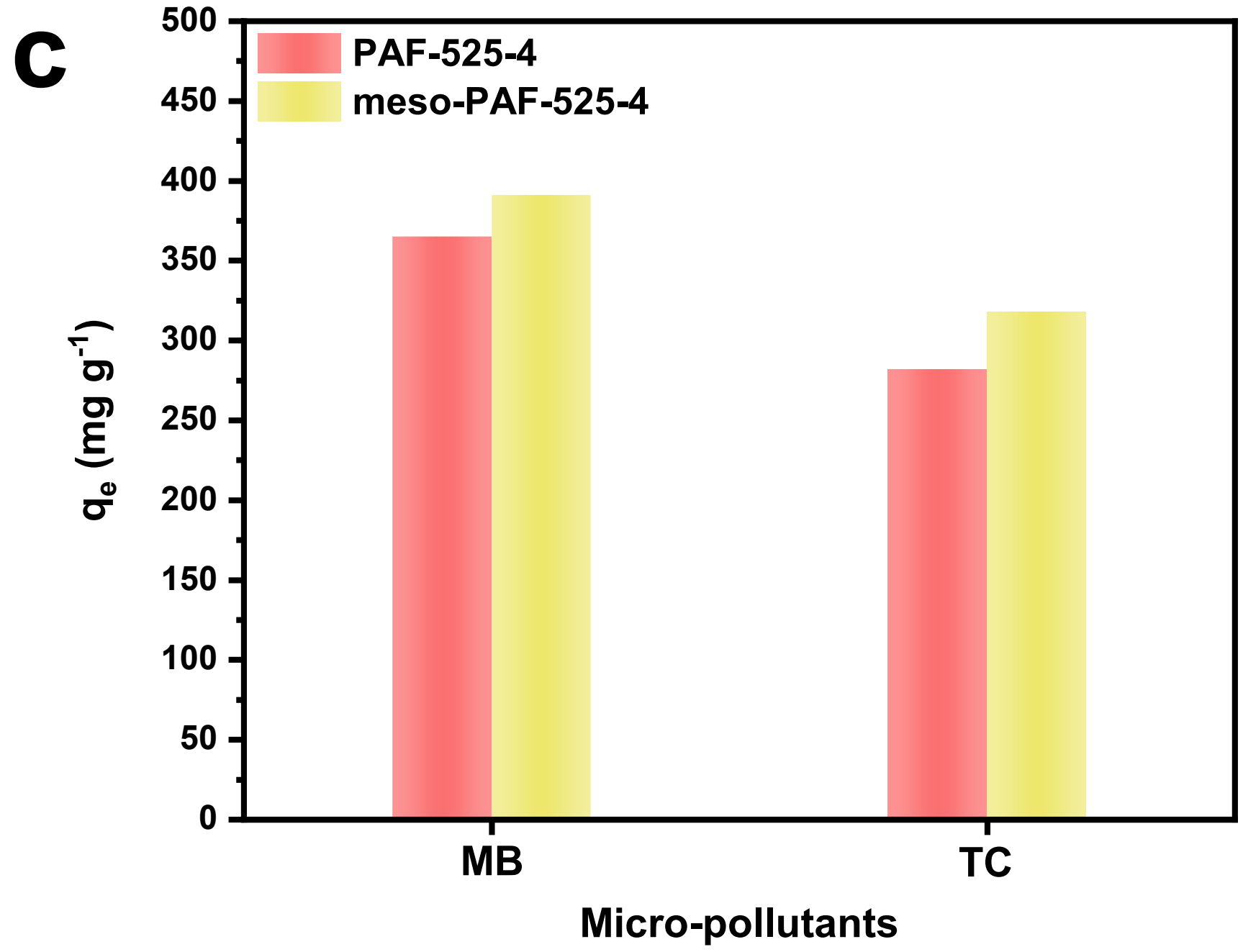

Figure S40. Removal efficiency of meso-PAF-525-4 and PAF-525-4 for MB (a) and TC (b). Maximum adsorption capacity of of meso-PAF-525-4 and PAF-525-4 for MB and TC (c).

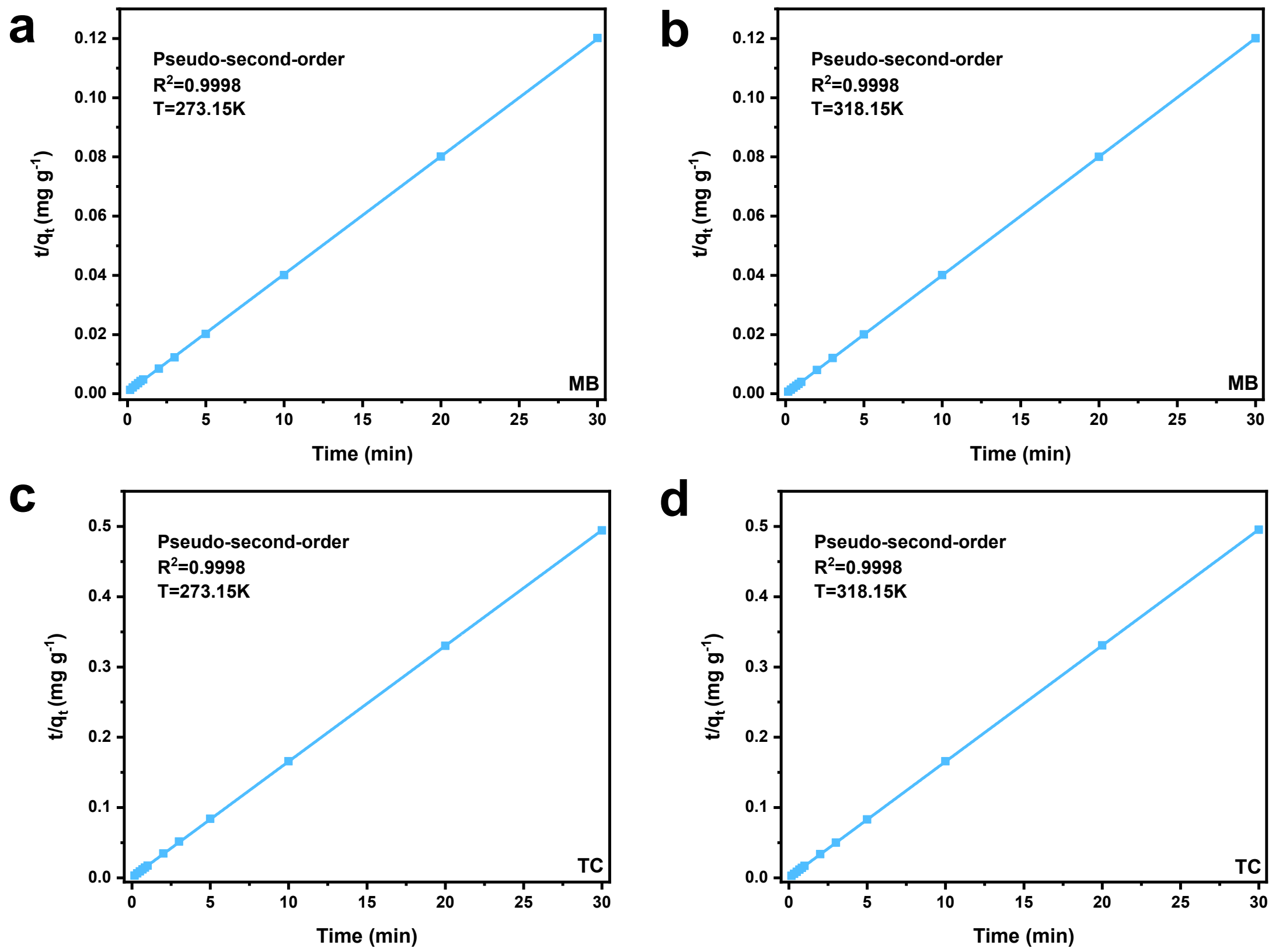

Figure S41. The pseudo-second-order kinetic data of PAF-525-9 for MB at 237.15 K (a) and 318.15 K (b). The pseudo-second-order kinetic data of PAF-525-9 for TC at 237.15 K (c) and 318.15 K (d).

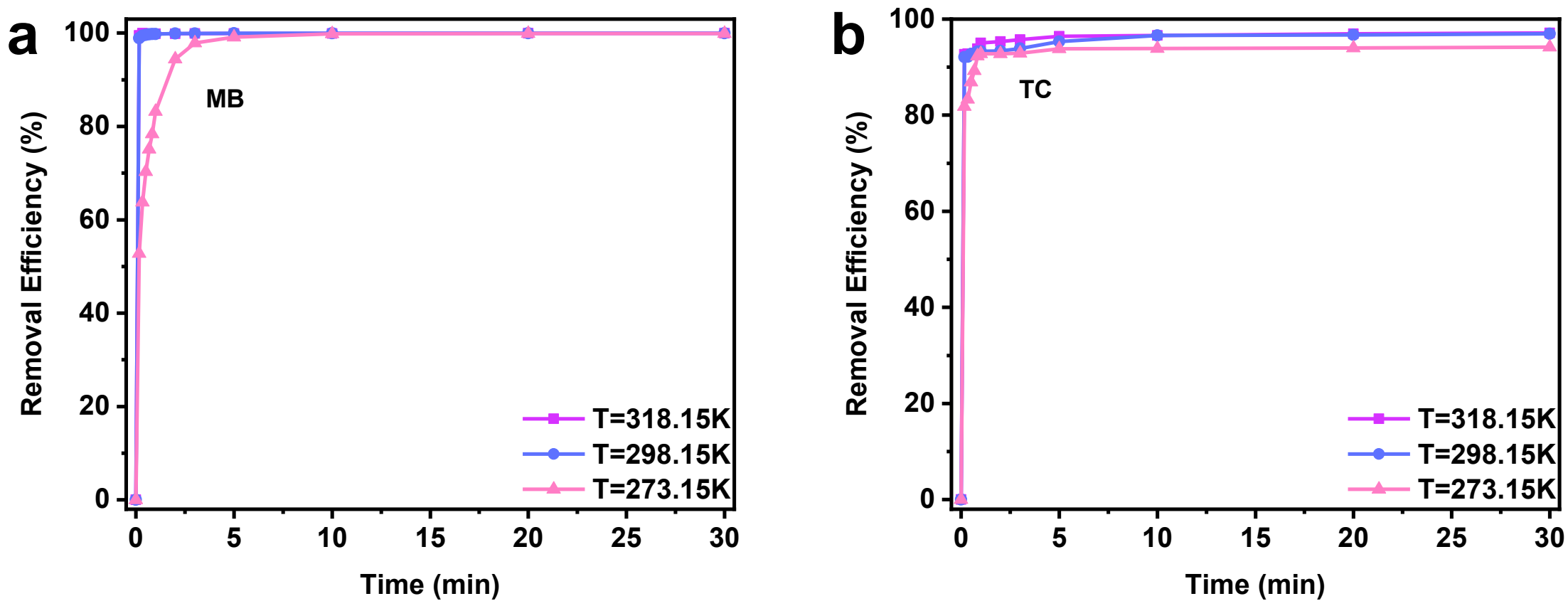

Figure S42. Removal efficiency of PAF-525-9 for MB (a) and TC (b) at different temperatures.

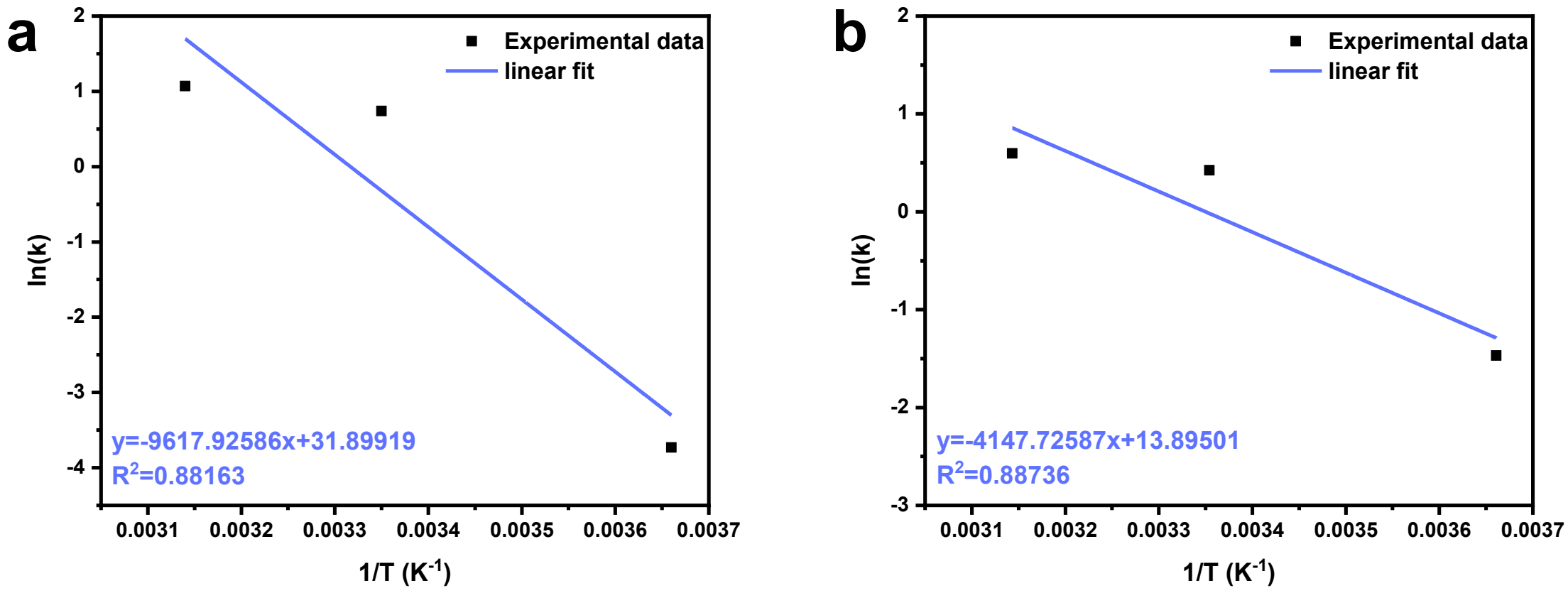

Figure S43. Arrhenius plot established based on experimental data obtained for MB (a) and TC (b) adsorption on the surface of PAF-525-9 at different temperatures.

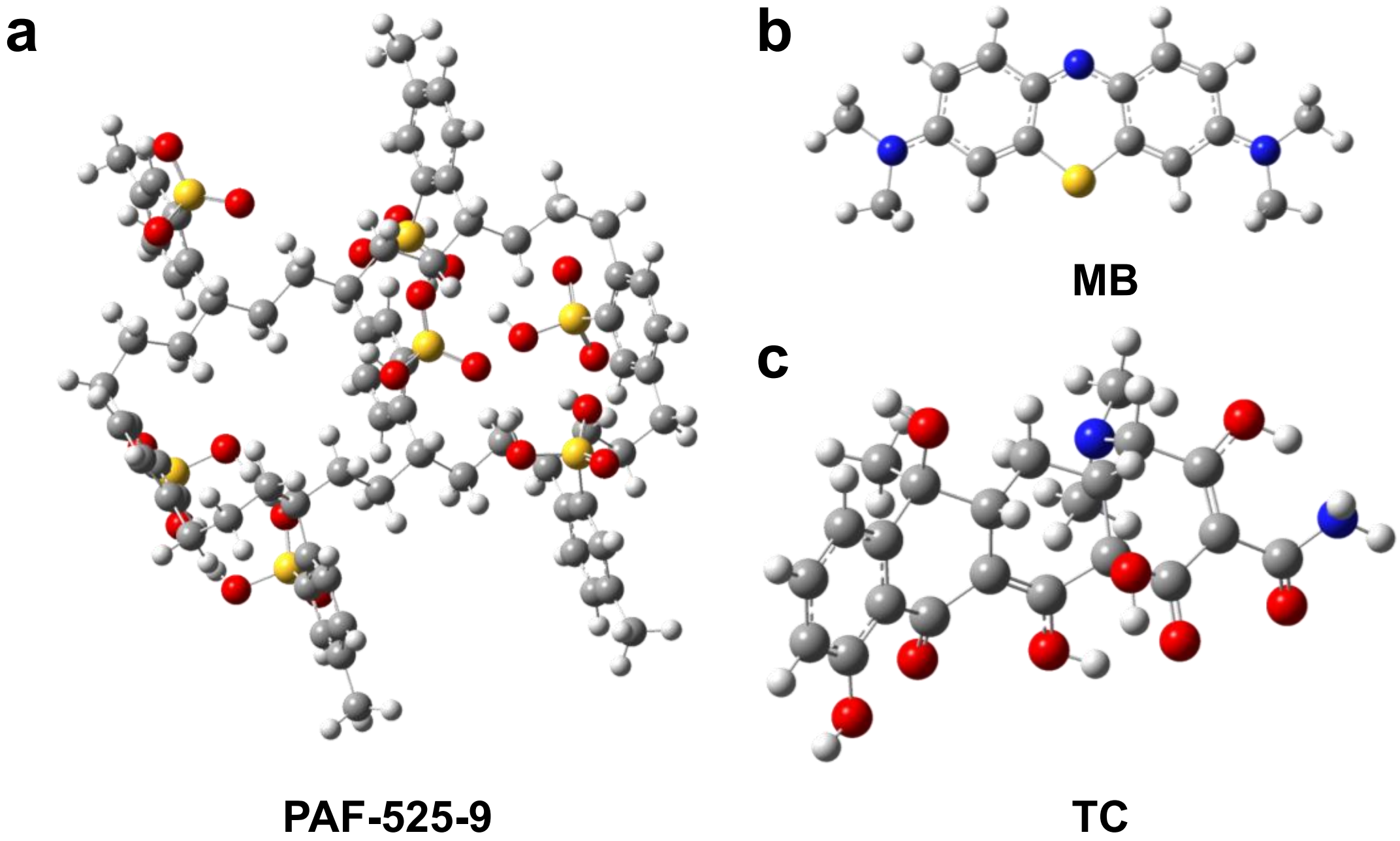

Figure S44. Structural models of PAF-525-9 fragment (a), MB (b) and TC (c).

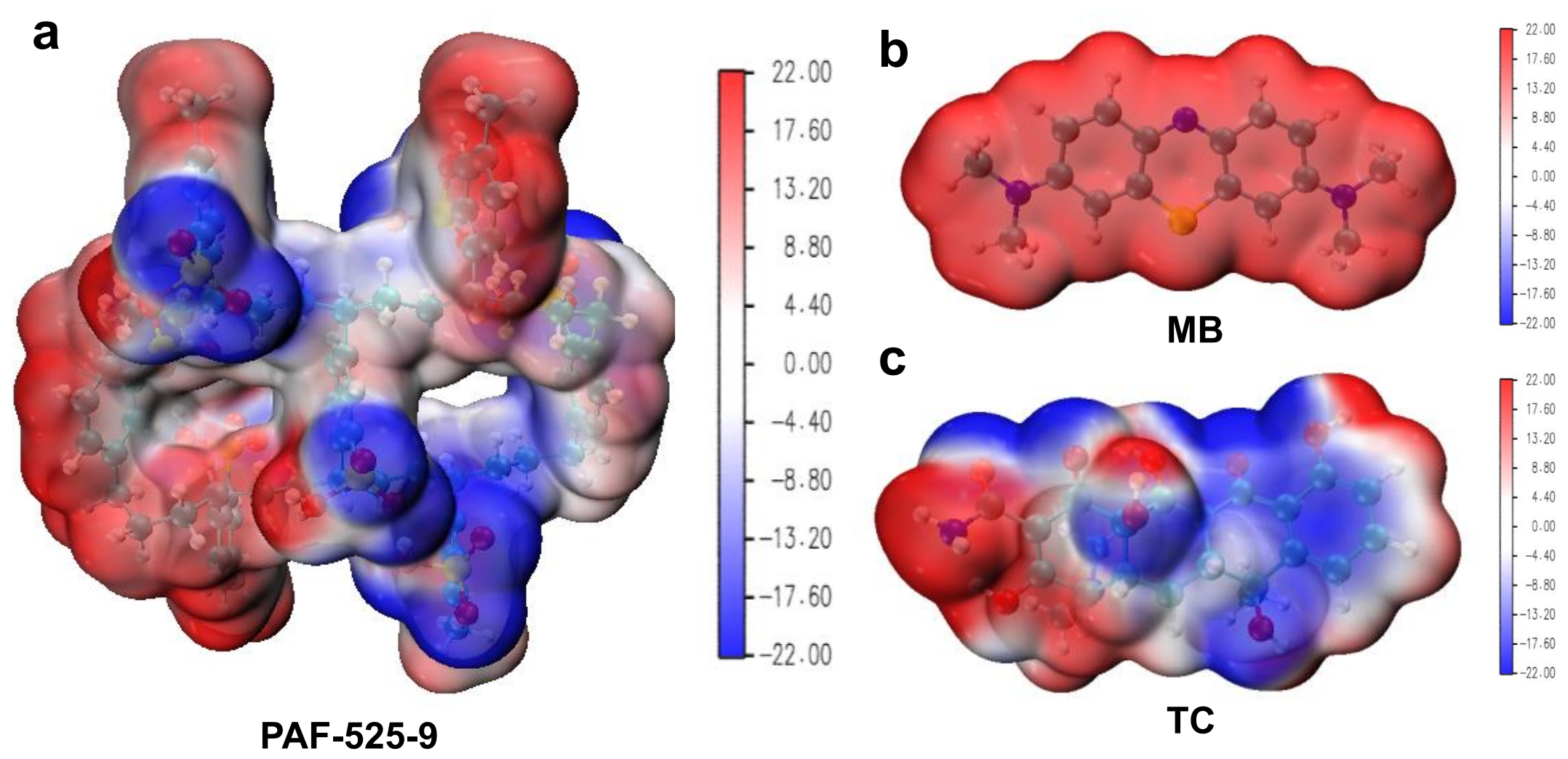

Figure S45. ESP images of PAF-525-9 fragment (a), MB (b) and TC (c).

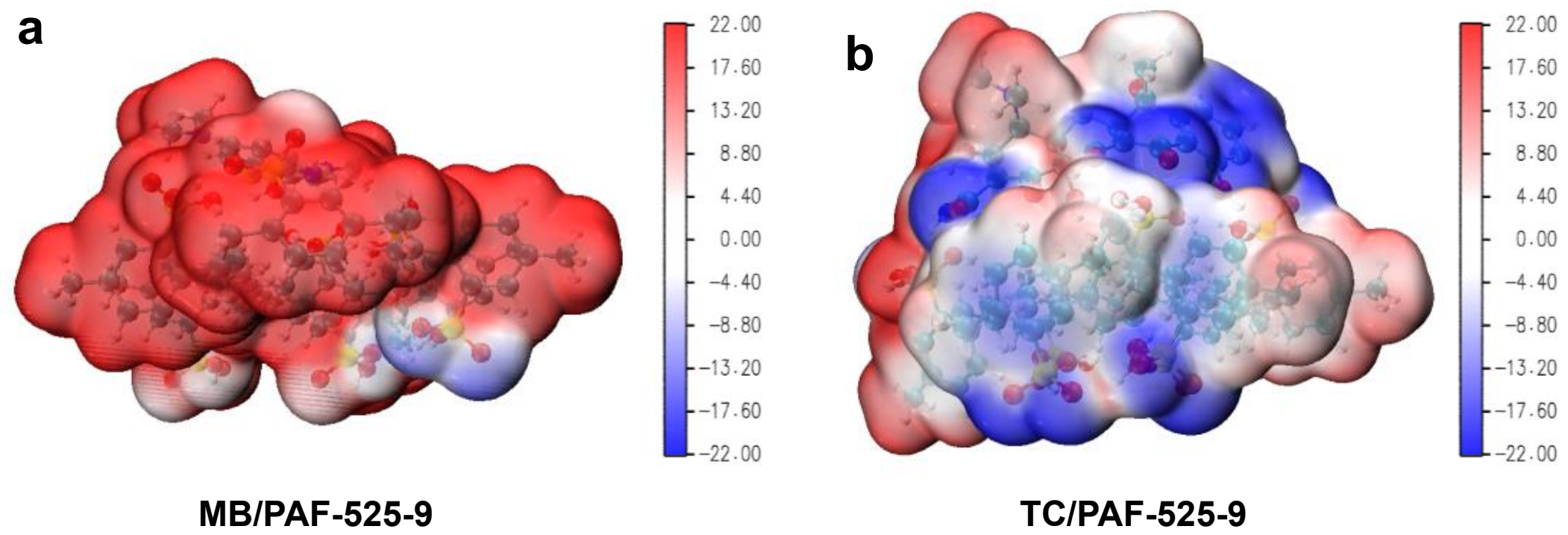

Figure S46. (a) ESP images of MB/PAF-525-9 and TC/PAF-525-9.

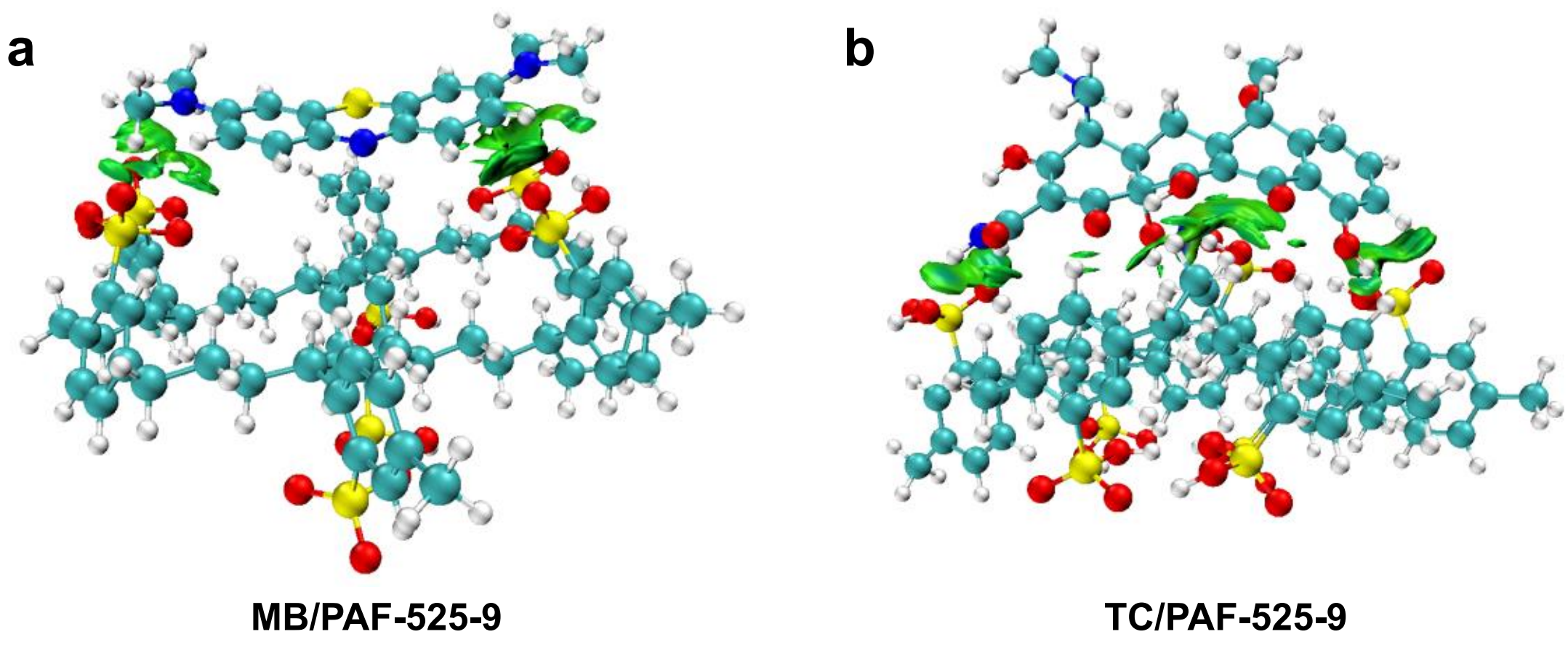

Figure S47. (a) Schematic diagram of intermolecular interactions between PAF-525-9 and MB. (b) Schematic diagram of intermolecular interactions between PAF-525-9 and TC.

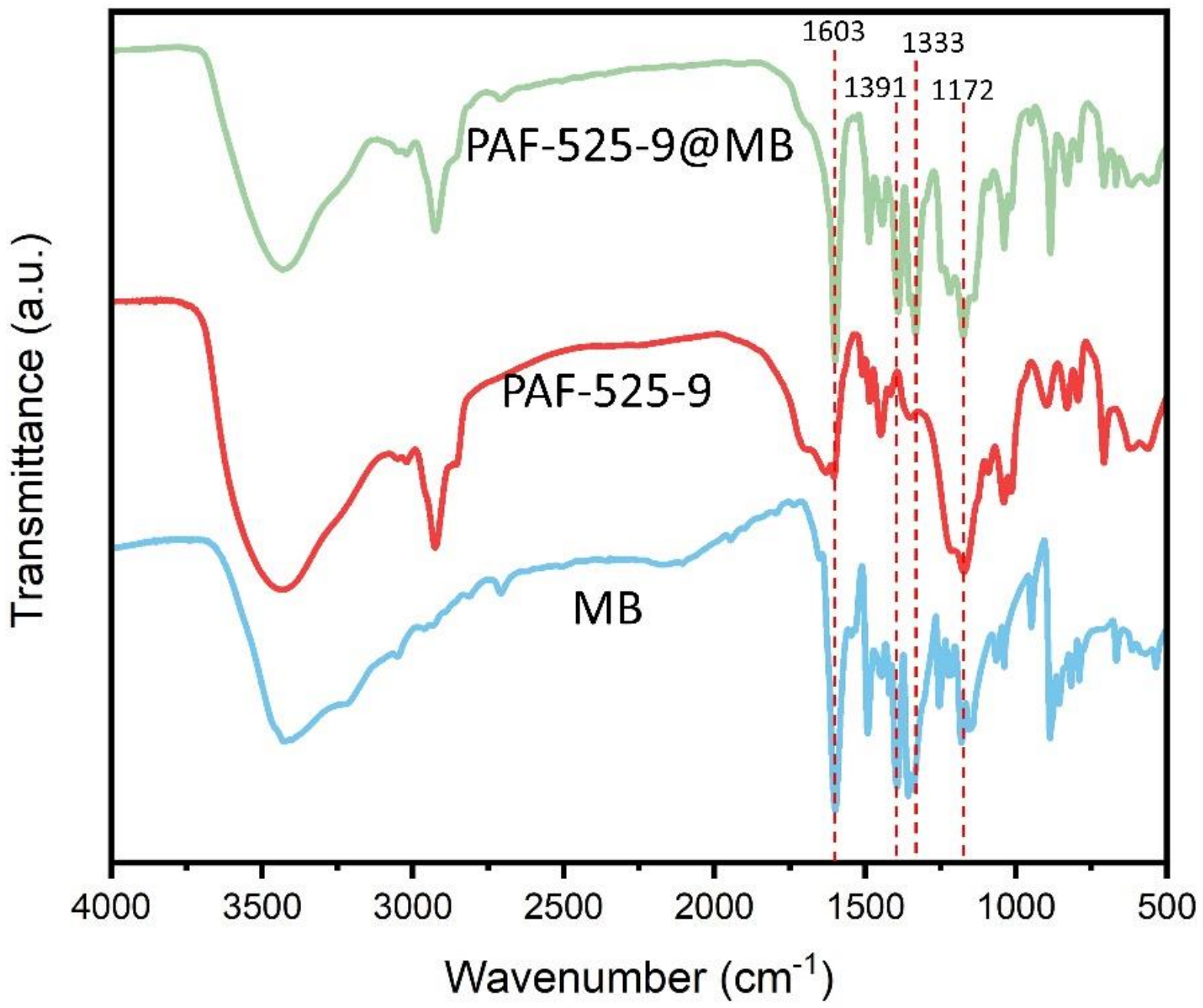

Figure S48. FT-IR spectra of MB, PAF-525-9 and PAF-525-9@MB.

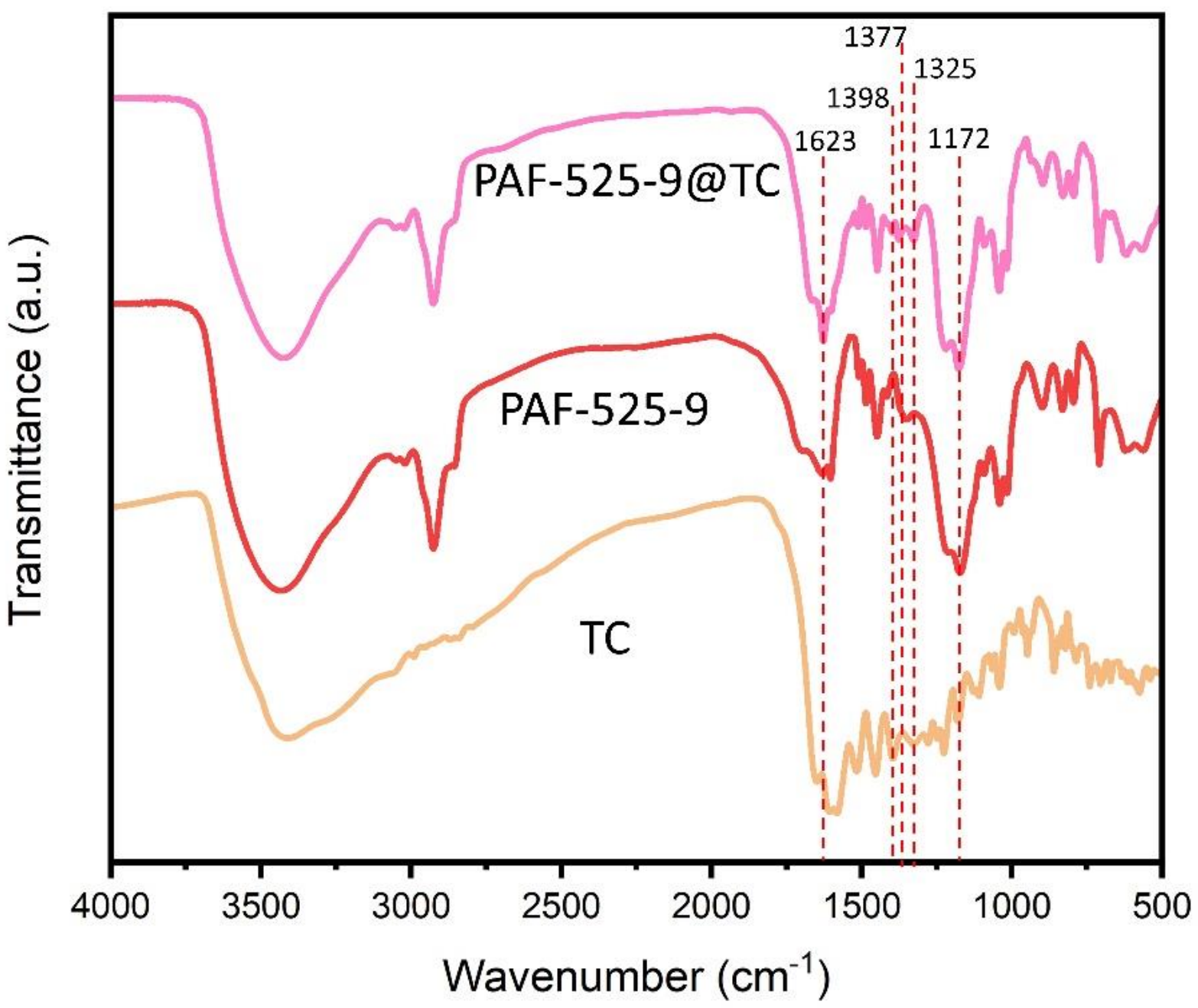

Figure S49. FT-IR spectra of TC, PAF-525-9 and PAF-525-9@TC.

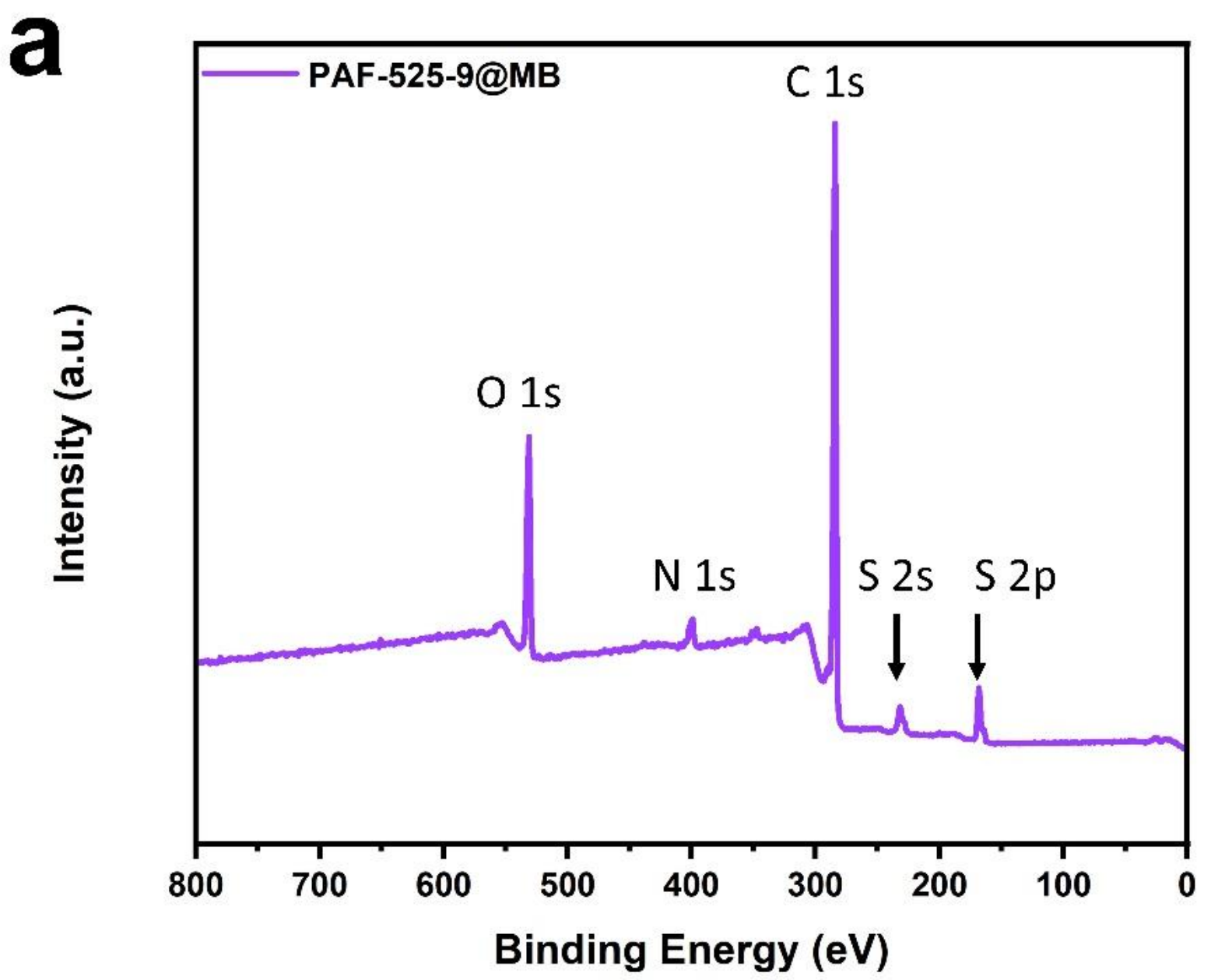
a
PAF-525-9@MB
C 1s
O 1s
N 1s
S 2s
S 2p
Intensity (a.u.)
800
700
600
500
400
300
200
100
0
Binding Energy (eV)

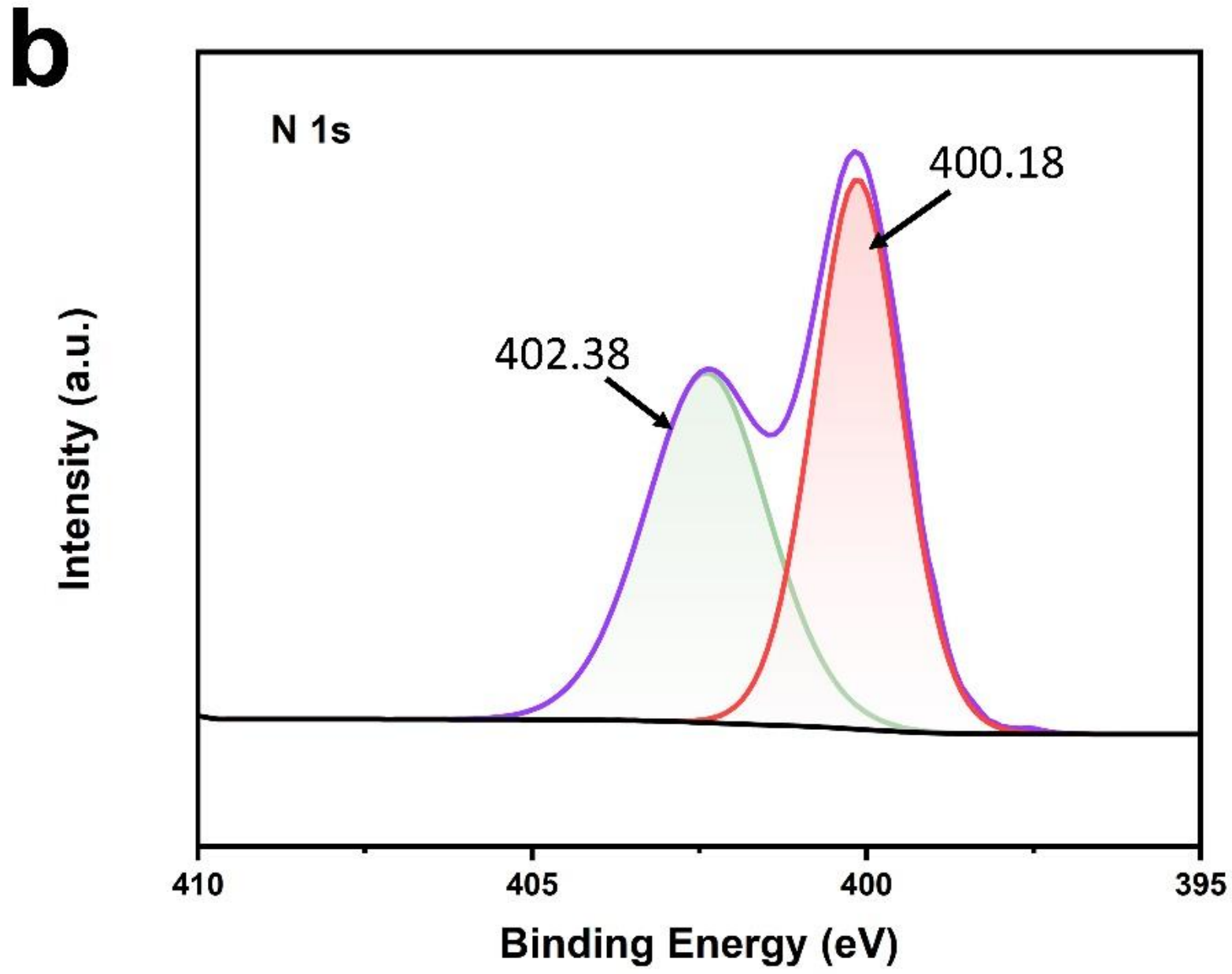
b
N 1s
400.18
402.38
Intensity (a.u.)
410
405
400
395
Binding Energy (eV)

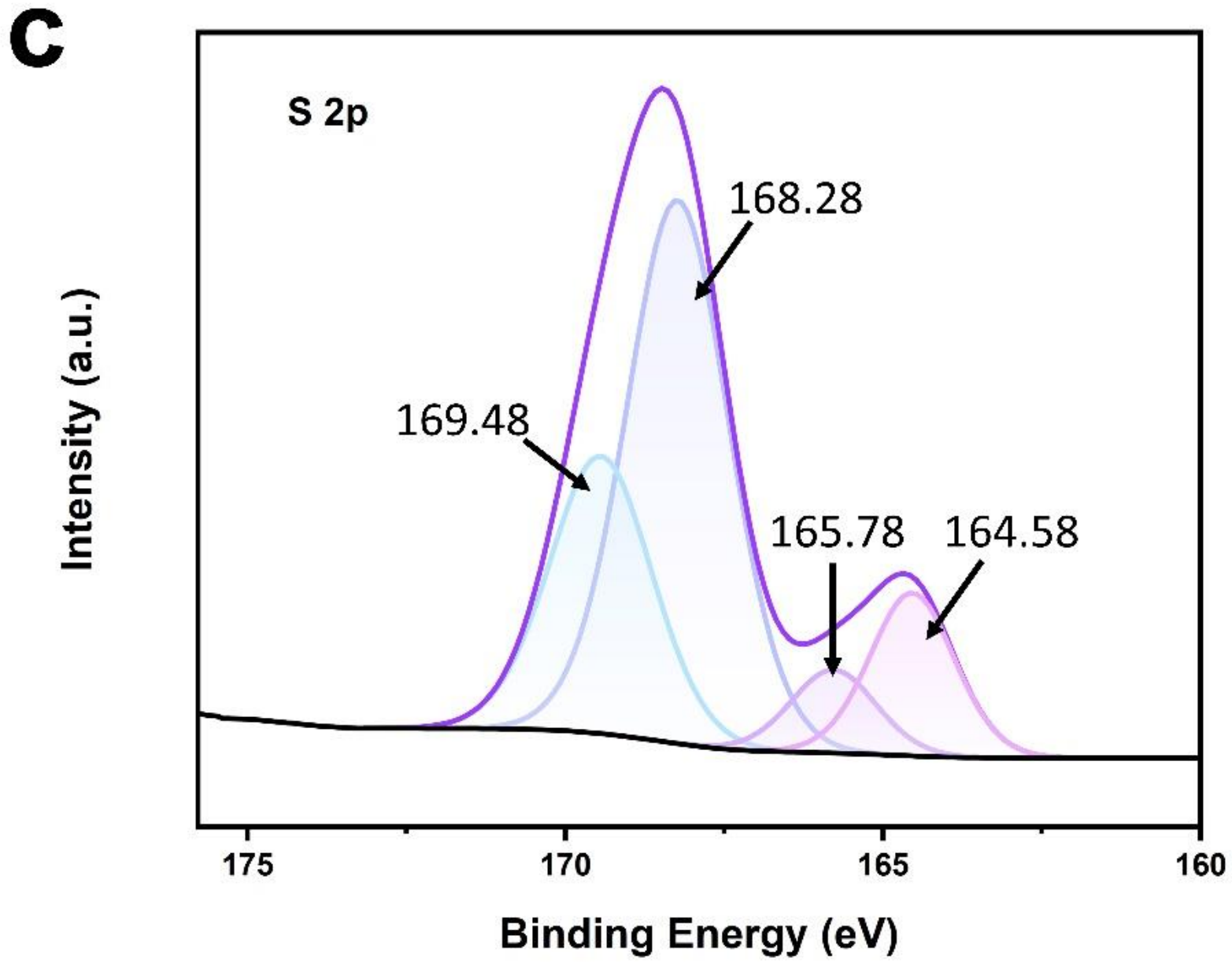

Figure S50. (a) XPS spectrum, (b) N 1s and (c) S 2p high resolution XPS spectra of PAF-525-9@MB.

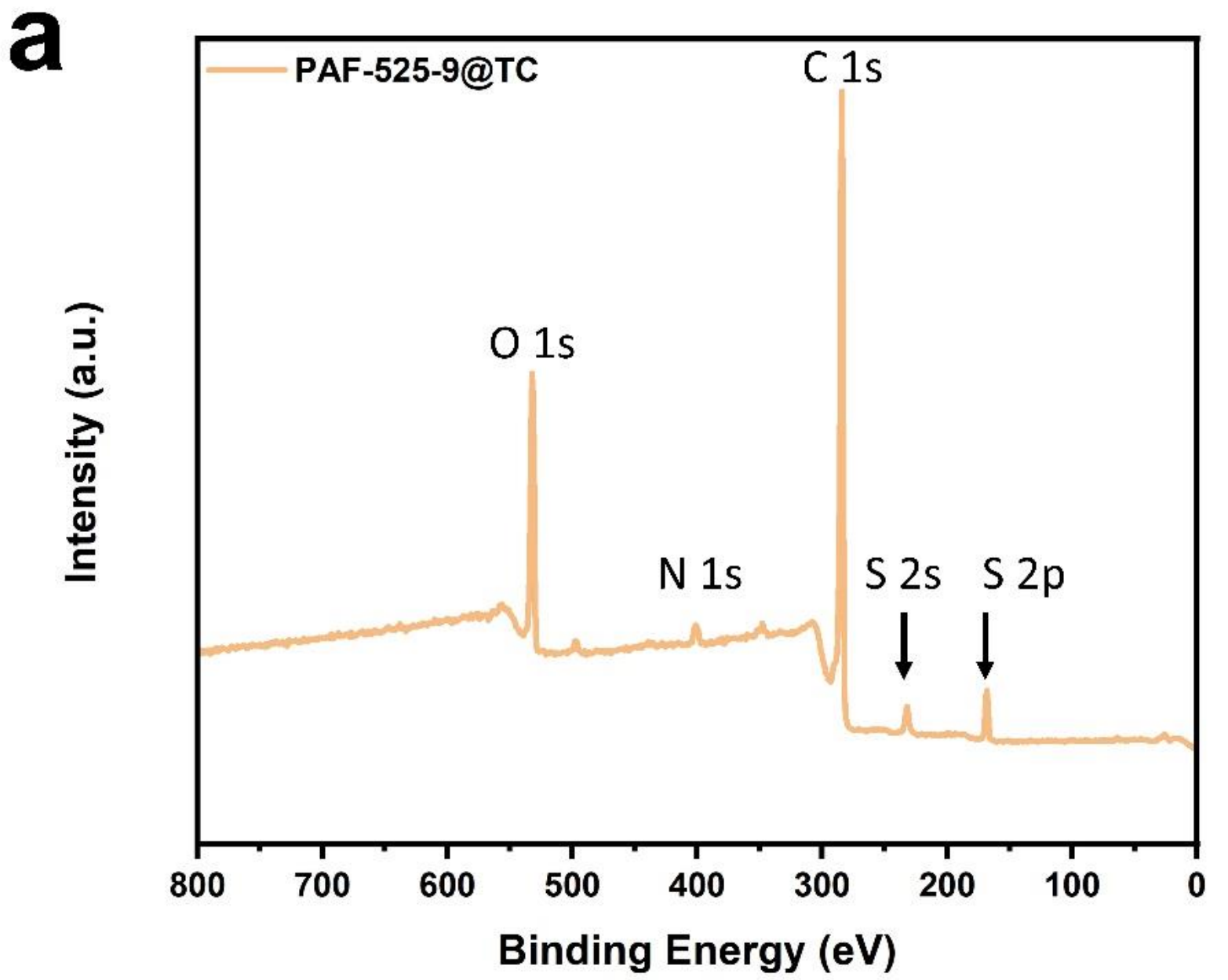

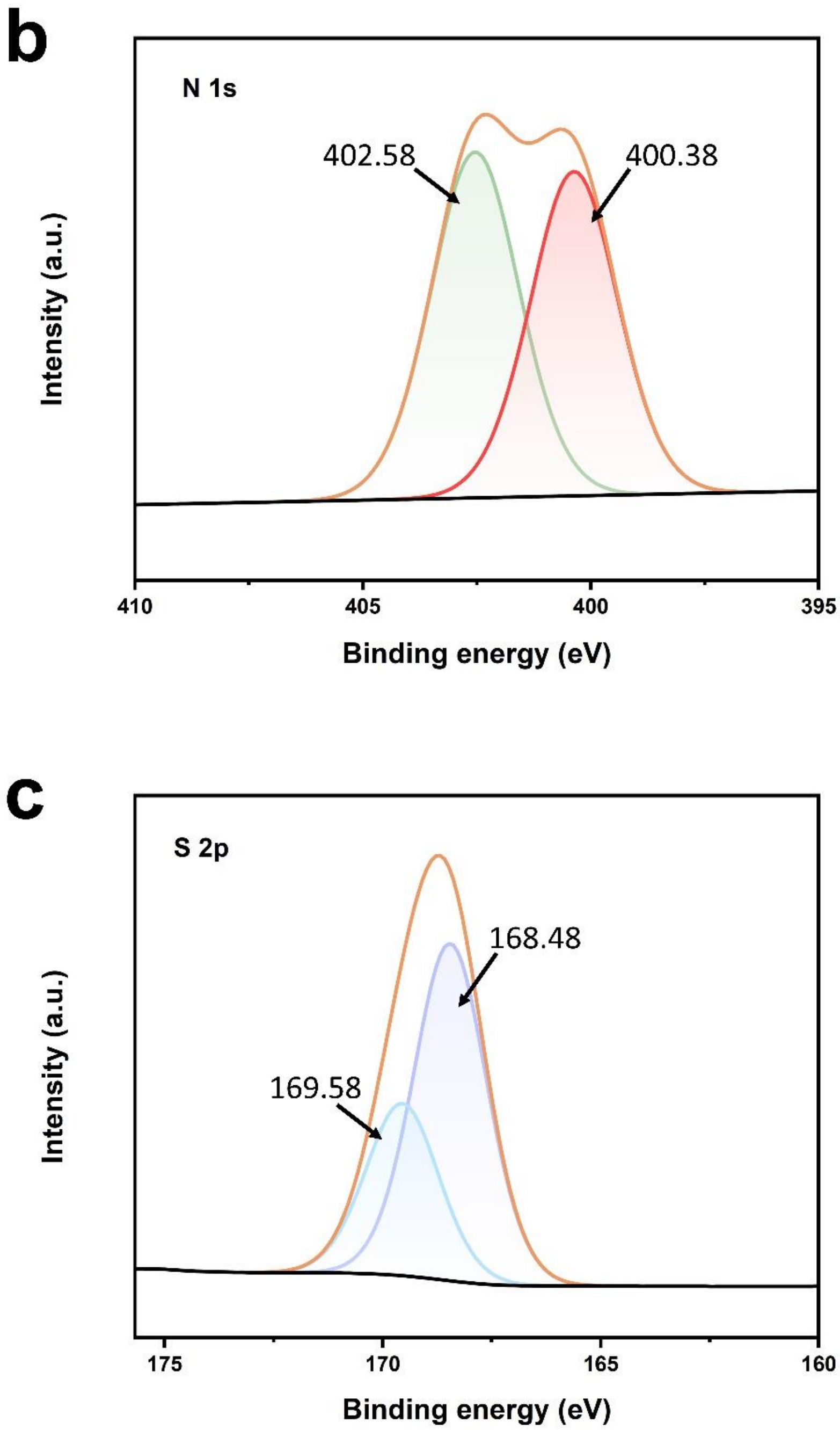

Figure S51. (a) XPS spectrum, (b) N 1s and (c) S 2p high resolution XPS spectra of PAF-525-9@TC.

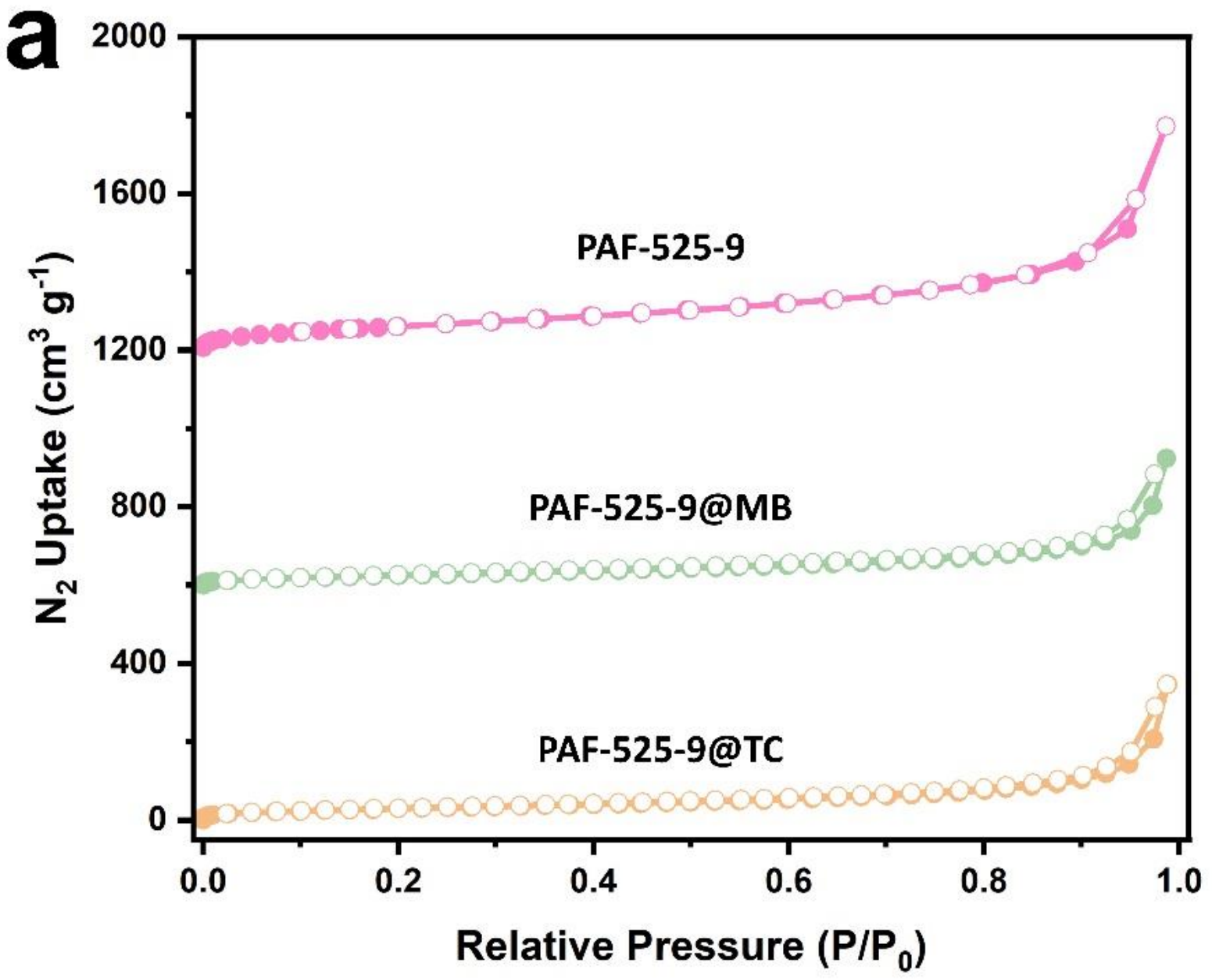

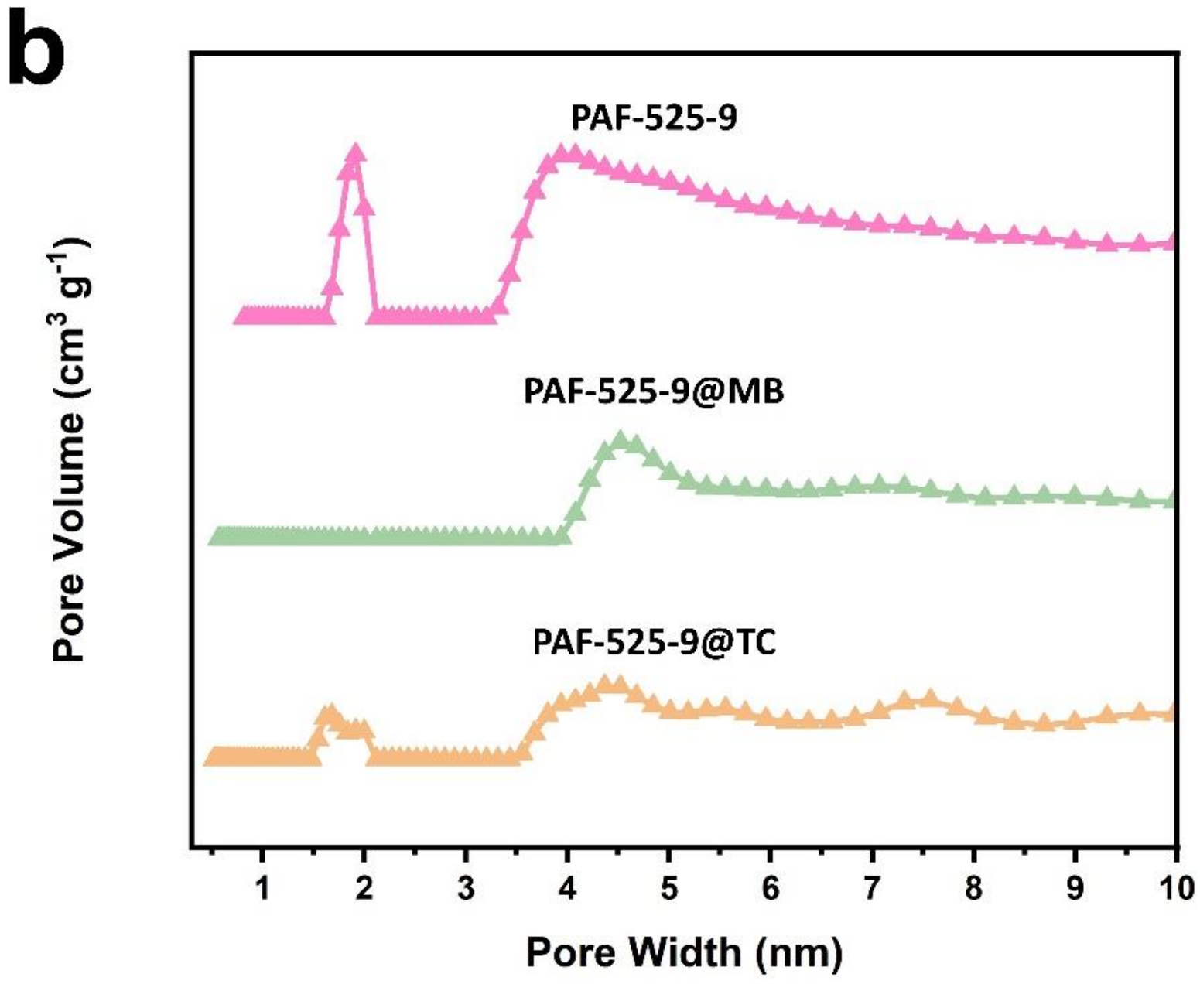

Figure S52. (a) N_2 adsorption and desorption isotherms of PAF-525-9, PAF-525-9@MB and PAF-525-9@TC at 77.3 K and (b) Pore size distributions of PAF-525-9, PAF-525-9@MB and PAF-525-9@TC calculated by using density functional theory (DFT) methods (slit pore models and differential pore volumes).

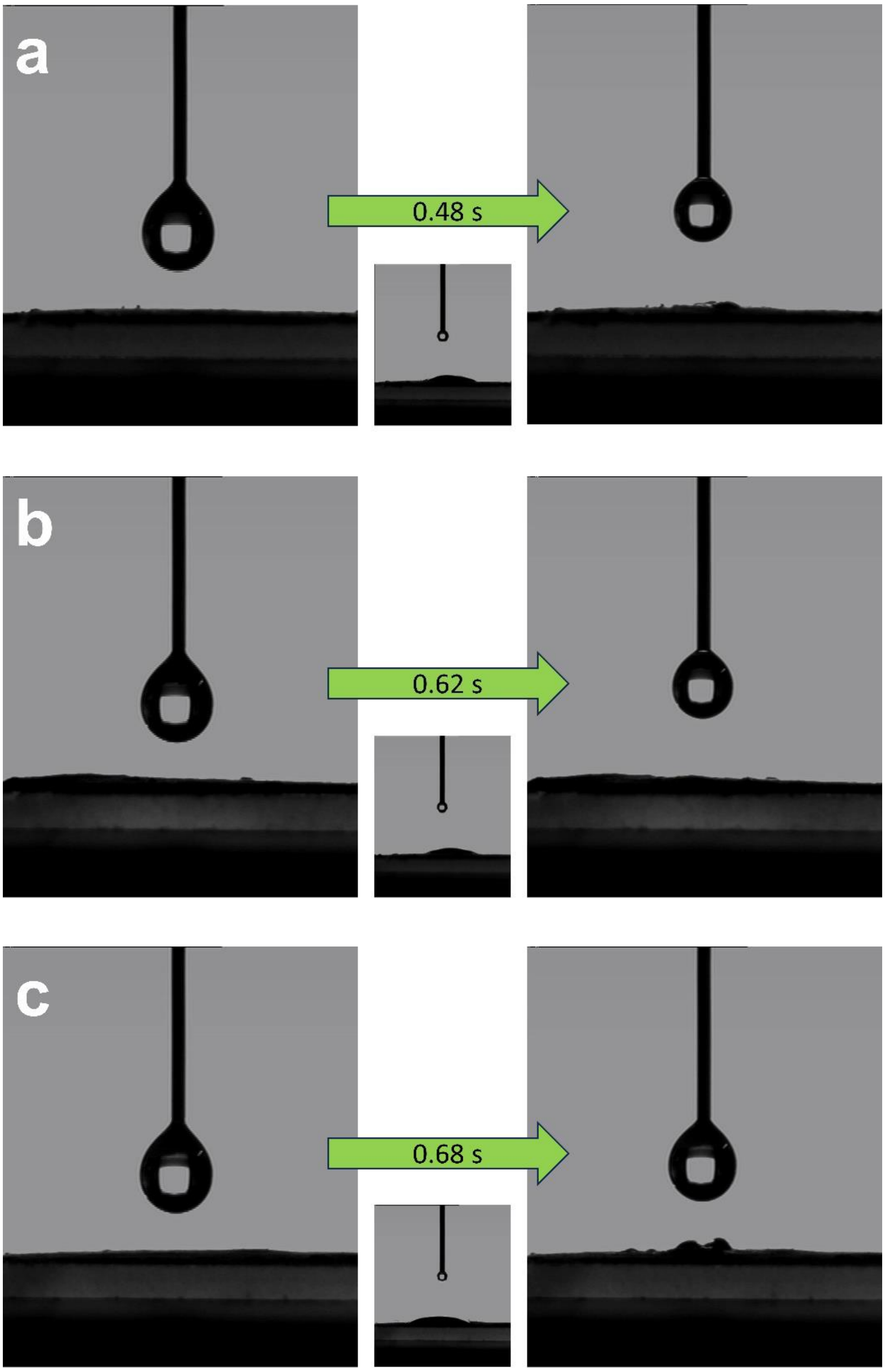

Figure S53. Water contact angle of PAF-525-9 (a), PAF-525-9@MB (b) and PAF-525-9@TC (c).

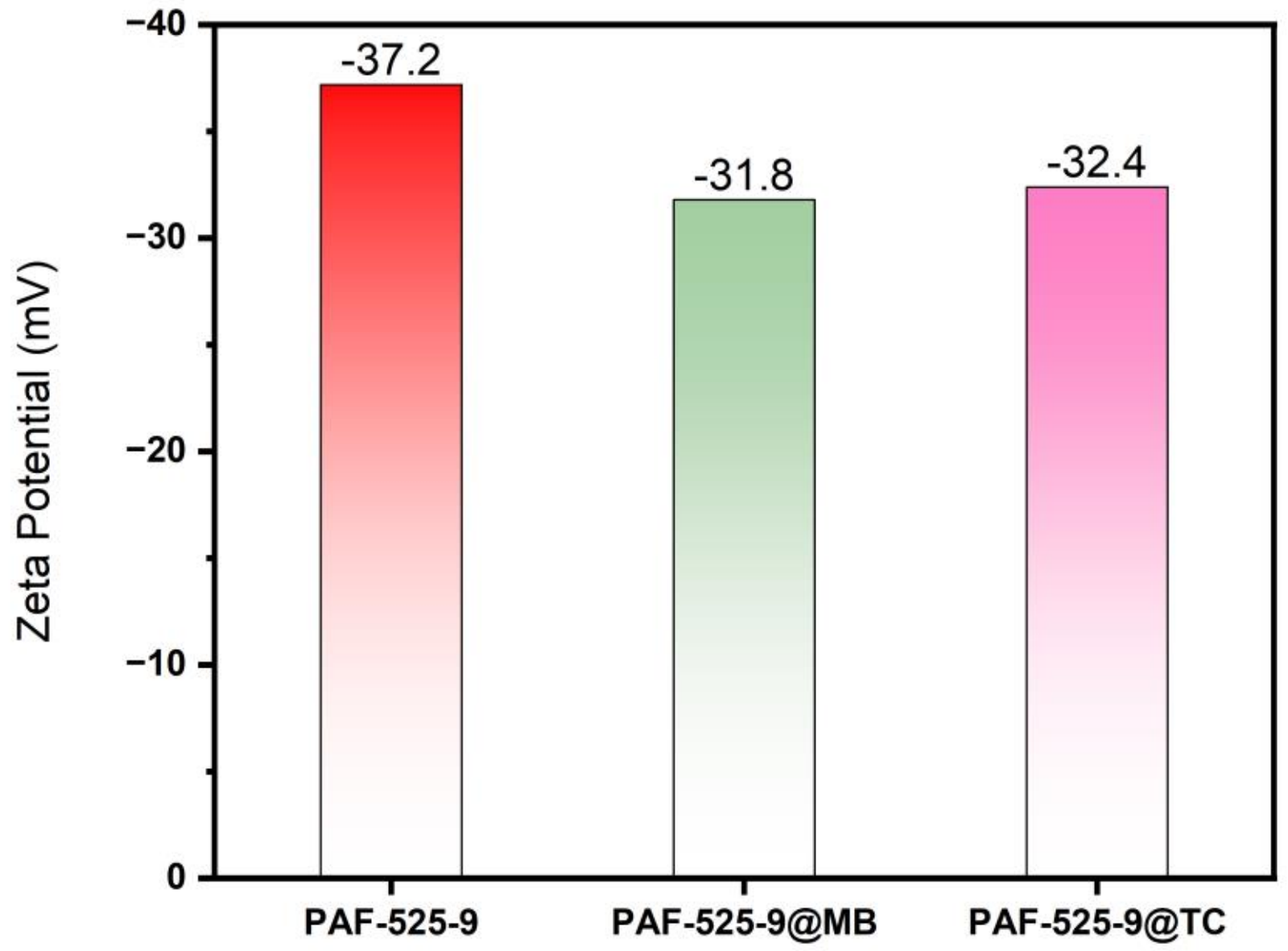

Figure S54. Zeta potentials of PAF-525-9, PAF-525-9@MB and PAF-525-9@TC.

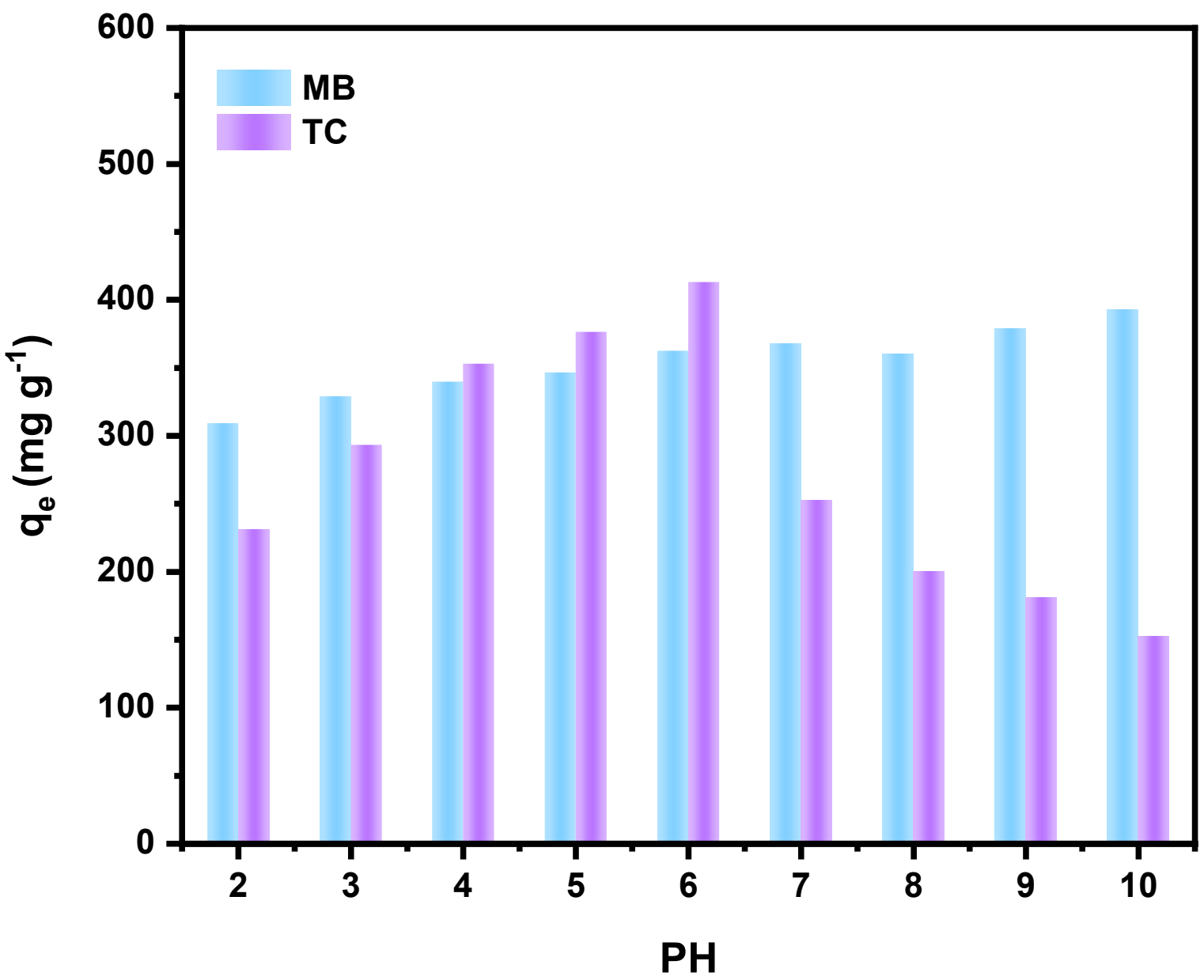

Figure S55. Maximum adsorption capacity of MB and TC at different pH value for PAF-525-9.

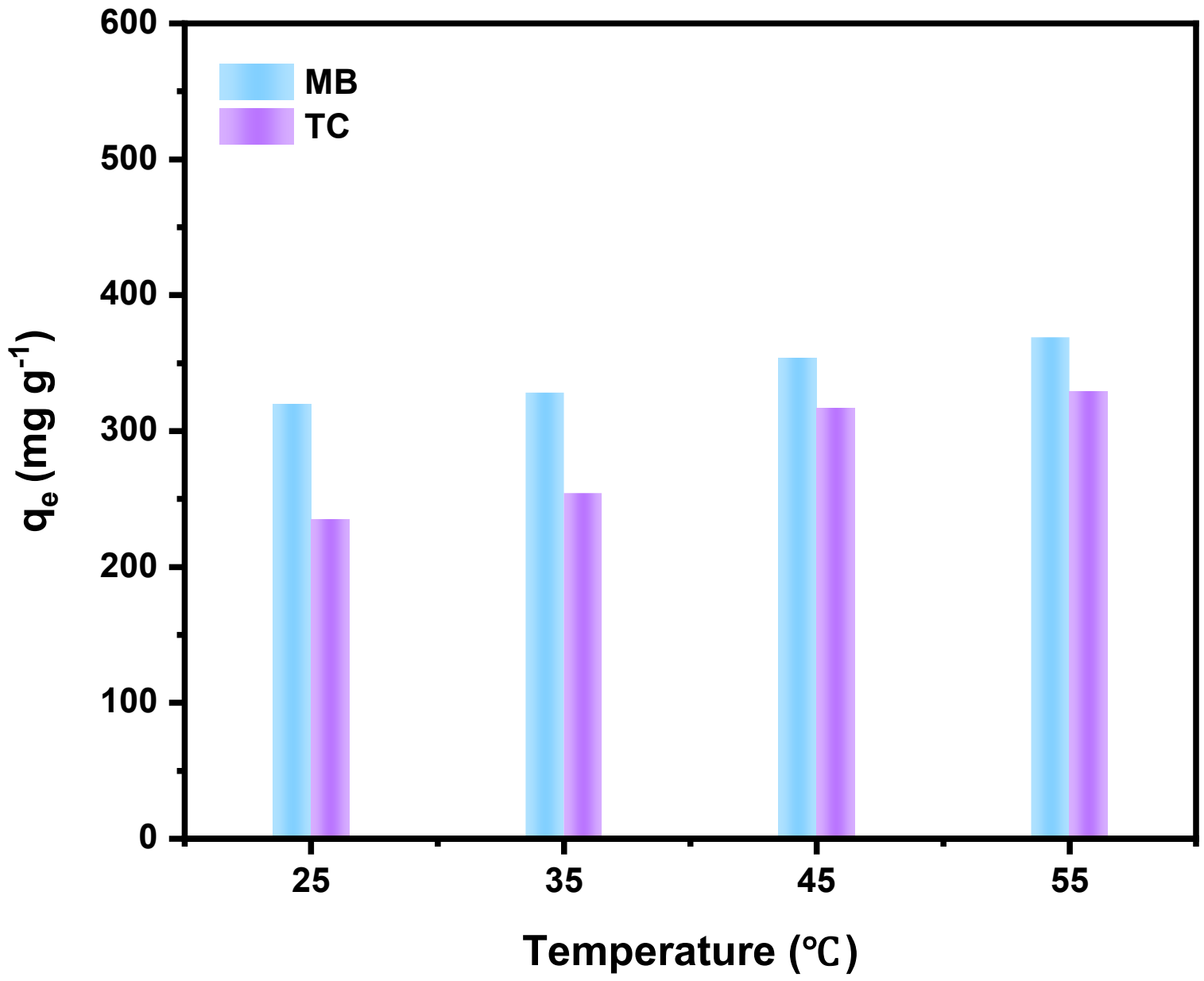

Figure S56. Maximum adsorption capacity of MB and TC at different temperature for PAF-525-9.

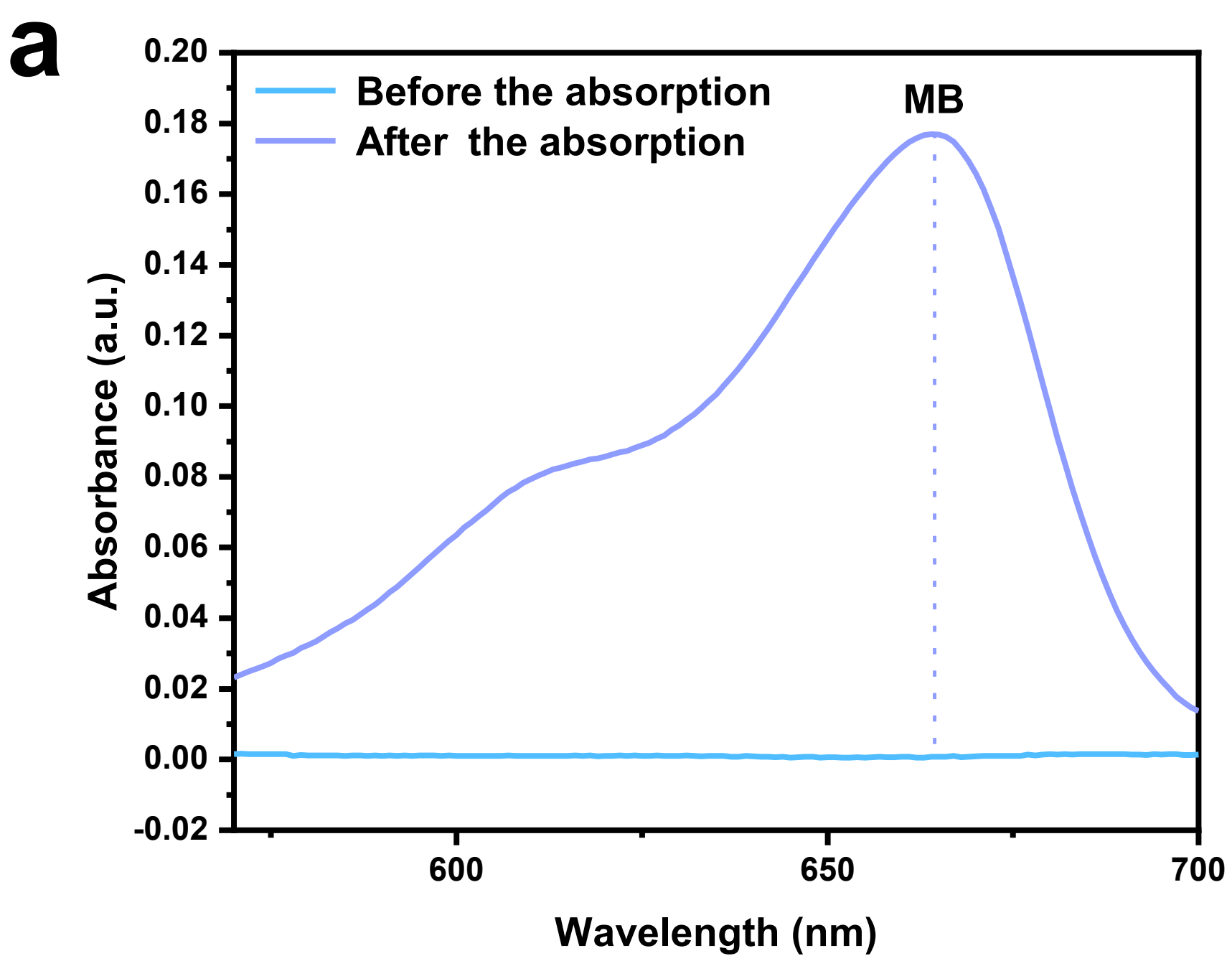

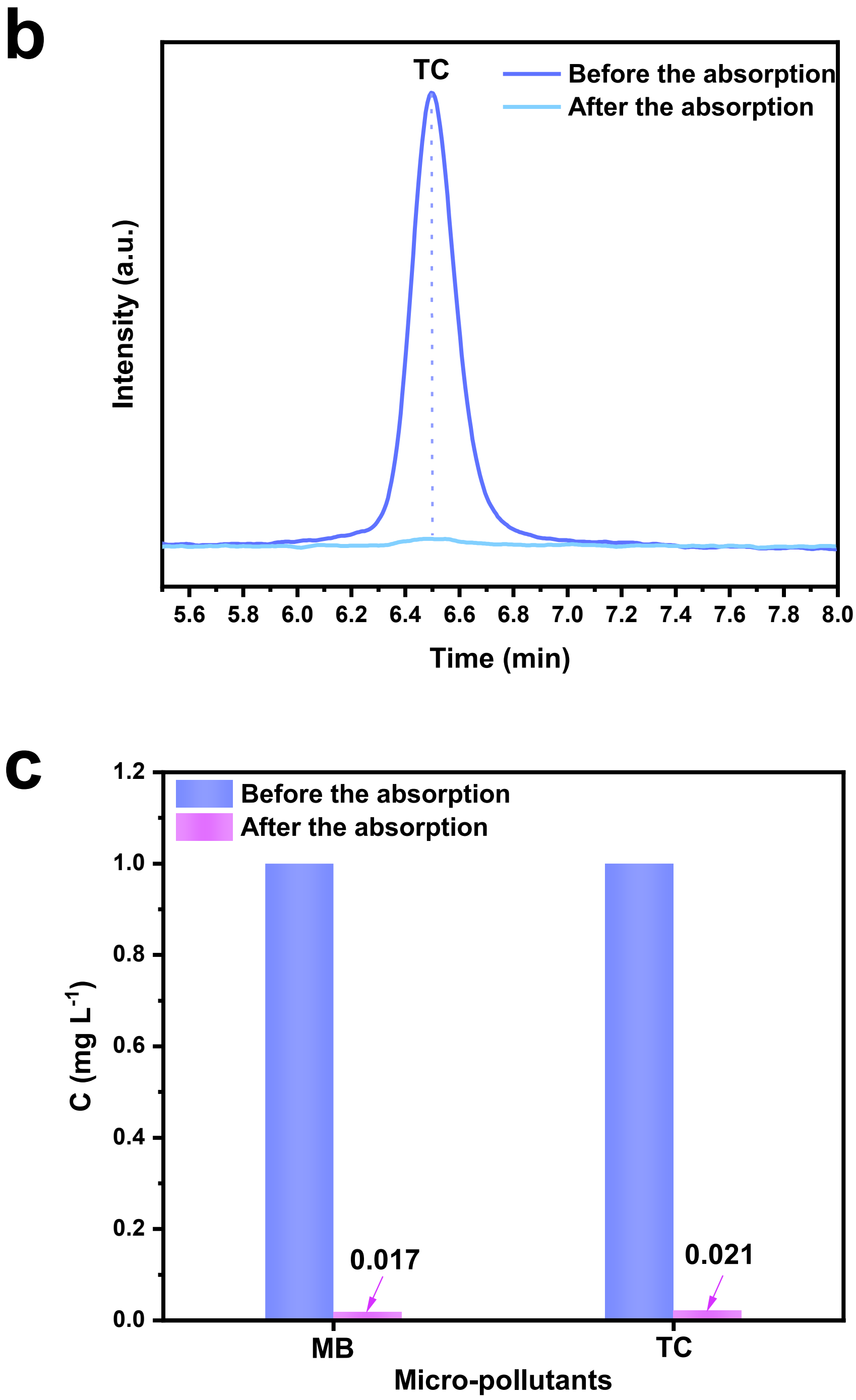

Figure S57. (a) The removal efficiency of PAF-525-9 toward 1 ppm MB by UV spectrophotometric. (b) The removal efficiency of PAF-525-9 toward 1 ppm TC by HPLC chromatogram. (c) Comparison of MB and TC concentrations before and after adsorption.

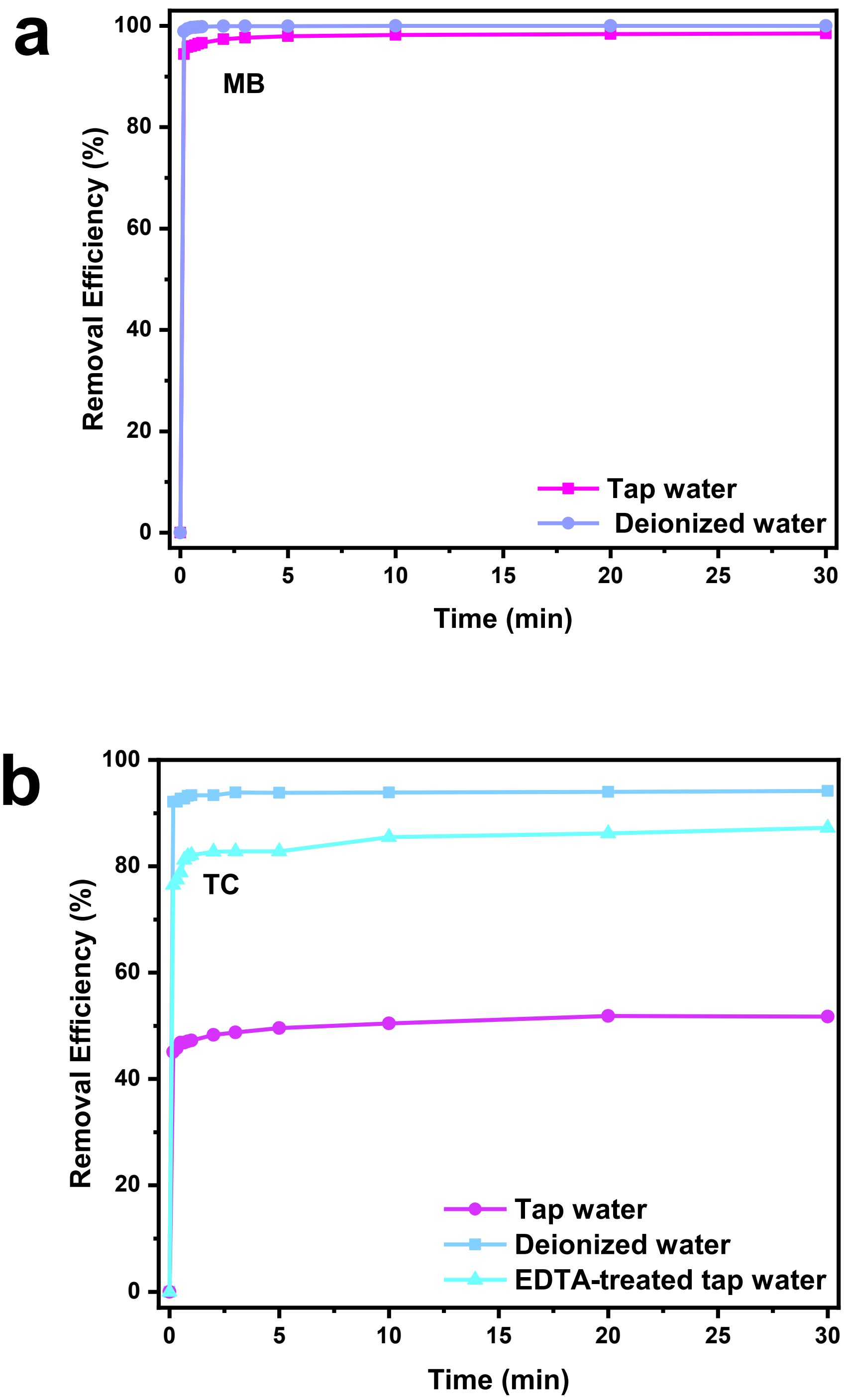
a
MB
Removal Efficiency (%)
Time (min)
Tap water
Deionized water
b
TC
Removal Efficiency (%)
Time (min)
Tap water
Deionized water
EDTA-treated tap water

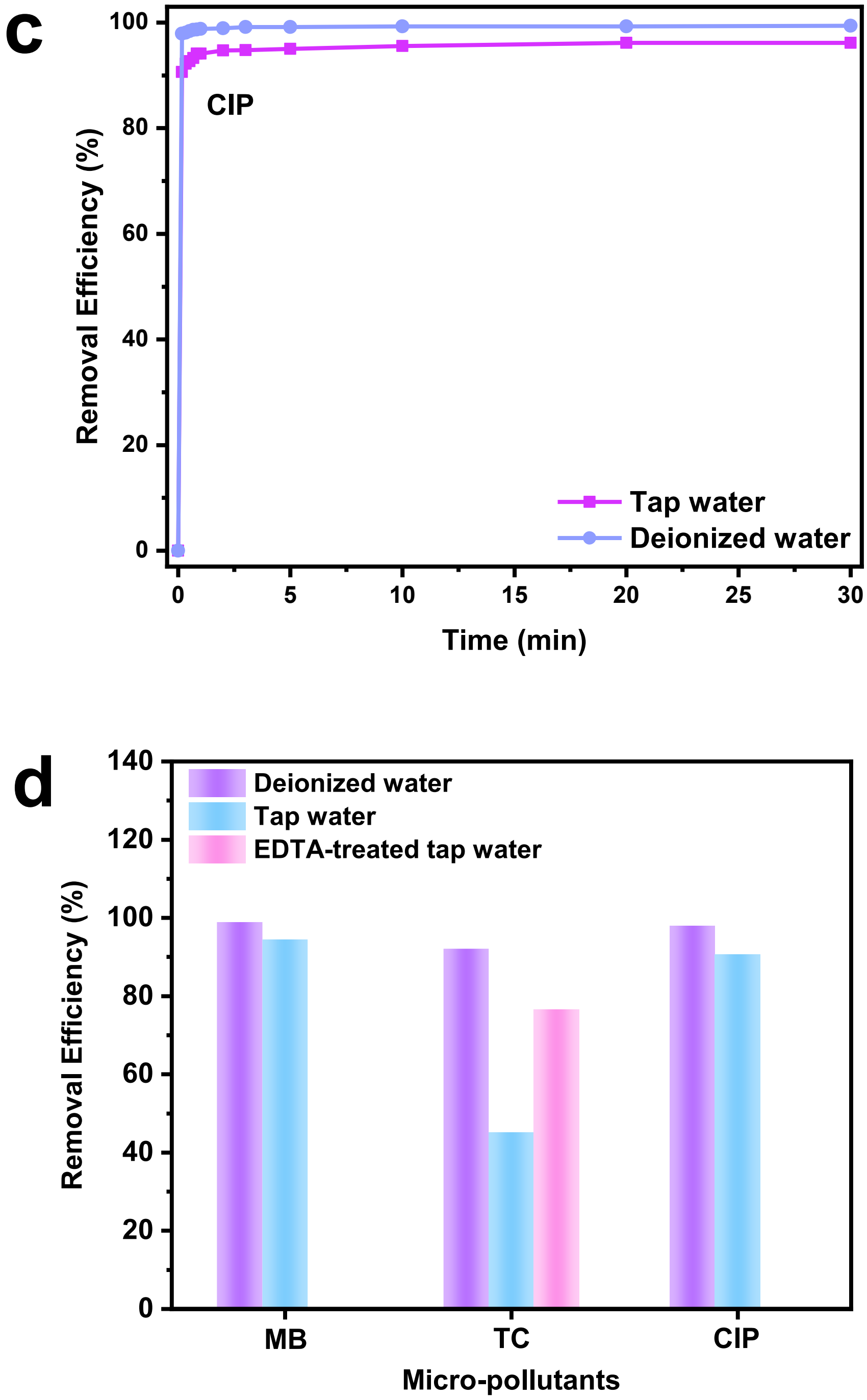

Figure S58. (a) Removal efficiencies of PAF-525-9 toward MB in tap water and deionized water. (b) Removal efficiencies of PAF-525-9 toward TC in tap water, deionized water and EDTA-treated tap water. (c) Removal efficiencies of PAF-525-9 toward CIP in tap water and deionized water. (d) Removal efficiencies of PAF-525-9 toward MB, TC and CIP in tap water and deionized water after 10 seconds.

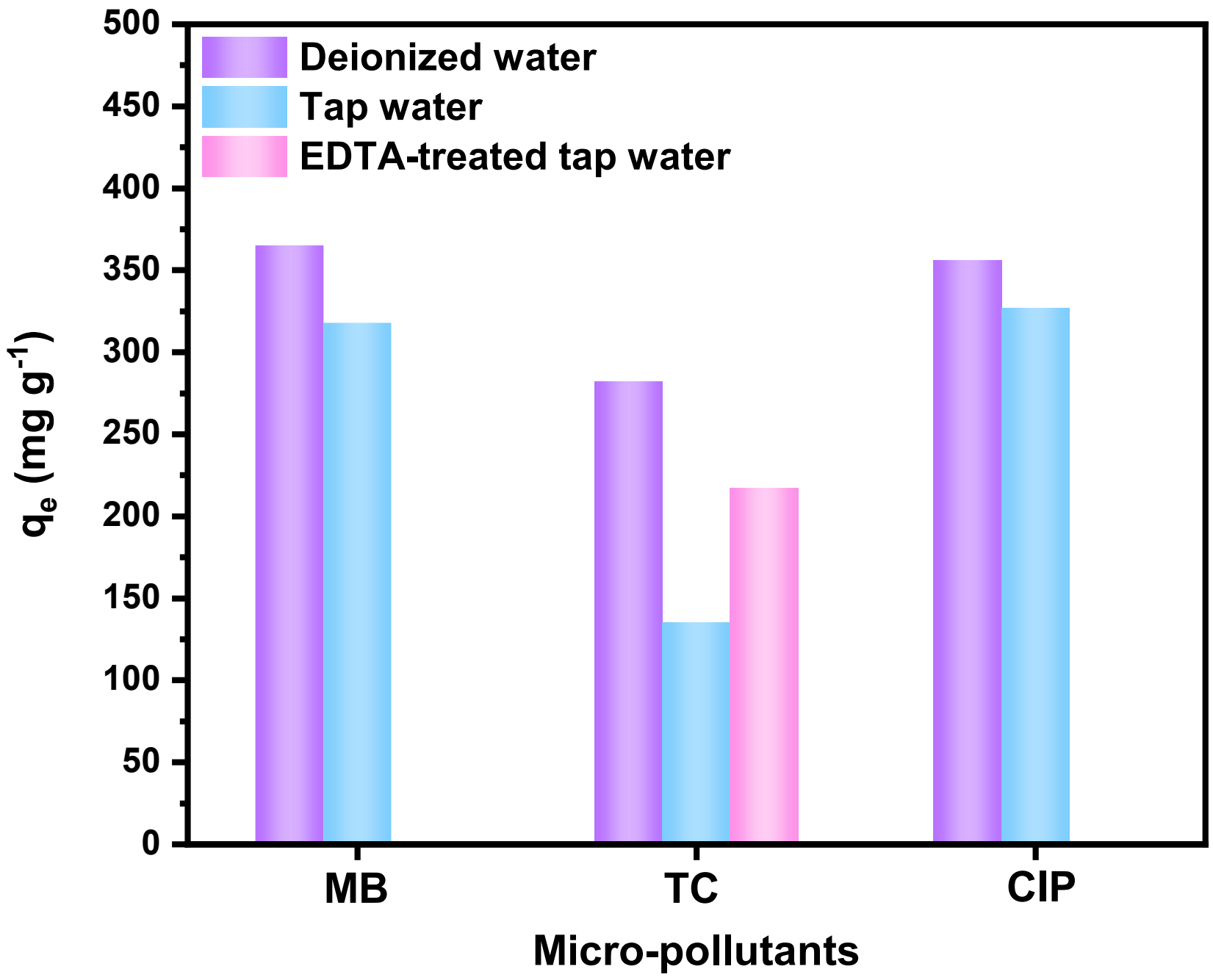

Figure S59. Maximum adsorption capacities of PAF-525-9 toward MB, TC and CIP under different conditions.

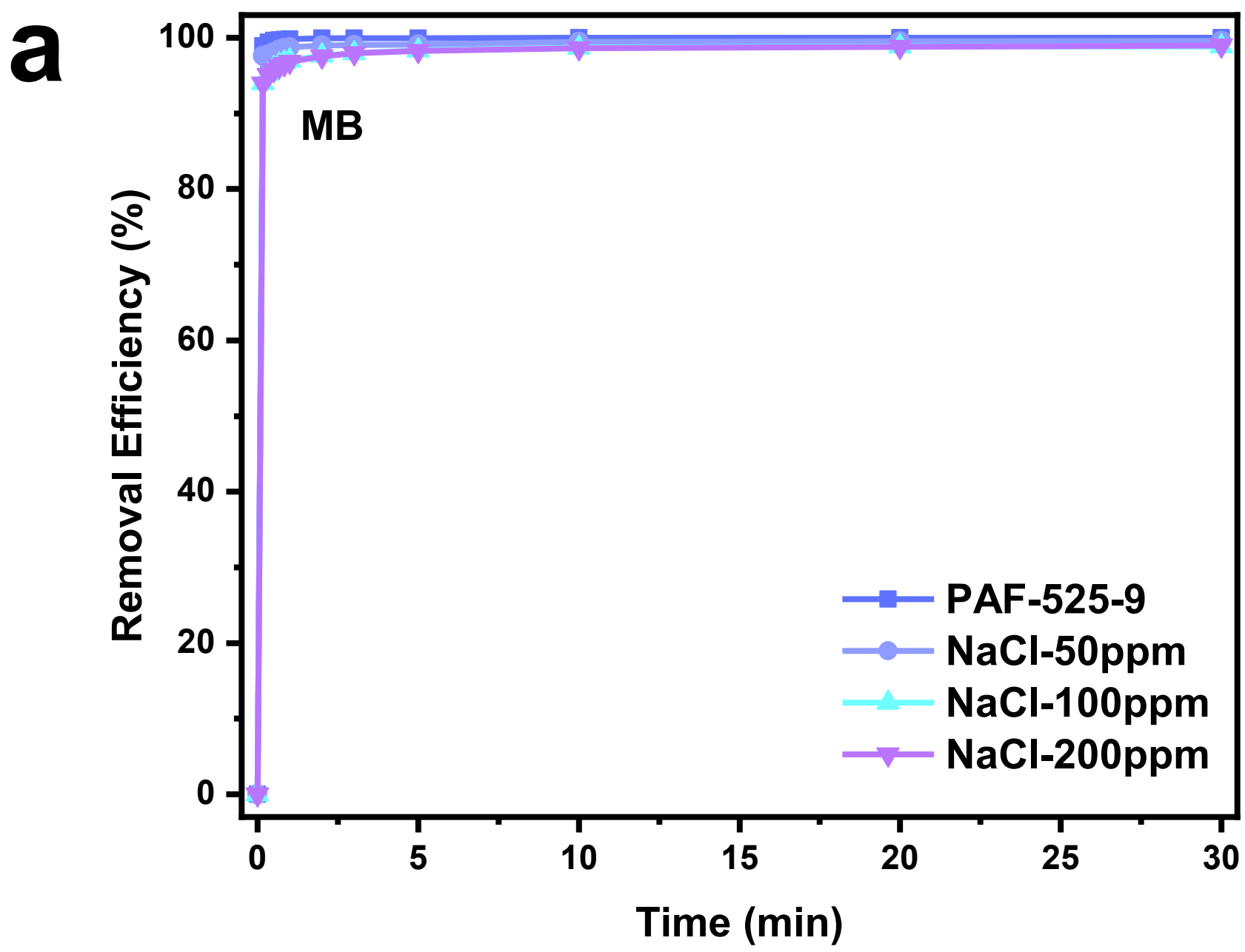

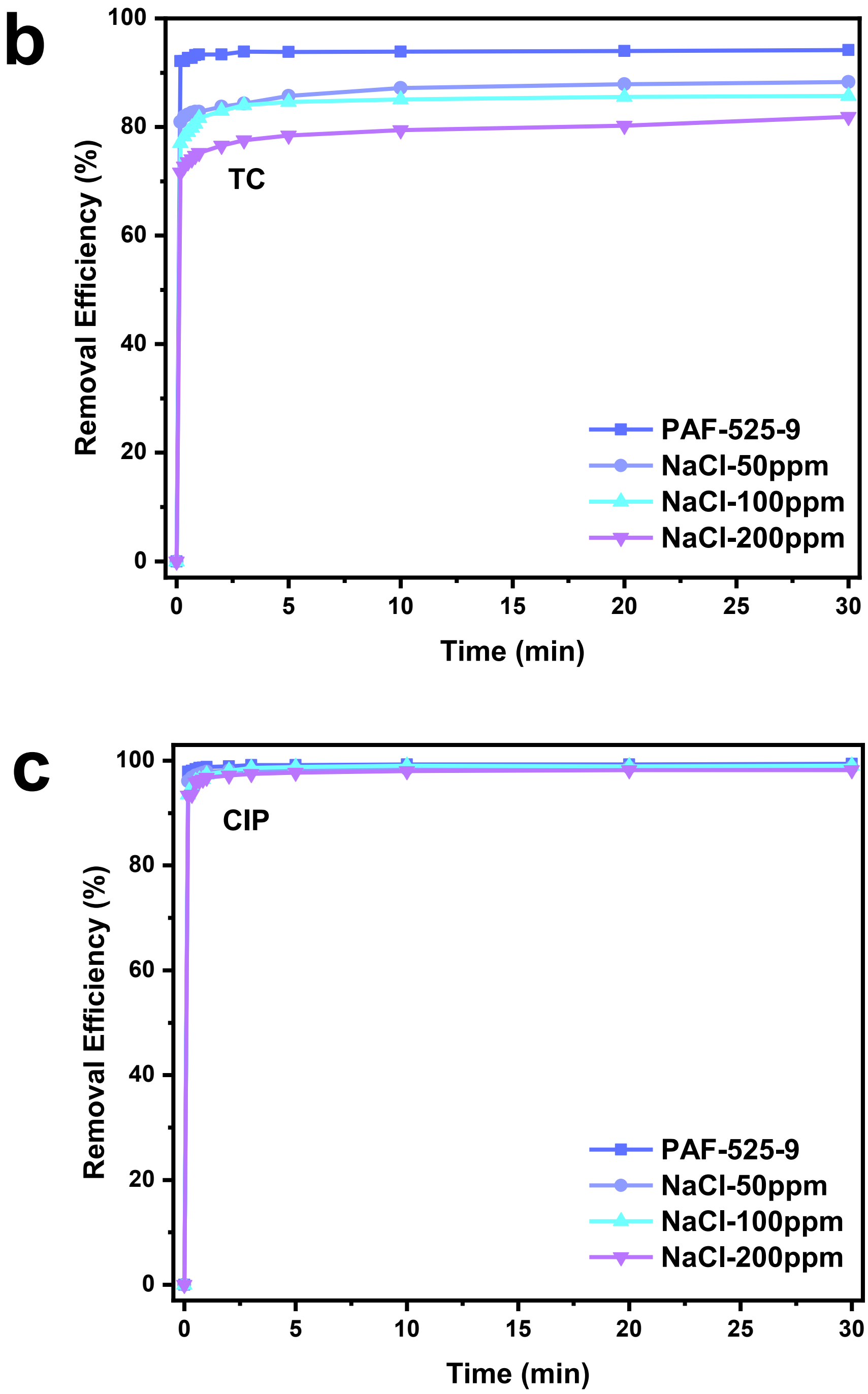
b
TC
Removal Efficiency (%)
Time (min)
PAF-525-9
NaCl-50ppm
NaCl-100ppm
NaCl-200ppm
0
20
40
60
80
100
0
5
10
15
20
25
30
c
CIP
Removal Efficiency (%)
Time (min)
PAF-525-9
NaCl-50ppm
NaCl-100ppm
NaCl-200ppm

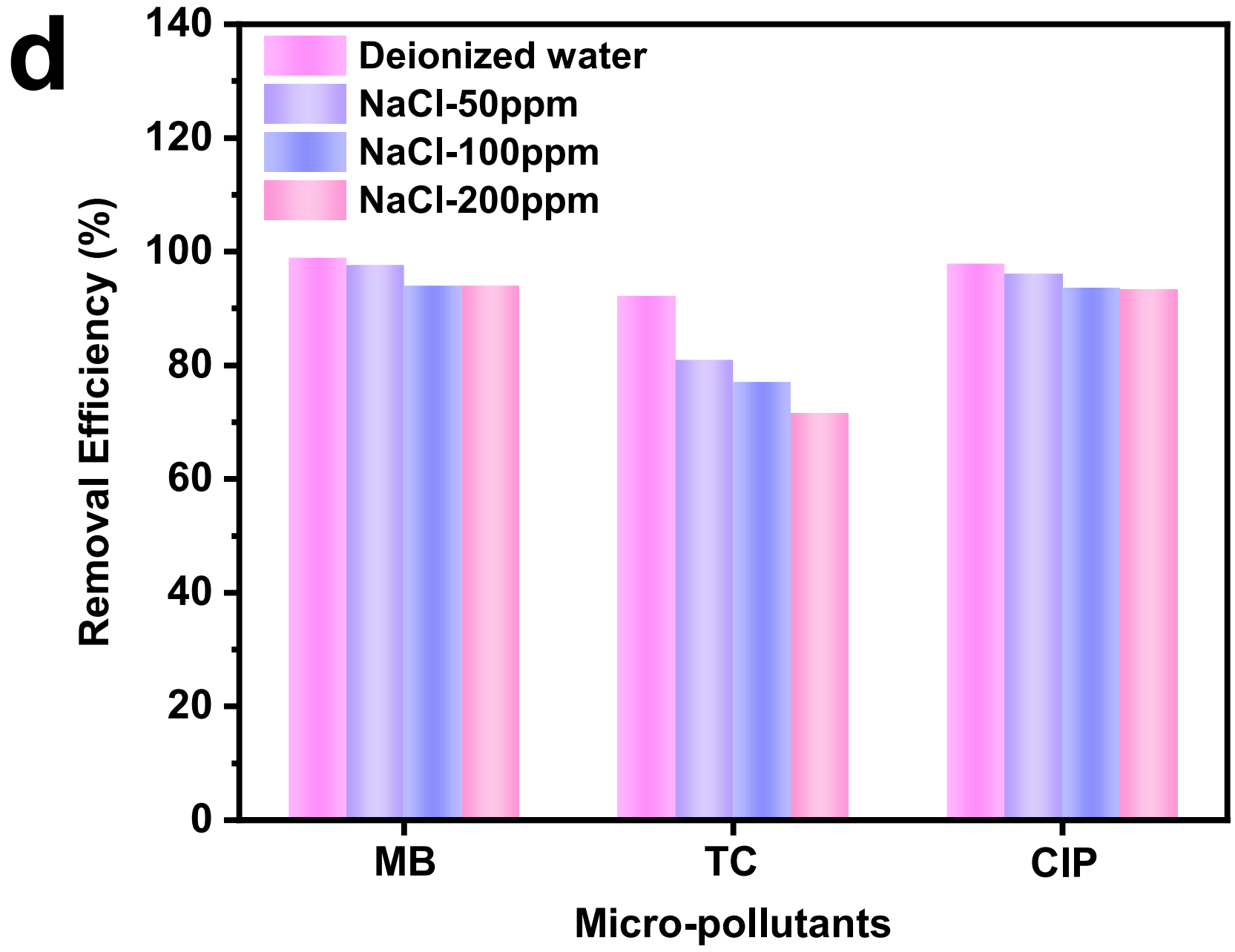

Figure S60. Removal efficiencies of PAF-525-9 toward MB (a), TC (b) and CIP (c) at different sodium ion concentrations. (d) Removal efficiencies of PAF-525-9 toward MB, TC and CIP over 10 seconds at different sodium ion concentrations.

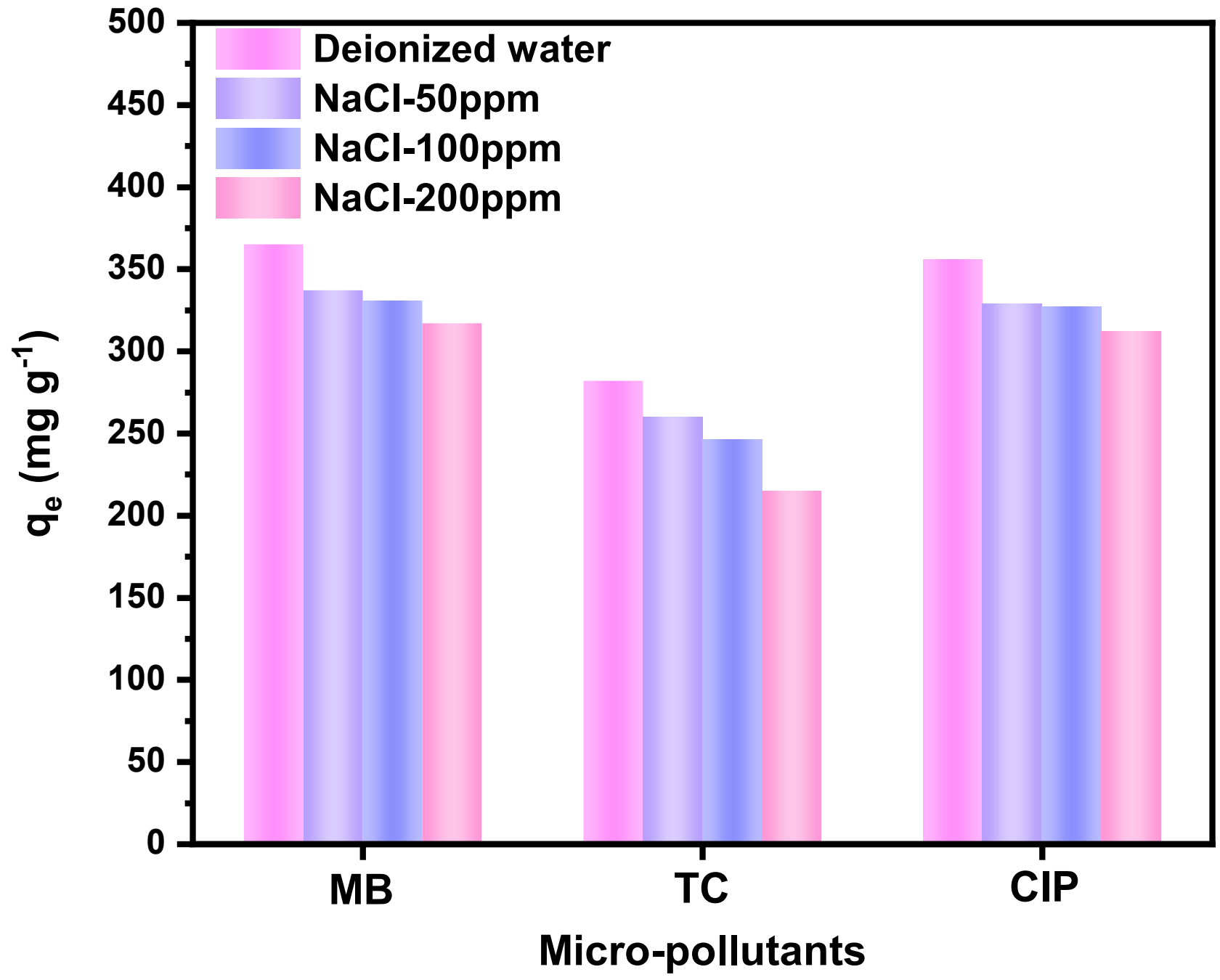

Figure S61. Maximum adsorption capacities of PAF-525-9 toward MB, TC and CIP at different sodium ion concentrations.

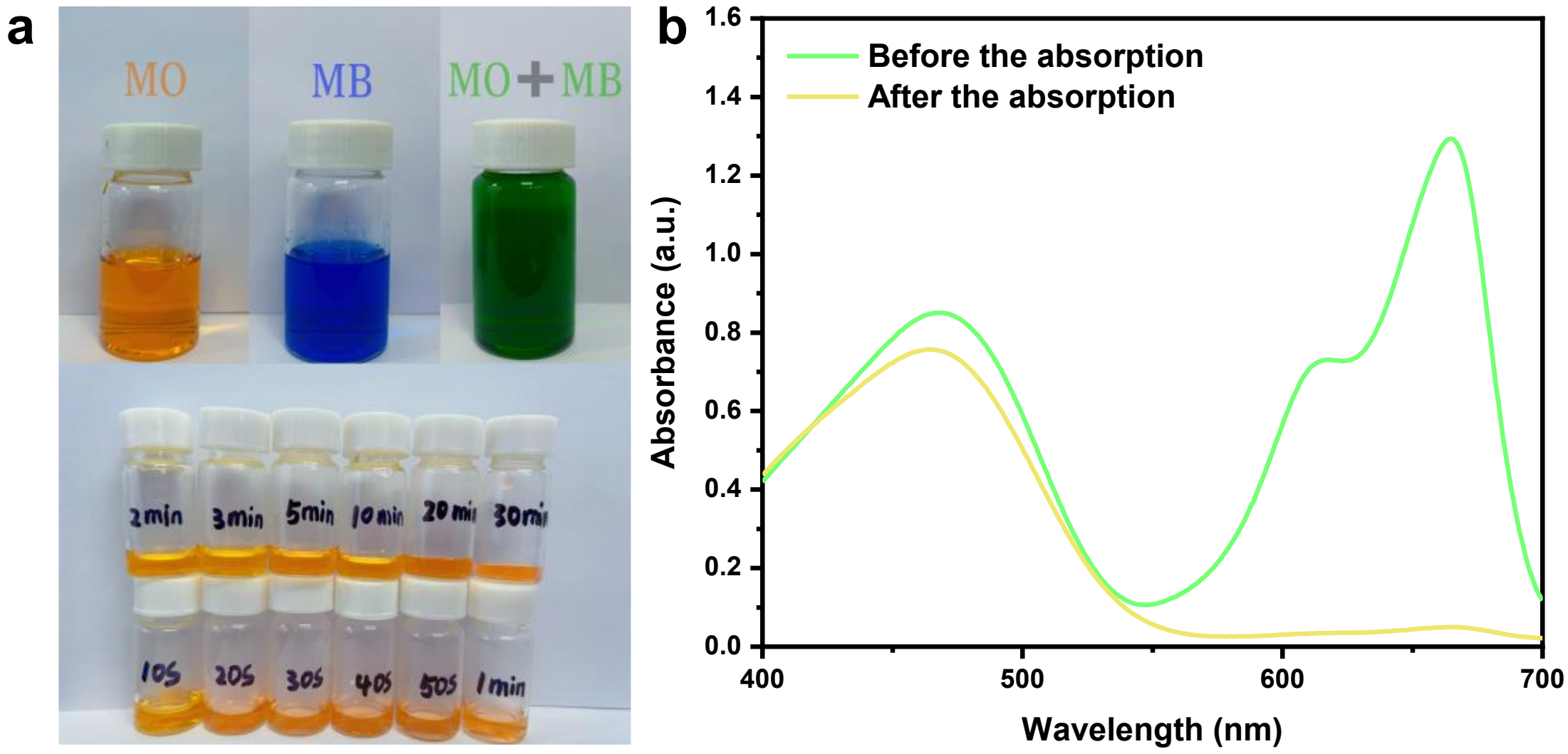

Figure S62. (a) Solution color for methyl orange, methylene blue, the mixture of methyl orange and methylene blue, and the mixture of methyl orange and methylene blue after adsorption. (b) UV spectrophotograms of the solution before and after adsorption.

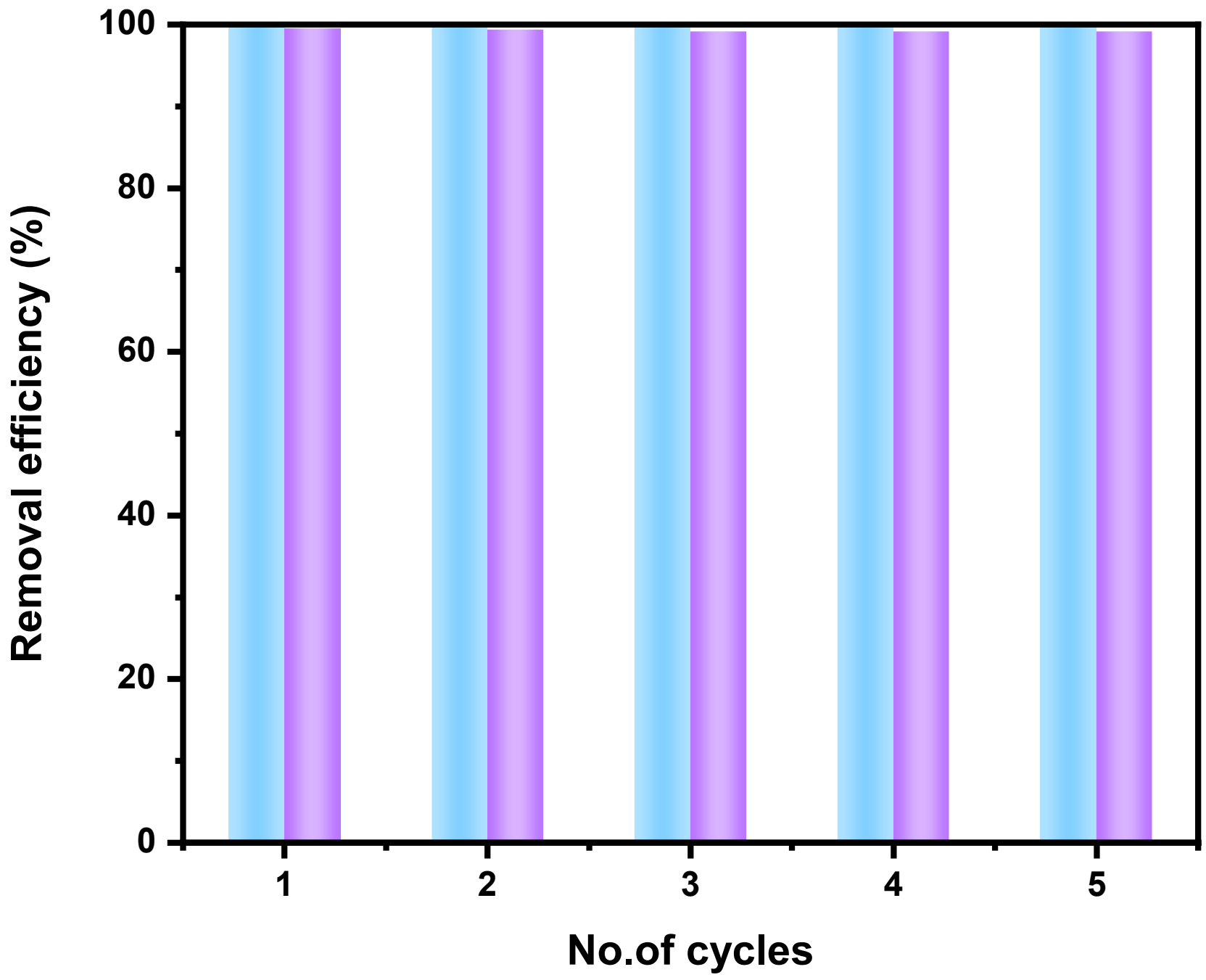

Figure S63. Regeneration performance of PAF-525-9 for micro-pollutants models MB (blue) and TC (purple).

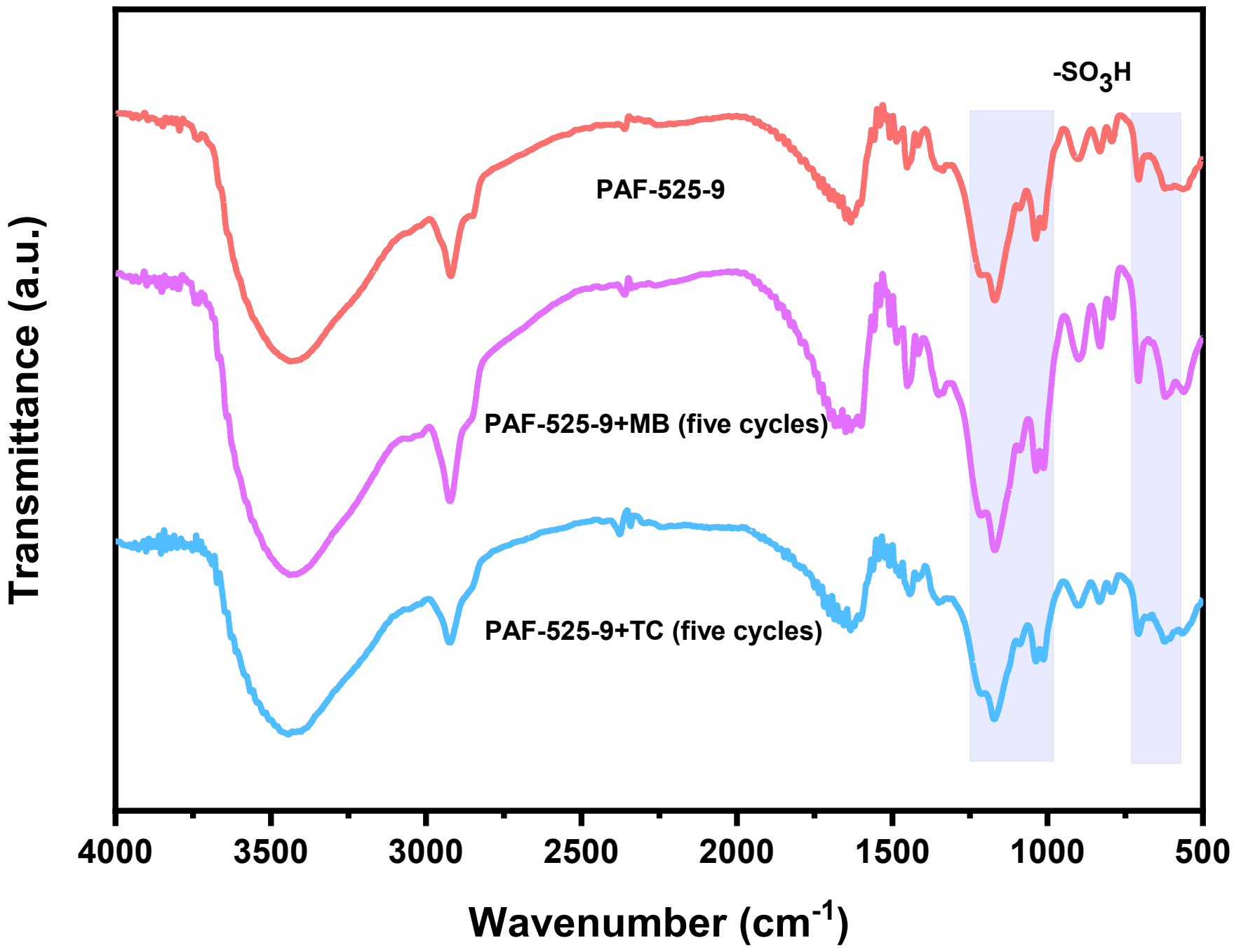

Figure S64. FT-IR spectra of PAF-525-9 after 5 cycles.

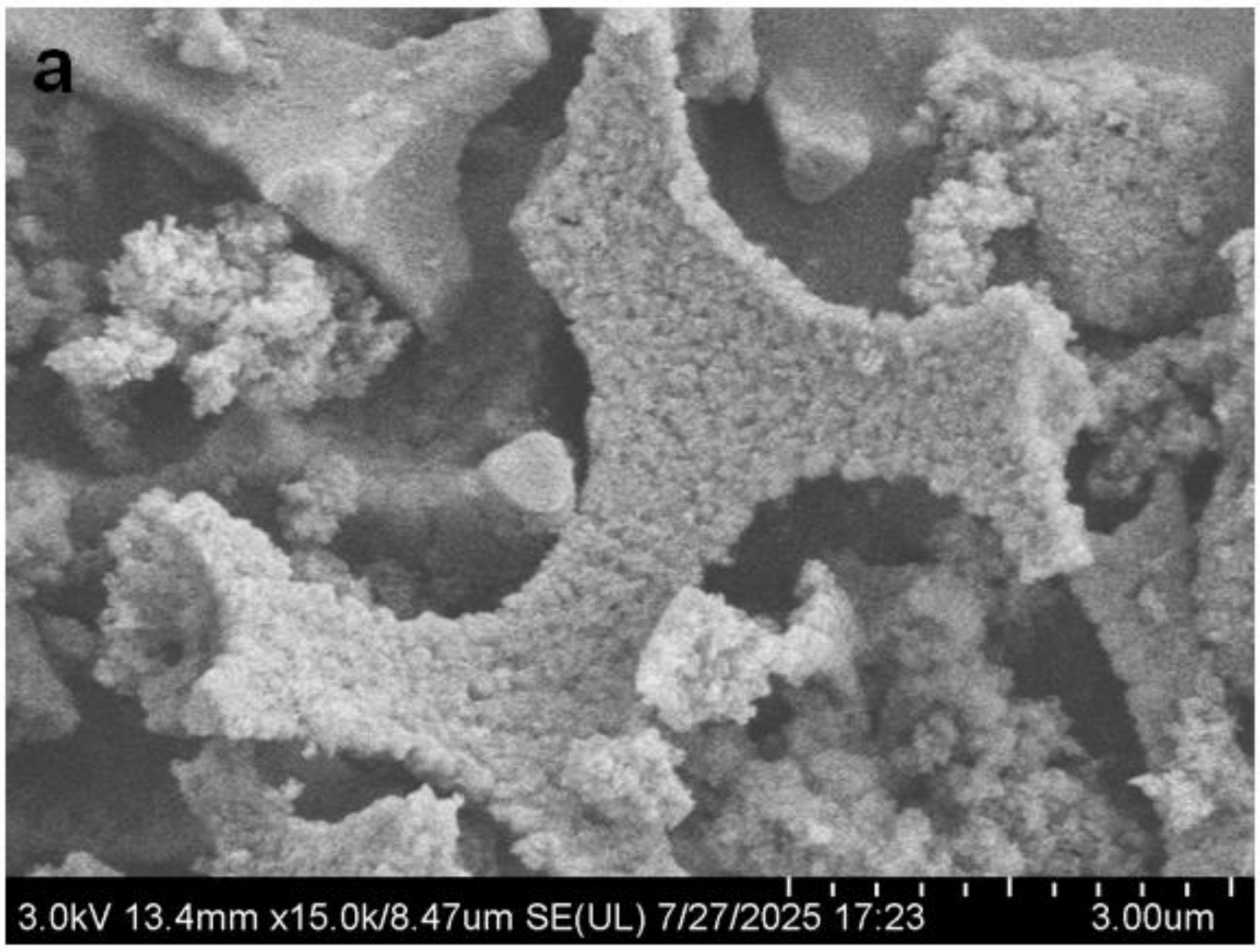

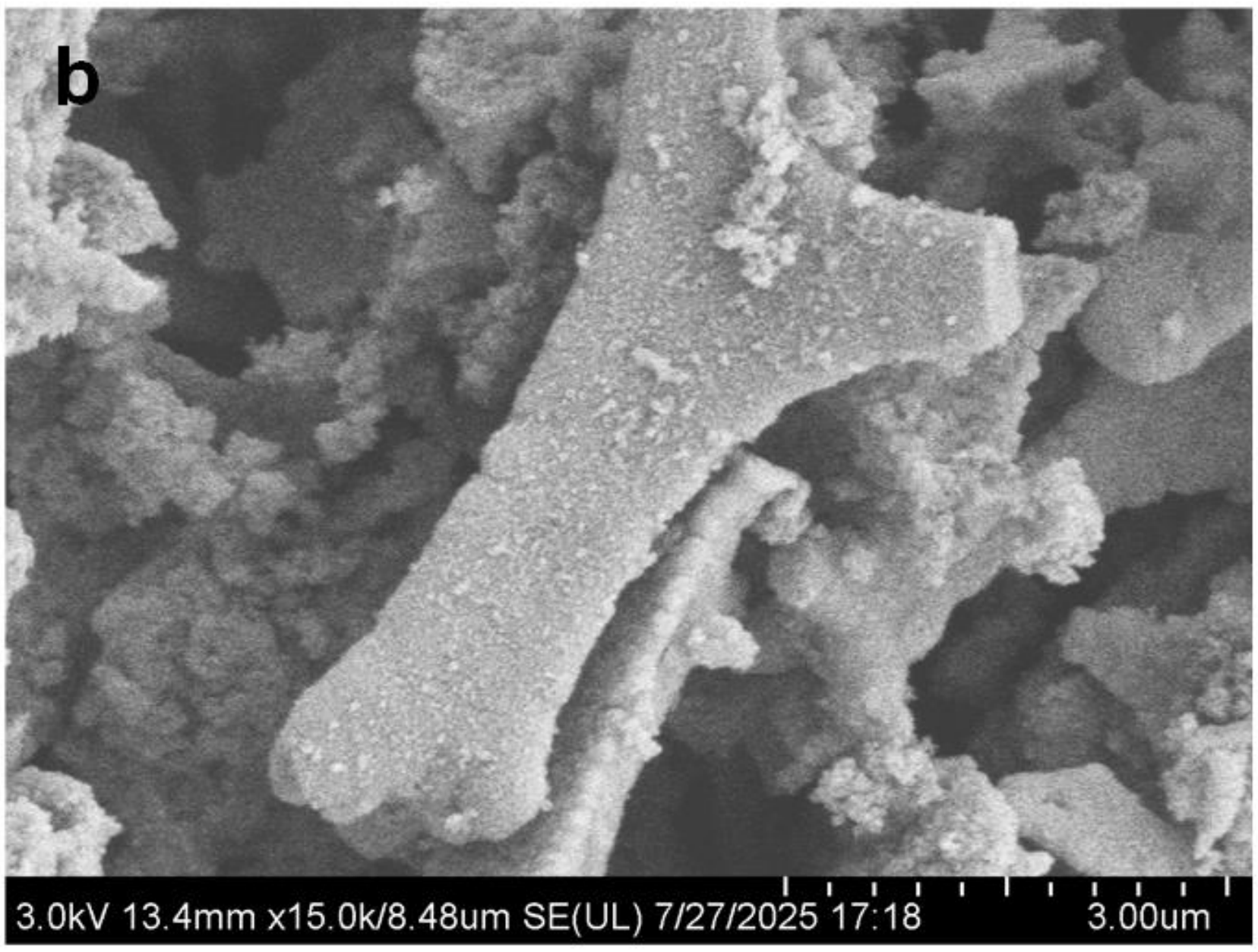

Figure S65. FE-SEM images of PAF-525-9 after 5 cycles for PAF-525-9 + MB (a) and PAF-525-9 + TC (b).

Supporting Tables:

Table S1. Sulfur content of samples.

Samples	Sulfur content (%)
PAF-525-1	9.346
PAF-525-2	9.705
PAF-525-3	9.377
PAF-525-4	9.8
PAF-525-5	9.618
PAF-525-6	10.015
PAF-525-7	10.072
PAF-525-8	10.549
PAF-525-9	10.52
PAF-525-9-50%	8.53
PAF-525-9-25%	6.27

Table S2. Porosity, cumulative pore volume, macropore width and BET surface area of samples.

Samples	Porosity (%)	Pore volume (cm^3 g^{-1})	Pore width (μm)	Surface area (m^2 g^{-1})
PAF-525-1	80.62	4.325	1.636	225
PAF-525-2	83.89	8.812	2.818	232
PAF-525-3	86.38	11.738	3.804	229
PAF-525-4	87.68	15.636	8.053	211
PAF-525-5	88.92	21.843	11.317	217
Meso-PAF-525-4	88.06	16.241	8.061	234

Table S3. BET surface area, micropore volume and micropore width of samples.

Samples	Surface area (m^2 g^{-1})	Micropore volume (cm^3 g^{-1})	Micropore width (nm)
PAF-525-1	206	0.355	1.478
PAF-525-2	214	0.347	1.478
PAF-525-3	248	0.383	1.543
PAF-525-5	217	0.325	1.543
PAM-15-1	288	0.532	1.543
PAM-15-2	386	0.759	1.543
PAM-15-3	423	0.834	1.543
PAM-15-4	486	0.919	1.543
PAM-15-5	504	1.036	1.356
PAF-525-4	211	0.406	1.245
PAF-525-6	246	0.447	1.299
PAF-525-7	284	0.470	1.478
PAF-525-8	303	0.522	1.193

PAF-525-9	337	0.558	1.915
PAF-525-9-50%	374	0.692	1.356
PAF-525-9-25%	402	0.760	1.356
PAF-525-9@MB[a]	85	0.162	4.520
PAF-525-9@TC[a]	91	0.145	1.682

[a] The MB- and TC-adsorbed PAF-525-9 composites were denoted as PAF-525-9@MB and PAF-525-9@TC, respectively.

Table S4. Kinetics parameters of the removal of different micro-pollutants.

Micro-pollutants	Pseudo-first-order model		Pseudo-second-order model	
	K_1 (min^{-1})	R^2	K_2 (g mg^{-1} min^{-1})	R^2
MB[a]	0.55	0.8354	2.09	0.9998
TC[b]	0.88	0.6112	1.53	0.9999

a) The concentration of MB is 200 ppm; b) The concentration of TC is 50 ppm.

Table S5. K_2 value of PAF-525-9 and other porous adsorbents.

Pollutants	Adsorbents	k_2 (g mg^{-1} min^{-1})	Concentration (ppm)	BET (m^2 g^{-1})	Ref.
MB	PAF-525-9	2.09	200	246	This work
	TP-PPOPs-SO_3H	0.0007	300	368	6
	HCPs@Azp	0.0003	100	862	7
	His-CDP-N	0.216	100	562	8
	Fe_3O_4@MIL-101	0.01	30	116	9
	ZM-CTAB-0.36	0.00812	160	608	10
	TZDABA-COF	0.017	10	147	11

	ZrSBA-1.07-40	0.1204	200	845	12
	TPDD-COOH	0.0012	20	174	13
	CaO-FB@GO	0.002	20	112	14
	CaO-ES@GO	0.0002	50	108	14
	Co/C-1000	0.0000993	20	204	15
	COF-SO$_3$H-2.0	0.003981	20	-	16
	POP-T (1:1.5)	0.01	100	1416	17
	2D-COF	0.00914	200	223	18
	COF-SO$_3$Na	2.16	200	179	19
	CGA	2.33	200	149	19
	PAF-525-9	1.53	50	246	This work
	NaY	0.025	120	657	20
	HCP-BPA-Na	0.0099	0.125	24	21
	HCP-MAH	0.0001	30	754	22
	ZIF-8	0.00143	30	1158	23
	Fe-BTC-HB	0.0005	100	109	24
	CM	0.000038	200	1058	25
	NMF/TT-90	0.000765	50	359	26
	NiCo/Fe$_3$O$_4$	0.024	44.44	86	27
TC	ZnO@Zn-MOF	0.007	20	221	28
	TaPa-1	0.00526	50	703	29
	Mxene-S2	0.00012	-	709	30
	AIg@MOF-rGO	0.00428	10	1.6	31
	Fe$_3$O$_4$@PDA-ZIF	0.000106	200	77	32
	MnO$_2$-graphene	0.000482	200	106	33
	SMC	0.005	40	1215	34
	BNC-8	0.00154	50	1413	35
	FTN@PLP	0.00245	100	-	36
	KP-AC	5.6	50	1528	37

Table S6. Intra-particle diffusion parameters of samples.

Micro-pollutants	Samples	Intra-particle diffusion model		
		K_{i1} (mg g^{-1} $min^{-1/2}$)	K_{i2} (mg g^{-1} $min^{-1/2}$)	K_{i3} (mg g^{-1} $min^{-1/2}$)
MB	PAF-525-1	80.5512	0.0277	-
	PAF-525-2	51.5434	0.0502	-
	PAF-525-3	39.5359	0.0030	-
	PAF-525-4	33.1752	0.0218	-
	PAF-525-5	25.2510	0.0043	-
	PAF-525-6	9.0258	1.3224	0.1736
	PAF-525-7	2.1750	0.8123	0.0923
	PAF-525-8	2.7467	0.6994	0.0955
	PAF-525-9	6.0242	0.7001	0.1146
TC	PAF-525-1	1.6311	0.0117	-
	PAF-525-2	1.4906	0.0605	-
	PAF-525-3	0.7350	0.0171	-
	PAF-525-4	0.6830	0.0043	-
	PAF-525-5	0.6074	0.0973	-
	PAF-525-6	1.2991	0.8186	0.0518
	PAF-525-7	1.7113	0.6092	0.0838
	PAF-525-8	2.1466	0.4823	0.0138
	PAF-525-9	1.3784	0.4379	0.0493

Table S7. The parameters of different isotherm models for PAF-525-9.

Samples	Micro-pollutants	Langmuir model			Freundlich model		
		q_m[a] (mg g^{-1})	k_L[b] (L mg^{-1})	R^{2c}	K_F[d] (mg g^{-1})	1/n[e]	R^{2f}
PAF-525-9	MB	361	4.45	0.9996	226	0.12	0.7117
PAF-525-9-50%		337	2.09	0.9995	218	0.11	0.5811
PAF-525-9-25%		315	2.56	0.9995	207	0.10	0.6441
PAF-525-9	TC	294	0.68	0.9995	101	0.24	0.8813
PAF-525-9-50%		230	0.24	0.9975	53	0.35	0.9072

PAF-525-9-25%	195	0.37	0.9976	46	0.33	0.9301

a) The maximum adsorption capacity calculated by the Langmuir model; b) The adsorption capacity constant of the Langmuir model; c) The correlation coefficients of the Langmuir model; d) The adsorbent capacity constant of the Freundlich model; e) The n is the heterogeneity factor and $1/n$ is the adsorption intensity of the Freundlich model; f) The correlation coefficients of the Freundlich model.

Table S8. Kinetics parameters of the removal of different micro-pollutants at different temperatures.

Micro-pollutants	Temperature	Pseudo-second-order model	
		K_2 (g mg^{-1} min^{-1})	R^2
MB	273.15	0.02	0.9998
	298.15	2.09	0.9998
	318.15	2.92	0.9998
TC	273.15	0.23	0.9998
	298.15	1.53	0.9999
	318.15	1.82	0.9998

Table S9. Electronic energy and enthalpy value of samples calculated by DFT.

Samples	E (kJ mol^{-1})	H (kJ mol^{-1})
PAF-525-9	-7158.604407	-7158.506546
MB	-1562.972388	-1562.942654
TC	-1182.072362	-1182.052826
MB/PAF-525-9	-8721.631474	-8721.505033
TC/PAF-525-9	-8340.737807	-8340.621336

References:

1. Y. Zhao, D. G. Truhlar, The M06 suite of density functionals for main group thermochemistry, thermochemical kinetics, noncovalent interactions, excited states, and transition elements: two new functionals and systematic testing of four M06-class functionals and 12 other functionals, T*heor. Chem. Acc.,* 2008, **120**, 215.

2. T. Lu, A comprehensive electron wavefunction analysis toolbox for chemists, *J. Chem. Phys.,* 2024, **161**, 41.

3. T. Lu, F. W. Chen, Multiwfn: A multifunctional wavefunction analyzer, *J. Comput. Chem.,* 2012, **33**, 580.

4. T. Lu, Q. X. Chen, Independent gradient model based on Hirshfeld partition: A new method for visual study of interactions in chemical systems, *J. Comput. Chem.,* 2022, **438**, 539.

5. J. Zhang, T. Lu, Efficient evaluation of electrostatic potential with computerized optimized code, *Phys. Chem. Chem, Phys.,* 2021, **23**, 20323.

6. Y. C. Zhu, R. Ding, S. H. Chen, X. Y. Qu, Y. Z. Yang, X. M. Zhang, Three-dimensional sulfonic-functionalized porphyrin-based porous organic polymer for high-performance methylene blue and ciprofloxacin capture, *Sep. Purif. Technol.,* 2024, **333**, 12.

7. A. T. Liu, X. M. Wei, S. L. Lin, L. Ding, Y. Huang, X. F. Fan, P. Li, C. H. Lau, Photo-modulated regeneration of hypercrosslinked polymer adsorbents for water treatment, *Sep. Purif. Technol.,* 2024, **329**, 13.

8. K. Zhang, Y. L. Shi, Z. X. Zhang, L. J. Li, J. T. Wang, N. Y. Peng, G. F. Dai, Y. F. He, F. Xia, X. J. Zhang, Rapid removal of various recalcitrant pollutants from water by a pH-responsive high-swelling cyclodextrin polymer, *Chem. Eng. J.,* 2025, **515**, 10.

9. H. S. Far, M. Hasanzadeh, M. Najafi, M. Rabbani, Magnetic metal-organic framework (Fe3O4@MIL-101) functionalized with dendrimer: highly efficient and selective adsorption removal of organic dyes, *J. Inorg. Organomet. Polym. Mater.,* 2022, **32**, 3848-3863.

10. H. O. Wang, W. D. Chen, S. F. Yan, C. X. Guo, W. Ma, A. Yang, Zirconium-doped MCM-41 from Zirconium-containing silica residue for methylene blue removal: one-pot hydrothermal synthesis, characterization, and dye adsorption performance, *Sep. Purif. Technol.,* 2025, **376**, 14.

11. R. Li, K. Zhang, X. Yang, R. Xiao, Y. Xie, X. H. Tang, G. Miao, J. Fan, W. G. Zhang, S. R. Zheng, S. L. Cai, Construction of a carboxyl-functionalized clover-like covalent organic framework for selective adsorption of organic dyes, *Sep. Purif. Technol.,* 2024, **340**, 10.

12. H. O. Wang, W. D. Chen, S. F. Yan, C. X. Guo, W. Ma, Synthesis of zirconium-doped SBA-15 from zirconium-containing silica residue via an in situ one-pot hydrothermal method for methylene blue removal: structural characterization and adsorption performance, *Sep. Purif. Technol.,* 2025, **378**, 16.

13. G. S. Hu, G. Cui, J. Zhao, M. Y. Han, R. Y. Zou, Pyrazine-cored covalent organic frameworks for efficient CO_2 adsorption and removal of organic dyes, *Polym. Chem.,* 2022, **13**, 3827-3832.

14. K. S. Obayomi, S. Y. Lau, M. K. Danquah, J. H. Zhang, T. Chiong, O. V. Obayomi, L. Meunier, M. M. Rahman, A response surface methodology approach for the removal of methylene blue dye from wastewater using sustainable and cost-effective adsorbent, *Process Saf. Environ. Protect.,* 2024, **184**, 129-150.

15. X. F. Ma, S. Y. Zhao, Z. W. Tian, G. G. Duan, H. Y. Pan, Y. Y. Yue, S. S. Li, S. J. Jian, W. S. Yang, K. M. Liu, S. J. He, S. H. Jiang, MOFs meet wood: reusable magnetic hydrophilic composites toward efficient water treatment with super-high dye adsorption capacity at high dye concentration, *Chem. Eng. J.,* 2022, **446**, 9.

16. C. H. Ding, Y. Y. Du, S. Agarwal, Open-cell robust COF-nanowire network sponges as sustainable adsorbent and filter, *Adv. Funct. Mater.,* 2024, **34**, 10.

17. N. Li, L. L. Huo, W. J. Shen, C. H. Qiang, M. M. Wu, G. F. Sun, Q. F. Li, M. H. Shi, J. Q. Ma, Porous organic polymers for superefficient removal of pollutants from water: Design, synthesis and adsorption performance, *J. Clean Prod.,* 2023, **396**, 9.

18. C. D. Zhou, H. Y. Zhou, S. Y. Tang, F. M. Zhang, S. Lei, Z. J. Li, M. Chen, J. J. Liu, Aryl-aryl linked two-dimensional covalent organic frameworks/cellulose composite monolith with hierarchical structure for aqueous dyes adsorption, *J. Appl. Polym. Sci.,* 2022, **139**, 10.

19. C. Li, P. Guggenberger, S. W. Han, W. L. Ding and F. Kleitz, Ultrathin covalent organic framework anchored on graphene for enhanced organic pollutant removal, *Angew. Chem. Int. Ed.,* 2022, **61**, e202206564.

20. M. M. M. Ali, M. J. Ahmed, B. H. Hameed, NaY zeolite from wheat (Triticum aestivum L.) straw ash used for the adsorption of tetracycline, *J. Clean Prod.,* 2018, **172**, 602-608.

21. A. B. Hu, L. Liqing, M. Y. Zhang, Y. Y. Liu, G. Y. Liao, D. S. Wang, Synthesis of highly water-dispersible adsorbent derived from alkali-modified hyper-cross-linked polymer for efficient removal of various organic contaminants and ammonia, *J. Water Process. Eng.,* 2021, **40**, 12.

22. S. W. Song, J. Ai, A. B. Hu, G. Y. Liao, D. S. Wang, Synthesis of carboxyl-modified hyper-cross-linked polymers with conspicuous removal capability for various water-soluble contaminants, *J. Environ. Chem. Eng.,* 2021, **9**, 15.

23. N. Li, L. Zhou, X. Y. Jin, G. Owens, Z. L. Chen, Simultaneous removal of tetracycline and

oxytetracycline antibiotics from wastewater using a ZIF-8 metal organic-framework, *J. Hazard. Mater.,* 2019, **366**, 563-572.

24. C. F. Zheng, Q. Wu, X. J. Hu, J. X. Ma, K. Y. Sun, Y. J. Sun, B. C. Xu, Macro-manufacturing robust and stable metal-organic framework beads for antibiotics removal from wastewater, *Environ. Res.,* 2024, **246**, 13.

25. W. Sebai, S. Ahmad, N. Brun, T. Cacciaguerra, D. Cot, A. Boccheciampe, P. Chaurand, C. Levard, M. P. Belleville, J. Sanchez-Marcano, A. Galarneau, Treatment of wastewater containing pharmaceutical micropollutants by adsorption under flow in highly porous carbon monoliths, *Chem. Mater.,* 2023, **35**, 8464-8482.

26. N. Yuan, C. Zhang, X. L. Zhang, R. H. Zhang, Covalent integration of Fe-based metal-organic framework and triazine-containing covalent organic framework for enhanced adsorptive removal of antibiotics, *J. Clean Prod.,* 2024, **434**, 10.

27. R. Xiao, H. I. Abdu, L. P. Wei, T. Y. Wang, S. H. Huo, J. Chen, X. Q. Lu, Fabrication of magnetic trimetallic metal-organic frameworks for the rapid removal of tetracycline from water, *Analyst.,* 2020, **145**, 2398-2404.

28. Z. Q. Guo, F. J. Yang, R. R. Yang, L. Sun, Y. Li, J. Z. Xu, Preparation of novel ZnO-NP@Zn-MOF-74 composites for simultaneous removal of copper and tetracycline from aqueous solution, *Sep. Purif. Technol.,* 2021, **274**, 11.

29. L. J. Huang, N. Q. Mao, Q. Shuai, Efficient removal of tetracycline from aqueous solution by covalent organic frameworks derived porous carbon, *J. Environ. Chem. Eng.,* 2021, **9**, 8.

30. R. C. Wang, C. Yao, C. Y. Peng, J. F. Qiu, Q. Wang, X. Liu, J. L. Meng, W. Wang, Ultra-strong adsorption of organic dyes and antibiotic onto the alk-MXene/ ZIF adsorbents with a specific intercalation structure, *Chem. Eng. J.,* 2024, **485**, 14.

31. N. Kim, B. Cha, Y. Yea, L. K. Njaramba, S. Vigneshwaran, S. S. D. Elanchezhiyan, C. M. Park, Effective sequestration of tetracycline and ciprofloxacin from aqueous solutions by Al-based metal organic framework and reduced graphene oxide immobilized alginate biosorbents, *Chem. Eng. J.,* 2022, **450**, 14.

32. D. H. Sheng, X. T. Ying, R. Li, S. Y. Cheng, C. Zhang, W. Dong, X. H. Pan, Polydopamine-mediated modification of ZIF-8 onto magnetic nanoparticles for enhanced tetracycline adsorption from wastewater, *Chemosphere.,* 2022, **308**, 9.

33. Z. Song, Y. L. Ma, C. E. Li, The residual tetracycline in pharmaceutical wastewater was effectively removed by using MnO_2 graphene nanocomposite, *Sci. Total Environ.,* 2019, **651**, 580-590.

34. B. Wang, Q. Y. Mo, B. Qin, L. Song, J. Li, G. S. Sheng, D. Z. Shi, X. Y. Xu, L. A. Hou, Adsorption behaviors of three antibiotics in single and co-existing aqueous solutions using mesoporous carbon, *Environ. Res.,* 2022, **215**, 12.

35. H. Li, N. Wang, C. X. Lin, Z. Q. Ren, Y. C. Du, Q. Xu, B, N dual-doped porous carbon nano frameworks as bifunctional peroxymonosulfate activators for ultrafast adsorption and decomposition of microcontaminants in water, *Chem. Eng. J.,* 2025, **503**, 14.

36. H. P. Zeng, J. H. Wu, Y. Chen, J. R. Liang, S. M. Li, S. Han, D. Li, J. Zhang, Adsorptive removal of tetracycline from wastewater using platanus leaf powder modified by metal-phenolic network based on Fe (III)-Tannic acid: Low carbon and sustainable, *Sep. Purif. Technol.,* 2025, **379**, 13.

37. H. O. Singh, G. Murugesan, T. Varadavenkatesan, R. Selvaraj and R. Vinayagam, High surface area activated carbon for sustainable tetracycline adsorption: Mechanism, regeneration and efficacy in realistic water matrices, *Diamond and Relat. Mater.,* 2025, **159**, 112835.
